# E-FAST Ultrasound Training Curriculum for Prehospital Emergency Medical Service (EMS) Clinicians

**DOI:** 10.21980/J8S060

**Published:** 2024-01-31

**Authors:** Clever M. Nguyen, Krista Hartmann, Craig Goodmurphy, Avram Flamm

**Affiliations:** * Pennsylvania State University College of Medicine, Hershey, PA; ^ Pennsylvania State University College of Medicine, Department of Radiology, Hershey, PA; † WellSpan Health, Department of Emergency Medicine, York, PA; ** WellSpan EMS, York, PA

## Abstract

**Audience and Type of Curriculum:**

Audience and type of curriculum: This hybrid, asynchronous curriculum is designed for prehospital clinician colleagues, including but not limited to emergency medical technicians (EMT), advanced EMTs (AEMT), EMT-paramedics (EMT-P), critical care EMT-Ps (CCEMTP), critical care transport nurses (CCTN), and certified flight registered nurses (CFRN) to learn and practice ultrasound fundamentals in the setting of a standardized extended focused assessment with sonography in trauma (E-FAST) exam.

**Length of Curriculum:**

Over a five-month curriculum, learners will perform a pre-test, review online module lectures, attend an ultrasound scanning workshop, and perform post-test examinations.

**Introduction:**

The extended-focused assessment with sonography in trauma (E-FAST) exam can identify intrathoracic and intraabdominal free fluid, as well as pneumothoraces. The E-FAST ultrasound exam has previously been taught to clinicians of various backgrounds in healthcare including emergency medical service (EMS). However, an open-access, systemized curriculum for teaching E-FAST exams to EMS clinicians has not been published.

**Educational Goals:**

By the end of these training activities, prehospital EMS learners will be able to demonstrate foundational ultrasound skills in scanning, interpretation, and artifact recognition by identifying pertinent organs and anatomically relevant structures for an E-FAST examination. Learners will differentiate between normal and pathologic E-FAST ultrasound images by identifying the presence of free fluid and lung sliding. Learners will also explain the clinical significance and application of detecting free fluid during an E-FAST scan.

**Educational Methods:**

The educational strategies used in this curriculum include a hybrid, asynchronous curriculum encompassing 2.5 hours of lectures derived from online learning modules and in-person review. In addition, learners will attend 2 hours of hands-on proctored ultrasound scanning practicing E-FAST examinations.

**Research Methods:**

An online 13-question pre-test was administered prior to the study. An online post-test and in-person scanning OSCEs were administered at least eight weeks after their scheduled workshop consisting of an online 13-question multiple-choice post-test, a confidence survey, and a hands-on E-FAST Objectively Structured Clinical Exam (OSCE) session. A non-parametric Wilcoxon signed-rank test was performed between each pre-test and post-test metric to examine the statistical differences of paired data.

**Results:**

Post-test scores demonstrated statistically significant improvement in both image interpretation exams and ultrasound self-efficacy from the pre-test. The mean pre-test and post-test scores were 55.46% (7.21 ± 1.99) and 84.23% (10.89 ± 1.59) correct out of 13 questions, respectively (p < 0.0001). Participants surveyed an increase in self-efficacy reflected by a Likert scale for ultrasound usage and image interpretation (p < 0.005). The average post-test OSCE E-FAST exam score was 37.89 ± 2.76 out of 42 points (90.21%).

**Discussion:**

This 4.5-hour hybrid asynchronous model demonstrates an effective curriculum for teaching E-FAST ultrasound to prehospital clinicians.

**Topics:**

Ultrasound, sonography, prehospital clinicians, emergency medical services (EMS), paramedics, critical care transport, extended focused assessment with sonography in trauma (E-FAST), free fluid, sliding lung sign, elective, pain.

## USER GUIDE

[Table t1-jetem-9-1-c41]

**Table t1-jetem-9-1-c41:** 

List of Resources:	
Abstract	41
User Guide	43
Curriculum Chart	47
Appendix A: Pre-Screening Survey, Pre-Test and Answer Key	54
Appendix B: Introduction	71
Appendix C & D: Ultrasound Machine & Mechanics Part 1	72
Appendix E & F: Ultrasound Machine & Mechanics Part 2	73
Appendix G & H : Subxiphoid-Pericardial View	74
Appendix I & J: Anterior Thoracic View	75
Appendix K & L: Right Upper Quadrant View	76
Appendix M & N: Left Upper Quadrant View	77
Appendix O & P: Suprapubic View	78
Appendix Q: E-FAST Proctored Scanning Workshop Guide	79
Appendix R: Post-Screening Survey, Post-Test and Answer Key	81
Appendix S: OSCE Rubric	96

**Learner Audience:**

Prehospital clinicians, emergency medical service personnel, emergency medical technicians, paramedics, Critical Care Transport registered nurses

**Length of Curriculum:**

Over the course of a five-month curriculum, learners will perform a pre-test, review online module lectures, attend ultrasound scanning workshops, and perform post-test examinations.

**Topics:**

Ultrasound, sonography, prehospital clinicians, emergency medical services (EMS), paramedics, Critical Care Transport, extended focused assessment with sonography in trauma (E-FAST), free fluid, sliding lung sign.

**Objectives:**

By the end of these training activities, prehospital emergency medical service learners will be able to:

1. Demonstrate foundational ultrasound skills in E-FAST scanning, interpretation, and artifact recognition.
2. Acquire clinically adequate sonography images to identify pertinent organs and anatomically relevant structures for an E-FAST examination.
3. Differentiate between normal and pathologic E-FAST ultrasound images by accurately identifying the presence of free fluid in the views captured by E-FAST.
4. Identify the presence of a lung sliding sign in both Bright (B) and Motion (M)-Mode settings.
5. Explain the clinical significance and application of finding free fluid during an E-FAST scan.

### Brief introduction

The use of prehospital ultrasound has been shown to contribute to the initial evaluation, diagnosis, management, and clinical outcomes in patients within the prehospital setting.^1^,^2^,^3^ Ultrasound utilization can be implemented in various ways in the prehospital setting. The extended-focused assessment with sonography in trauma (E-FAST) exam is a well-known standardized exam to evaluate for free fluid or air in the intrathoracic and intraabdominal cavity and identify the presence of large pneumothorax in trauma patients.^4^,^5^ The ultrasound exam is most contributory in the setting of traumatic injuries with associated hemodynamic instability or suspected pneumothoraces.^4^,^5^ The Advanced Trauma Life Support guidelines include E-FAST ultrasound scans as part of the primary survey to assess hypotensive trauma scenarios, adjuvant to assessing the airway patency, breathing adequacy, circulation, and disability.^6^ Prehospital ultrasound E-FAST exams have been shown to be more effective in detecting free fluid compared to relying on clinical exams alone.^7^ This efficiency may minimize the time taken for definitive surgical care and influence decision-making in prehospital trauma care.^7^ Point-of-care ultrasound has demonstrated its value in resource-limited rural settings by enabling the transmission of images to a central site, while also aiding in determining the appropriate patient destination and level of facility.^8^ Prior studies have been successful in teaching prehospital clinicians to use an ultrasound device and interpret ultrasound findings.^9^ In addition, EMS clinicians have shown interest in learning ultrasound in a hands-on training modality with oversight in real-life practice.^10^ Ultrasound education takes form in a variety of modalities contingent upon the institution, resources, and equipment availability. Moreover, both the availability and willingness of EMS learners and advanced sonographers significantly shape the educational approach.

### Problem identification, general and targeted needs assessment

Congruent with Kern’s 6-step curriculum development approach, curricula that have been effective in teaching FAST and E-FAST ultrasound have utilized a combination of both didactic and hands-on training in goal-specific agendas.^9^,^11^,^12^ FAST ultrasound curricula emphasized the importance of recognizing normal anatomy and distinguishing it from the presence of pathological free fluid on an exam.^9^ In addition, the majority of pleural ultrasound education employs sliding lung scale (SLS) as a teaching point and its absence as the primary indicator of a pneumothorax.^9^ The absence of SLS indicates the absence of movement between the visceral and parietal pleural surfaces during respiration, establishing it as the key determinant for identifying pneumothoraces, rather than relying on searching for comet tail artifacts.^5^,^9^ Moreover, conventional ultrasound curricula showed time-effective FAST and E-FAST education comprised of ultrasound theory, image acquisition, and image interpretation in six to eight hours of in-person teachings.^9^

Despite the proven effectiveness of ultrasound, its use in the prehospital setting remains infrequent in North America.^13^ Barriers, such as equipment and training cost and availability, hinder its integration into EMS systems.^13^, ^14^ However, advancements in portable ultrasound innovation and its decrease in costs have increased its accessibility to prehospital clinicians, potentially making ultrasound a more feasible investment for EMS systems.^13^, ^14^ Training EMS learners offers a unique opportunity to introduce ultrasound and provide fundamental education in anatomy, physiology, and pathophysiology. Potentially aligning with the needs of various early learners in emergency medicine, such as medical students, residents, and advanced practice clinicians, this emphasis underscores the necessity for open-access, cost-effective ultrasound education.

Online modules offer a high-yield alternative for self-paced learning, complemented by dedicated in-person scanning workshops.^9^ A hybrid approach would minimize lecture and space costs while maximizing focused hands-on scanning time. The flexibility of a hybrid curriculum also benefits prehospital clinicians with limited work schedules by providing accessible review content. Furthermore, it supports sonographer educators who have restricted time for didactic instruction. Future implications involve transitioning ultrasound education to clinical sites under the supervision of a trained sonographer. This allows prehospital clinicians to apply their skills to further diverse normal and pathologic images, enhancing their understanding of ultrasound’s role in clinical decision-making.

### Goals of the curriculum

Our proposed curriculum and study materials aim to provide a novel, open-access, asynchronous curriculum to educators with the desire to teach ultrasound fundamentals and E-FAST examinations to the skill set of their prehospital clinician colleagues.

### Objectives of the curriculum

By the end of these training activities, prehospital emergency medical service learners will be able to:

1. Demonstrate foundational ultrasound skills in E-FAST scanning, interpretation, and artifact recognition.
2. Acquire clinically adequate sonography images to identify pertinent organs and anatomically relevant structures for an E-FAST examination.
3. Differentiate between normal and pathologic E-FAST ultrasound images by accurately identifying the presence of free fluid in the views captured by E-FAST.
4. Identify the presence of a lung sliding sign in both Bright (B) and Motion (M)-Mode settings.
5. Explain the clinical significance and application of finding free fluid during an E-FAST scan.

### Educational strategies

Please see the curriculum chart below.

### Results and tips for successful implementation

Twenty-seven prehospital clinicians were initially registered and completed the pre-test for the asynchronous E-FAST ultrasound curriculum that took place in October 2022. This cohort included 12 emergency medical technician paramedics (EMT-P), six critical care EMT-Ps (CCEMTP), three critical care transport nurses (CCTN), and six certified flight registered nurses (CFRN). Following the pre-test, eight learners did not continue with the program and were not present for in-person ultrasound scanning (1 EMT-P, 3 CCEMTPs, 2 CCTNs, and 2 CFRNs) due to injury (1), lost to follow-up (4), and other work-related obligations (3). Participants who did not complete the study were excluded from the pre-test and post-test data analyses. Nineteen online post-tests were collected. The Wilcoxon signed-rank test was employed to compare the pre- and post-intervention scores with a significance-threshold alpha value of < 0.05, indicating a positive effect of the intervention.

The online multiple-choice pre-test had an average correct score of 7.21 (SD ± 2.04) out of 13 questions. The average pretest online confidence survey on a 1–10 Likert scale for using an ultrasound device was 2.63 (SD ± 1.74), understanding the purpose of the E-FAST exam was 6.37 (SD ± 2.97), obtaining the necessary views for an E-FAST exam was 3.68 (SD ± 2.11), identifying organs on an E-FAST exam was 4.16 (SD ± 1.80), and identifying pathology was 3.58 (SD ± 1.68).

The online multiple-choice post-test had an average correct score of 10.89 (SD ± 1.63) out of 13 questions (p < 0.0001). The average post-test online self-efficacy survey on a 1–10 Likert scale for using an ultrasound device was 6.47 (SD ± 1.78) (p < 0.0001), understanding the purpose of the E-FAST exam was 9.00 (SD ± 1.20) (p < 0.005), obtaining the necessary views for an E-FAST exam was 6.53 (SD ± 1.84) (p < 0.005), identifying organs on an E-FAST exam was 7.16 (SD ± 1.80) (p < 0.001), and identifying pathology was 7.16 (SD ± 2.01) (p < 0.0001).

A total of eighteen OSCE scans were proctored and graded. One participant was not able to attend a scheduled OSCE session due to work obligations. Out of 42 possible points, participants averaged a total score of 37.89 (SD ± 2.76) with the highest average scores attained in the right and left upper quadrant views (7.5 out of 8). The subxiphoid view had an average score of 7.17 out of 8, the anterior thoracic view was 7.06 out of 8, and the suprapubic view was 8.67 out of 10.

### Associated content

Appendix A: Pre-Screening Survey, Pre-Test and Answer Key

Appendix B: Introduction.mp4

Appendix C: Ultrasound Machine & Mechanics Part 1.mp4

Appendix D: Ultrasound Machine & Mechanics Part 1.pptx

Appendix E: Ultrasound Machine & Mechanics Part 2.mp4

Appendix F: Ultrasound Machine & Mechanics Part 2.pptx

Appendix G: Subxiphoid-Pericardial View.mp4

Appendix H: Subxiphoid-Pericardial View.pptx

Appendix I: Anterior Thoracic View.mp4

Appendix J: Anterior Thoracic View.pptx

Appendix K: Right Upper Quadrant View.mp4

Appendix L: Right Upper Quadrant View.pptx

Appendix M: Left Upper Quadrant View.mp4

Appendix N: Left Upper Quadrant View.pptx

Appendix O: Suprapubic View.mp4

Appendix P: Suprapubic View.pptx

Appendix Q: E-FAST Proctored Scanning Workshop Guide

Appendix R: Post-Screening Survey, Post-Test and Answer Key

Appendix S: OSCE Rubric

### Appendix YouTube Playlist

https://youtube.com/playlist?list=PL7LlW7XyJc_9pRYDvpD6ULzgHBEqwi67z&si=bsHe6D_C6IyOY0bp

### Evaluation and feedback

The introduction of ultrasound usage in the prehospital setting can be implemented in various ways, and our curriculum, materials, and tools can provide an effective groundwork in aiding this effort. This curriculum presents ultrasound foundations for prehospital clinicians with no experience with ultrasound interpretation, scanning techniques, or standardized scans such as the E-FAST exam. Learners were receptive to this curriculum and significantly improved their sonography knowledge, image interpretation, and confidence in ultrasound usage. Recorded lectures and associated PowerPoints provided pre-readings and initial exposure to the basic understanding of E-FAST significance and ultrasound usage. These materials were well received by our learners, and they have vocalized that it was beneficial in translating lecture concepts to their in-person scanning workshop. In doing so, learners were able to comprehend how to orient the probe, recognize artifacts, and identify normal organs and normal clinically relevant regions during the scanning of standardized patients or colleagues from the curriculum materials. In addition, learners were able to refer back to lecture resources after their in-person scanning to review pathologic findings and compare and contrast them to their normal findings during the workshop. This was shown to be effective in identifying normal and pathological findings in their pre-test and post-test data. Learners also reported that although the time between their in-person scanning workshop and in-person OSCEs felt significant, they were able to reorient themselves to scanning techniques during their OSCE evaluations.

Limitations to our curriculum include inconsistent ultrasound exposure. This institution currently does not require prehospital clinicians to use ultrasound routinely to fulfill their roles. The programs that took part in this study had the necessary equipment: portable ultrasound equipment, smart tablets, and gel for usage; however, the necessity to practice ultrasound outside of participation in this curriculum was limited. During the workshop, learners’ scanning practice was limited amongst a set of standardized patients and volunteering colleagues. In independent practice, available physician oversight was not routinely available. Physician oversight was largely variable and dependent on the institution’s organization and scheduling. Learners echoed that they would gain more comfort with ultrasound techniques with more frequent ultrasound use and having supportive physician oversight. Reflecting on our evaluation methods, we were limited in our data banks of pathologic ultrasound images to utilize for our pre and post-tests; therefore, a combination of both images and video clips was used to assess our learners. Finally, our OSCE rubric was categorized into meets expectations (ME) versus does not meet expectations (DNM) which may have been limiting in detailing how our learners could improve going forward.

Future curricula expanding upon this model may implement a longitudinal experience for prehospital clinicians in conjunction with physicians willing to support their continued ultrasound training experience. Future suggestions to improve the repetition and diversity of ultrasound scanning can be done by allowing prehospital clinicians to use ultrasound not only on standardized patients, but also amongst patients overseen by experienced sonographers such as physicians, residents, and advanced practitioners. This would enable prehospital clinicians to simulate bedside ultrasound practice, increase the frequency and diversity of ultrasound scans, and learn how an E-FAST scan may or may not influence clinical decision-making with providers. Finally, we encourage instructors to collect and present pathologic ultrasound images in addition to the materials provided by our curriculum for learners to appreciate a wider spectrum of pathologic findings. Our OSCE rubric may be improved with the addition of a “needs improvement” (NI) column with partial points and an area for facilitators to comment on how learners can improve for their next scan.

## Supplementary Information

















## Figures and Tables

**Table t2-jetem-9-1-c41:** 

Topic	Recommended Educational Strategy	Educational Content	Objectives	Learners	Timing, Resources Needed	Recommended Assessment, Milestones
Introduction	Hybrid, asynchronous curriculum allows learners to review educational content at their own pace. Content is ideally consumed consecutively, and within two weeks prior to hands-on scanning workshops. Dedicated in-person proctored ultrasound scanning session, following the completion of all videos within the virtual didactics portion.	Brief lecture introducing the significance and rising use of ultrasound in the prehospital setting. Introducing the significance of the E-FAST ultrasound exam.	Learners will become familiar with: The diverse applications of ultrasound in the prehospital setting, such as intrabdominal bleeds and pneumothoraces. The pros and cons of ultrasound use in real-world scenarios.	Prehospital Clinicians: EMR EMT EMT-P CCEMTP AEMT RN CCTN CFRN	15-minute video lecture Equipment: Computer 2-hour in-person proctored ultrasound scanning session with a brief didactic review: Instructors: 1 per 3–4 learners Equipment: 1 ultrasound machine and model per 3–4 learners	Milestone: PC9 Assessment: Real-time feedback by an instructor during hands-on proctored scanning workshops. Evaluation: Appendix A– Pre-Test multiple choice and Likert scale evaluation Appendix R – Post-Test multiple choice and Likert scale evaluation Appendix S – In-person post-test OSCE E-FAST Scanning Rubric
Ultrasound Mechanics	Hybrid, asynchronous curriculum allows learners to review educational content at their own pace. Content is ideally consumed consecutively, and within two weeks prior to hands-on scanning workshops. Dedicated in-person proctored ultrasound scanning session following the completion of all videos within the virtual didactics portion.	Overview of the physics background of ultrasounds. Introduction of knobology and the different settings of the ultrasound machine. Introduction of probe orientation, artifact recognition, and color doppler.	Learners will become familiar with: Terminology of probe positioning and manipulations: fanning, rotating/pivoting, heal/toe pressure. Terminology of interpreting ultrasound images (hyperechoic, hypoechoic, anechoic). Pertinent types of artifacts.	Prehospital Clinicians: EMR EMT EMT-P CCEMTP AEMT RN CCTN CFRN	25-minute video lecture (Part 1) Equipment: Computer 20-minute video lecture (Part 2) Equipment: Computer 2-hour in-person proctored ultrasound scanning session with a brief didactic review: Instructors: 1 per 3–4 learners Equipment: 1 ultrasound machine and model per 3–4 learners	Milestone: PC9 Assessment: Real-time feedback by an instructor during hands-on proctored scanning workshops. Evaluation: Appendix A – Pre-Test multiple choice and Likert scale evaluation Appendix R – Post-Test multiple choice and Likert scale evaluation Appendix S – In-person post-test OSCE E-FAST Scanning Rubric
Subxiphoid View	Hybrid, asynchronous curriculum allows learners to review educational content at their own pace. Content is ideally consumed consecutively, and within two weeks prior to hands-on scanning workshops. Dedicated in-person proctored ultrasound scanning session, following the completion of all videos within the virtual didactics portion.	Overview of how to capture the subxiphoid view in the E-FAST exam. Visualization of key landmarks, probe placement, and orientation. Review of pertinent anatomy. Presentation of normal versus abnormal E-FAST images demonstrated by the presence of a pericardial effusion.	The learner will demonstrate the ability to: Identify the xiphoid process and place the probe directly inferior. Place probe in transverse orientation with indicator towards patient’s right. Identify key anatomy: kidney, heart, and pericardial sac. Verbalizes how abnormal fluid accumulation in the pericardial sac would appear.	Prehospital Clinicians: EMR EMT EMT-P CCEMTP AEMT RN CCTN CFRN	15-minute video lecture Equipment: Computer 2-hour in-person proctored ultrasound scanning session with a brief didactic review: Instructors: 1 per 3–4 learners Equipment: 1 ultrasound machine and model per 3–4 learners	Milestone: PC9 Assessment: Real-time feedback by an instructor during hands-on proctored scanning workshops. Appendix Q – E-FAST Proctored Scanning Workshop Guide Evaluation: Appendix A – Pre-Test multiple choice and Likert scale evaluation Appendix R – Post-Test multiple choice and Likert scale evaluation Appendix S – In-person post-test OSCE E-FAST Scanning Rubric
Anterior Thoracic Lung View	Hybrid, asynchronous curriculum allows learners to review educational content at their own pace. Content is ideally consumed consecutively, and within two weeks prior to hands-on scanning workshops. Dedicated in-person proctored ultrasound scanning session, following the completion of all videos within the virtual didactics portion.	Overview of the anterior thoracic view of the E-FAST exam. Visualization of key anatomy and artifact recognition pertinent to identifying the lung sliding sign. Presentation of M-mode’s mechanics and interpretation used in identifying lung sliding. Reviewing the presence of a pneumothorax on an E-FAST exam.	The learner will demonstrate the ability to: Initially place the probe in the longitudinal axis at the midclavicular line around the third intercostal space. Identify key anatomy and artifact: ribs, rib shadowing, pleural line, A-lines, B-lines/comet tails. Verbalizes the presence and absence of lung sliding.	Prehospital Clinicians: EMR EMT EMT-P CCEMTP AEMT RN CCTN CFRN	20-minute video lecture Equipment: Computer 2-hour in-person proctored ultrasound scanning session with a brief didactic review: Instructors: 1 per 3–4 learners Equipment: 1 ultrasound machine and model per 3–4 learners	Milestone: PC9 Assessment: Real-time feedback by an instructor during hands-on proctored scanning workshops. Appendix Q – E-FAST Proctored Scanning Workshop Guide Evaluation: Appendix A – Pre-Test multiple choice and Likert scale evaluation Appendix R – Post-Test multiple choice and Likert scale evaluation Appendix S – In-person post-test OSCE E-FAST Scanning Rubric
Right Upper Quadrant View	Hybrid, asynchronous curriculum allows learners to review educational content at their own pace. Content is ideally consumed consecutively, and within two weeks prior to hands-on scanning workshops. Dedicated in-person proctored ultrasound scanning session, following the completion of all videos within the virtual didactics portion.	Overview of the Right Upper Quadrant view of the E-FAST exam. Review of key anatomy such as the liver and right kidney. Visualization of key landmarks, probe placement, and orientation. Presentation of normal versus abnormal ultrasound images of the hepatorenal recess (Morison’s pouch) by the presence of free fluid.	The learner will demonstrate the ability to: Correctly orient the probe in the longitudinal axis. Identify key anatomy: liver and right kidney. Verbalize the site of clinical interest, Morison’s pouch, and how free fluid would appear in ultrasound imaging.	Prehospital Clinicians: EMR EMT EMT-P CCEMTP AEMT RN CCTN CFRN	15-minute video lecture Equipment: Computer 2-hour in-person proctored ultrasound scanning session with a brief didactic review: Instructors: 1 per 3–4 learners Equipment: 1 ultrasound machine and model per 3–4 learners	Milestone: PC9 Assessment: Real-time feedback by an instructor during hands-on proctored scanning workshops. Appendix Q – E-FAST Proctored Scanning Workshop Guide Evaluation: Appendix A – Pre-Test multiple choice and Likert scale evaluation Appendix R – Post-Test multiple choice and Likert scale evaluation Appendix S – In-person post-test OSCE E-FAST Scanning Rubric
Left Upper Quadrant View	Hybrid, asynchronous curriculum allows learners to review educational content at their own pace. Content is ideally consumed consecutively, and within two weeks prior to hands-on scanning workshops. Dedicated in-person proctored ultrasound scanning session, following the completion of all videos within the virtual didactics portion.	Overview of the Left Upper Quadrant view of the E-FAST exam. Visualization of key landmarks, anatomy, probe placement, and orientation. Presentation of normal versus abnormal ultrasound images of the splenorenal recess by the presence of free fluid.	The learner will demonstrate the ability to: Correctly orient the probe in the longitudinal axis. Identify key anatomy: spleen and left kidney. Verbalize the site of clinical interest, splenorenal recess, and how free fluid would appear in ultrasound imaging.	Prehospital Clinicians: EMR EMT EMT-P CCEMTP AEMT RN CCTN CFRN	10-minute video lecture Equipment: Computer 2-hour in-person proctored ultrasound scanning session with a brief didactic review: Instructors: 1 per 3–4 learners Equipment: 1 ultrasound machine and model per 3–4 learners	Milestone: PC9 Assessment: Real-time feedback by an instructor during hands-on proctored scanning workshops. Appendix Q – E-FAST Proctored Scanning Workshop Guide Evaluation: Appendix A – Pre-Test multiple choice and Likert scale evaluation Appendix R – Post-Test multiple choice and Likert scale evaluation Appendix S – In-person post-test OSCE E-FAST Scanning Rubric
Suprapubic View	Hybrid, asynchronous curriculum allows learners to review educational content at their own pace. Content is ideally consumed consecutively, and within two weeks prior to hands-on scanning workshops. Dedicated in-person proctored ultrasound scanning session, following the completion of all videos within the virtual didactics portion.	Overview of the Suprapubic view of the E-FAST exam. Visualization of probe positioning and placement. Visualization of key anatomy in both male and female regions of interest. Presentation of normal versus abnormal ultrasound images of the bladder.	The learner will demonstrate the ability to: Scan the hypogastric-suprapubic region in both the transverse and longitudinal axis. Identify key anatomy: bladder, prostate/uterus. Verbalize the site of clinical interest, how abnormal fluid accumulation would appear around the bladder.	Prehospital Clinicians: EMR EMT EMT-P CCEMTP AEMT RN CCTN CFRN	10-minute video lecture Equipment: Computer 2-hour in-person proctored ultrasound scanning session with a brief didactic review: Instructors: 1 per 3–4 learners Equipment: 1 ultrasound machine and model per 3–4 learners	Milestone: PC9 Assessment: Real-time feedback by an instructor during hands-on proctored scanning workshops. Appendix Q – E-FAST Proctored Scanning Workshop Guide Evaluation: Appendix A – Pre-Test multiple choice and Likert scale evaluation Appendix R – Post-Test multiple choice and Likert scale evaluation Appendix S – In-person post-test OSCE E-FAST Scanning Rubric

## DIDACTICS AND HANDS-ON CURRICULUM

### Curriculum Chart

[Table t2-jetem-9-1-c41]

## Appendix A Pre-Screening Survey, Pre-Test and Answer Key

### Pre-Screening Survey

Please complete the survey below.

Thank you![Table t3-jetem-9-1-c41]

**Table t3-jetem-9-1-c41:**

	Please fill out the information below.	
1)	Work e-mail address	_______________________
2)	Current Date of Exam	_______________________
3)	Current Occupation	○ RN ○ Critical Care RN ○ Critical Care Transport RN ○ Flight RN ○ EMT - Basic ○ EMT - Intermediate ○ EMT - Advance ○ Paramedic ○ Flight Paramedic ○ Critical Care Paramedic ○ EMT/Paramedic ○ Physician ○ Other
4)	If you chose Other, please describe your role:	_______________________
5)	By checking this box, I certify that I am at least 18 years old, that I give my consent freely to participate in this study, and that I understand that by participating in this ultrasound research curriculum my involvement will not influence my standing at EMS Agency.	□ I consent

### Prehospital Ultrasound Exam Form

Please complete the survey below.

Thank you![Table t4-jetem-9-1-c41]

**Table t4-jetem-9-1-c41:**

	Prehospital Ultrasound Survey	
	Subxiphoid View (Author Owned)	
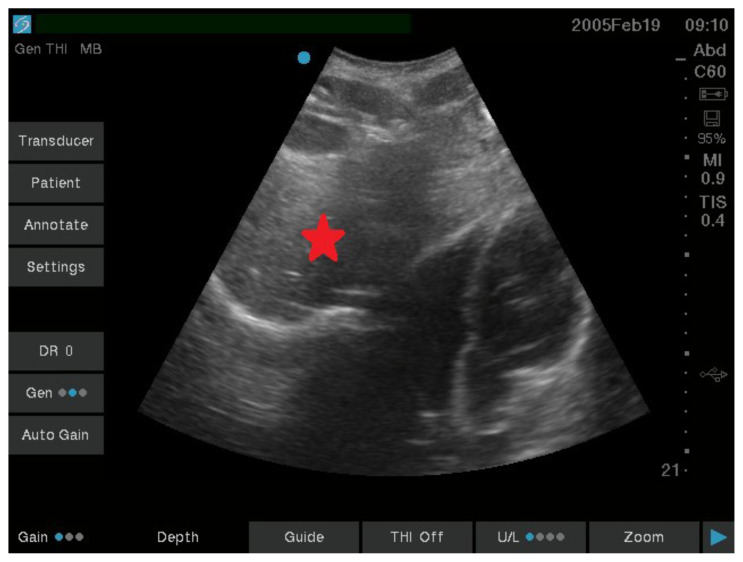
1)	In the subxiphoid view above, which organ is labeled with a red star?	○ Lung ○ Stomach ○ Liver ○ Kidney ○ Heart
	Subxiphoid (Author Owned)	
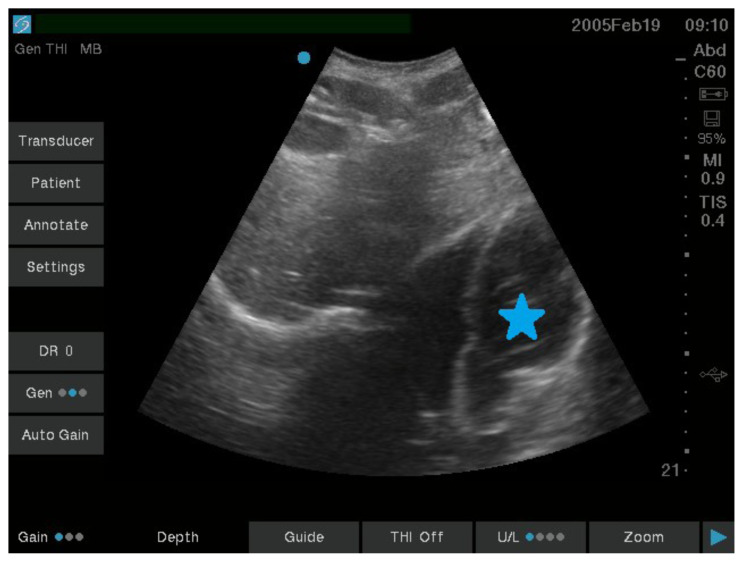
2)	In the subxiphoid view above, which organ is labeled with a blue star?	○ Lung ○ Stomach ○ Liver ○ Kidney ○ Heart
	Left Upper Quadrant (Author Owned)	
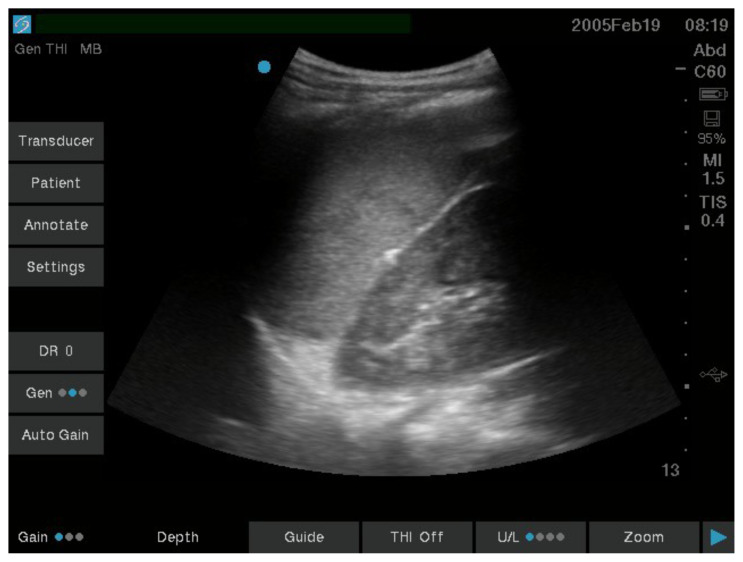
3)	In the left upper quadrant view above, which two organs are mainly shown?	○ Heart & Liver ○ Spleen & Kidney ○ Liver & Spleen ○ Liver & Kidney
	Right Upper Quadrant (Author Owned)	
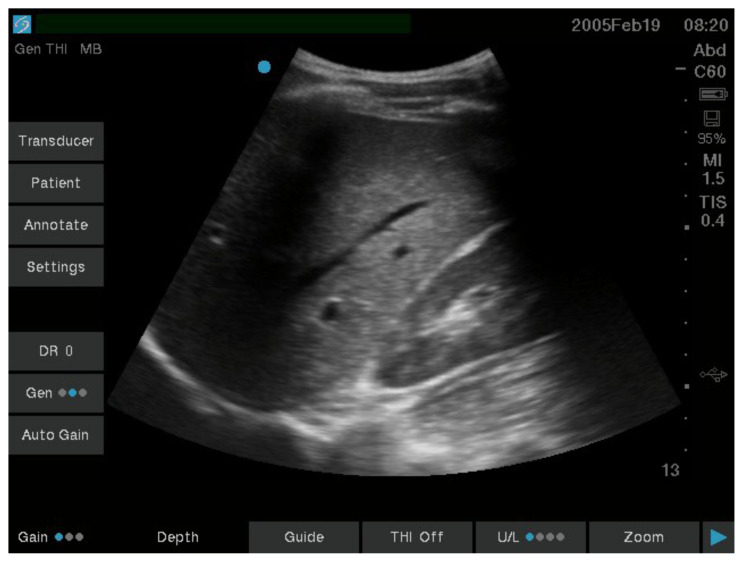
4)	In the right upper quadrant view above, which two organs are mainly shown?	○ Heart & Liver ○ Spleen & Kidney ○ Liver & Spleen ○ Liver & Kidney
5)	In the right upper quadrant view above, what is the potential space that free fluid can collect in?	○ Splenorenal recess ○ Hepatorenal recess; aka Morison’s Pouch ○ Rectovesical/Rectouterine recess; aka Pouch of Douglas ○ Pericardial cavity
	Anterior Thoracic View with M mode (Author Owned)	
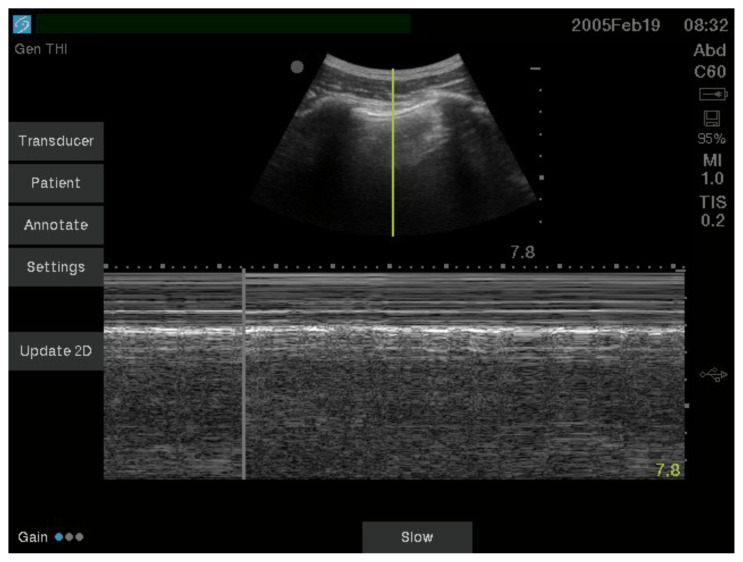
6)	Based on the M-mode capture above, is lung sliding present?	○ Yes, there is a Seashore Sign ○ No, there is a Barcode Sign ○ More information is needed to be determined
	Suprapubic View (Author Owned)	
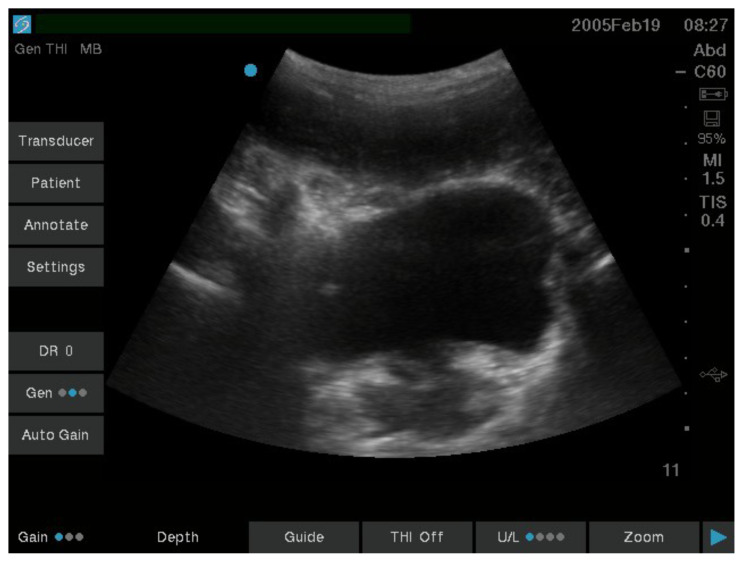
7)	How can a user describe the echogenicity of the contents of the bladder shown above?	○ Hyperechoic ○ Hypoechoic ○ Anechoic
	Suprapubic View (Author Owned)	
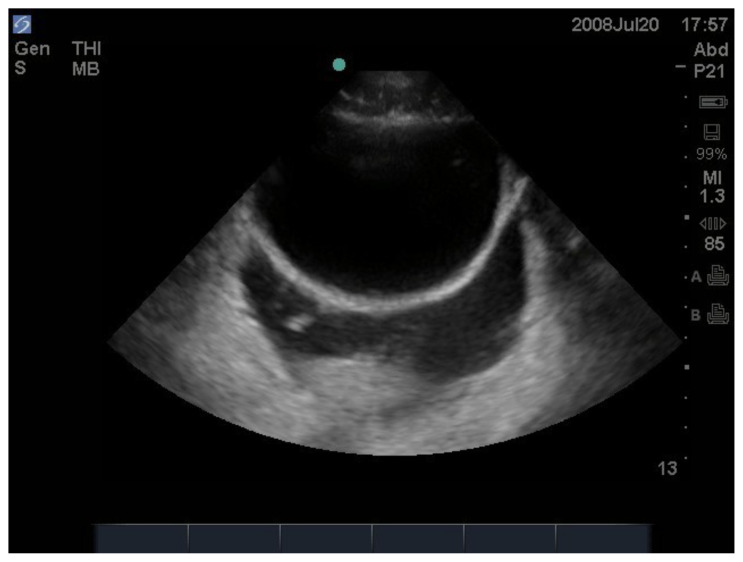
8)	In the suprapubic view shown above, is there free fluid present?	○ Yes, the anechoic content in the Pouch of Douglas ○ Yes, the hyperechoic content in Morison’s Pouch ○ No, there is no free fluid ○ More information is needed to be determined
	Subxiphoid/Pericardial View (Author Owned)	
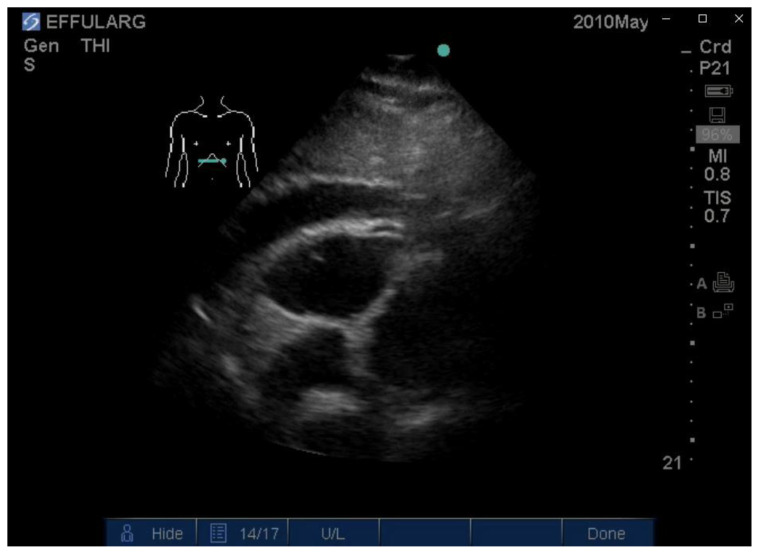
9)	In the subxiphoid view shown above, is there free fluid present?	○ Yes, the anechoic content within Morison’s Pouch ○ Yes, the anechoic content in the pericardial sac ○ No, there is no free fluid ○ More information is needed to be determined
	Right Upper Quadrant View (Author Owned)	
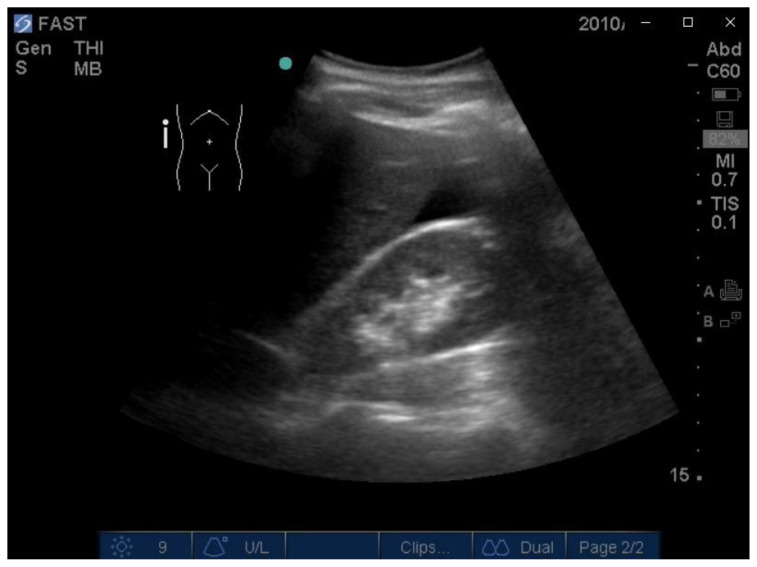
10)	In the right upper quadrant view shown above, is there free fluid present?	○ Yes, the anechoic content within Morison’s Pouch ○ Yes, the hyperechoic content within Morison’s Pouch ○ No, there is no free fluid ○ More information is needed to be determined
	Anterior Thoracic View (Author Owned)	
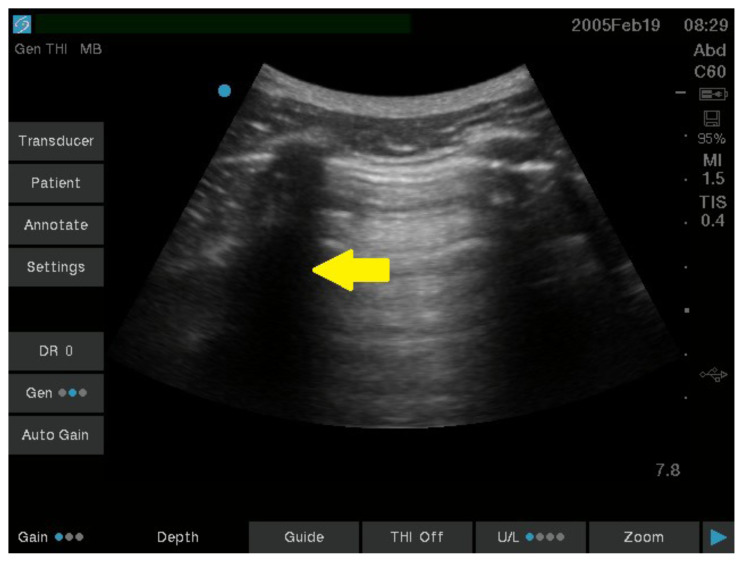
11)	In the anterior thoracic (lung) view shown above, what is the yellow arrow pointing to underneath the ribs?	○ A-Line artifact ○ Pleural Line ○ Rib Shadowing Artifact ○ Sliding Lung Sign ○ Free Fluid
	Anterior Thoracic View (Author Owned)	
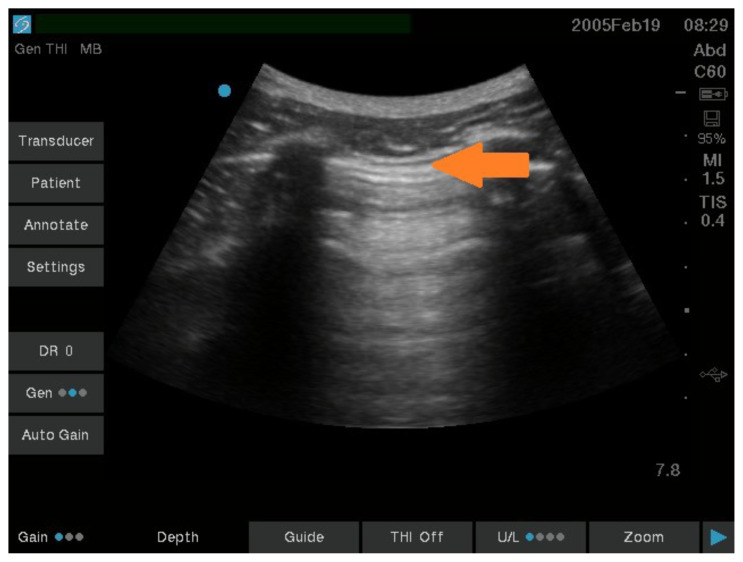
12)	In the lung view shown above, what is the Orange arrow pointing to?	○ Ribs ○ Pleural Line ○ Diaphragm ○ Heart
	Right Upper Quadrant (Author Owned)	
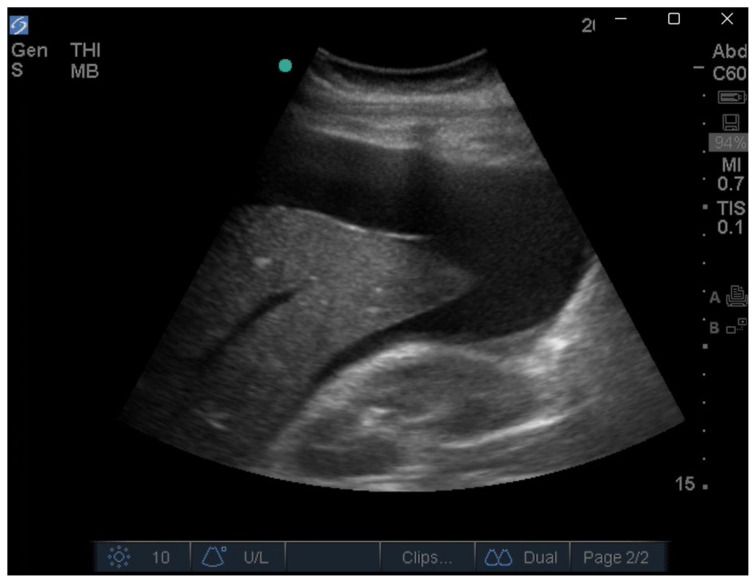
13)	A patient presents with altered mental status and appears diaphoretic. Blood pressure of 60/40. On a succinct eFAST exam, you find the following image above on the Right Upper Quadrant view. What disposition is best for the patient for definitive treatment?	○ Observation with adequate hydration by Normal Saline IV ○ Transport to the nearest Urgent Care clinic for further evaluation ○ Transport to the nearest Emergency Department for monitored blood transfusion IV ○ Transport to the nearest Trauma Surgical Center for operative management

### Learner’s Confidence Survey

Please complete the survey below.

Thank you![Table t5-jetem-9-1-c41]

**Table t5-jetem-9-1-c41:**

	As a participant in this study, please answer the questions below on a scale on a scale of 1 to 10. (1 = Not confident at all, 10 = Very confident)	
	E-FAST Ultrasound Exam Score (out of 13 questions)	_______________________
1)	How confident do you feel about using an ultrasound device?	○ 1 ○ 2 ○ 3 ○ 4 ○ 5 ○ 6 ○ 7 ○ 8 ○ 9 ○ 10
2)	How confident do you feel about understanding the purpose of the EFAST exam?	○ 1 ○ 2 ○ 3 ○ 4 ○ 5 ○ 6 ○ 7 ○ 8 ○ 9 ○ 10
3)	How confident do you feel about obtaining the views necessary for an eFAST?	○ 1 ○ 2 ○ 3 ○ 4 ○ 5 ○ 6 ○ 7 ○ 8 ○ 9 ○ 10
4)	How confident do you feel about identifying organs on an eFAST exam?	○ 1 ○ 2 ○ 3 ○ 4 ○ 5 ○ 6 ○ 7 ○ 8 ○ 9 ○ 10
5)	How confident do you feel about identifying pathologic free fluid on an eFAST exam?	○ 1 ○ 2 ○ 3 ○ 4 ○ 5 ○ 6 ○ 7 ○ 8 ○ 9 ○ 10

### Answer Key

1. Liver
2. Heart
3. Spleen & Kidney
4. Liver & Kidney
5. Hepatorenal recess; aka Morison’s Pouch
6. Yes, there is a Seashore Sign
7. Anechoic
8. Yes, the anechoic content in the Pouch of Douglas
9. Yes, the anechoic content in the pericardial sac
10. Yes, the anechoic content within Morison’s Pouch
11. Rib Shadowing Artifact
12. Pleural Line
13. Transport to the nearest Trauma Surgical Center for operative management

## Appendix B Introduction

Please see associated Power Point Lecture Link: https://youtu.be/HEAHCCP8EI8

## Appendix C & D Ultrasound Machine & Mechanics Part 1

Please see associated Power Point Lecture Link: https://youtu.be/yvn98wL8UYE

## Appendix E & F Ultrasound Machine & Mechanics Part 2

Please see associated Power Point Lecture Link: https://youtu.be/oc2v2DbcZyg

## Appendix G & H Subxiphoid-Pericardial View

Please see associated Power Point Lecture Link: https://youtu.be/yE5otm5elWw

## Appendix I & J Anterior Thoracic View

Please see associated Power Point Lecture Link: https://youtu.be/ibPAPW4TEfw

## Appendix K & L Right Upper Quadrant View

Please see associated Power Point Lecture Link: https://youtu.be/M57N0Gx4sWQ

## Appendix M & N Left Upper Quadrant View

Please see associated Power Point Lecture Link: https://youtu.be/7hZiNLUffAs

## Appendix O & P Suprapubic View

Please see associated Power Point Lecture Link: https://youtu.be/rVHFzFKGVV0

## Appendix Q E-FAST Proctored Scanning Workshop Guide

**Ultrasound Basics**

- Demonstrate adjusting gain, depth, and focus to optimize the ultrasound image.
- Identify deep and superficial structures on ultrasound images.
- Differentiate fluid, bone, and variable soft tissues on ultrasound images.
- Compare and contrast the different types of transducers and their applications.
- Relate the concept of indicator to anatomy and probe orientation.
- Understand the different ultrasound modes and their applications (B-mode, M-mode, Doppler mode).
- Demonstrate knowledge and understanding of basic physics and ‘knobology’ while scanning to position the probe and patient appropriately for viewing desired structures.

**E-FAST Exam**: to detect free fluid surrounding the abdominal organs in the case of trauma. Fluid appears black (anechoic) on the ultrasound images.

You should use a low frequency transducer (greater depth, lower resolution) for the following four views:

- **Subxiphoid (pericardial) view**
  - Location: directly inferior to the xiphoid process
  - Orientation of transducer: transverse, indicator towards patient’s right
  - Approximate depth: 21cm
  - Important structures: heart, pericardial sac
  - Looking for: pericardial free fluid
- **Right upper quadrant view**
  - Location: right mid axillary line, around the 10–11th intercostal space
  - Orientation of transducer: longitudinal, indicator towards patient’s head
  - Approximate depth: 16cm
  - Important structures: right kidney, liver, diaphragm
  - Looking for: fluid in Morrison’s pouch
- **Left upper quadrant view**
  - Location: left mid to posterior axillary lines, around the 8–9th intercostal space
  - Orientation of transducer: longitudinal, indicator towards patient’s head
  - Approximate depth: 16cm
  - Important structures: left kidney, spleen, diaphragm
  - Looking for: fluid in the splenorenal interface
- **Suprapubic view**
  - Location: superior to the pubic bone
  - Orientation of transducer: transverse (indicator towards pt’s right) and longitudinal (indicator towards pt’s head)
  - Approximate depth: 16cm
  - Important structures: bladder
  - Looking for: free fluid around the bladder

You should use a higher frequency transducer (less depth, higher resolution) for the following view:

- **Anterior thoracic lung view**
  - Location: along the midclavicular line at the level of the third and fourth intercostal spaces (scan BOTH left and right!)
  - Orientation of transducer: longitudinal (indicator towards pt’s head)
  - Approximate depth: 5cm
  - Important structures: rib shadow, pleural line
  - M-mode:
    ▪ Normal exam: sliding lung sign (waves on the beach)
    ▪ Abnormal exam (pneumothorax): barcode sign

## Appendix R Post-Screening Survey, Post-Test and Answer Key

Link to Post-Test Question #1 Subxiphoid View (Author Owned):

https://youtu.be/sjOBPxM_NmU

Link to Post-Test Question #2–4 Right Upper Quadrant View (Author Owned):

https://youtu.be/nr7FdyTj23Y

Link to Post-Test Question #5 Subxiphoid View (Author Owned):

https://youtu.be/ZfCES0RjxGg

Link to Post-Test Question #6 Subxiphoid View (Author Owned):

https://youtu.be/aKfWj1tD8Rs

Link to Post-Test Question #9 Suprapubic View (Author Owned):

https://youtu.be/2qQjMhdkmjY

Link to Post-Test Question #10 Lung View (Author Owned):

https://youtu.be/PNlE_I_uojU

Link to Post-Test Question #12 Lung View (Author Owned):

https://youtu.be/NXOgEzB9j0g

Link to Post-Test Question #13 Right Upper Quadrant View (Author Owned):

https://youtu.be/zMVLLRbTB0w

### Post-Screening Survey

Please complete the survey below.

Thank you![Table t6-jetem-9-1-c41]

**Table t6-jetem-9-1-c41:**

	Please fill out the information below.	
1)	Work e-mail address	_______________________
2)	Current Date of Exam	_______________________
3)	Current Occupation	○ RN ○ Critical Care RN ○ Critical Care Transport RN ○ Flight RN ○ EMT - Basic ○ EMT - Intermediate ○ EMT - Advanced ○ Paramedic ○ Flight Paramedic ○ Critical Care Paramedic ○ EMT/Paramedic ○ Physician ○ Other
4)	If you chose Other, please describe your role:	_______________________
5)	By checking this box, I certify that I am at least 18 years old, that I give my consent freely to participate in this study, and that I understand that by participating in this ultrasound research curriculum my involvement will not influence my standing at EMS Agency.	□ I consent

### Prehospital Ultrasound Exam Form

Please complete the survey below.

Thank you![Table t7-jetem-9-1-c41]

**Table t7-jetem-9-1-c41:**

	Prehospital Ultrasound Survey	
	Question 1: Subxiphoid View (Author Owned)	
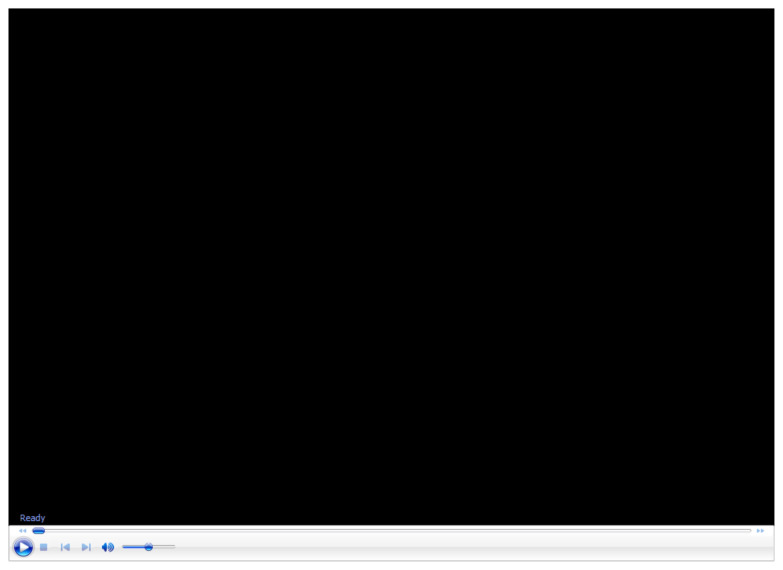
1)	Q1: In the subxiphoid view shown above, is there free fluid present?	○ Yes, positive E-FAST (+) free fluid in Morison’s Pouch. ○ Yes, positive E-FAST (+) free fluid in the pericardial sac. ○ No, negative E-FAST (−) no free fluid in splenorenal recess. ○ More information is needed to be determined.
	Questions 2–4: Right Upper Quadrant (Author Owned)	
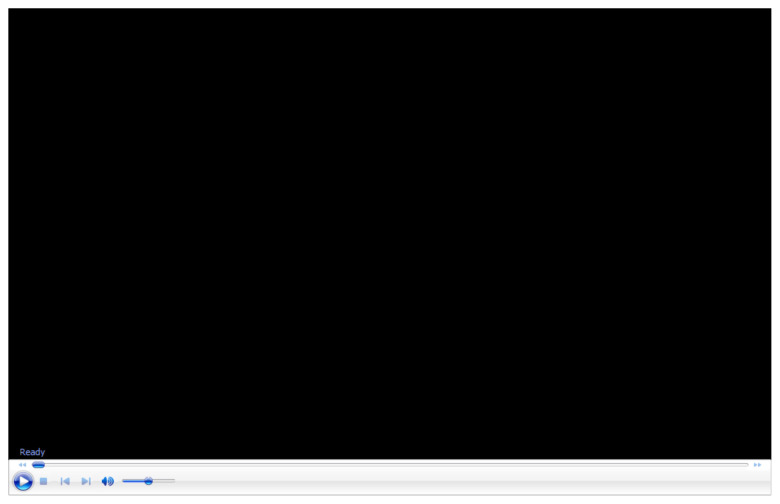
2)	Q2: In the Right Upper Quadrant view above, which Stomach organ is labeled with a “blue question mark” ?	○ Lung ○ Kidney ○ Liver ○ Heart ○ Bladder
3)	Q3: In the Right Upper Quadrant view above, which organ is identified with the “yellow X” ?	○ Lung ○ Spleen ○ Heart ○ Liver ○ Kidney
4)	Q4: In the Right Upper Quadrant view above, is there free fluid present?	○ Yes, positive E-FAST (+) free fluid in Morison’s Pouch. ○ Yes, positive E-FAST (+) free fluid below the bladder. ○ No, negative E-FAST (−) no free fluid in Morison’s Pouch. ○ No, negative E-FAST (−) no free fluid around the Heart.
	Question 5: Subxiphoid View (Author Owned)	
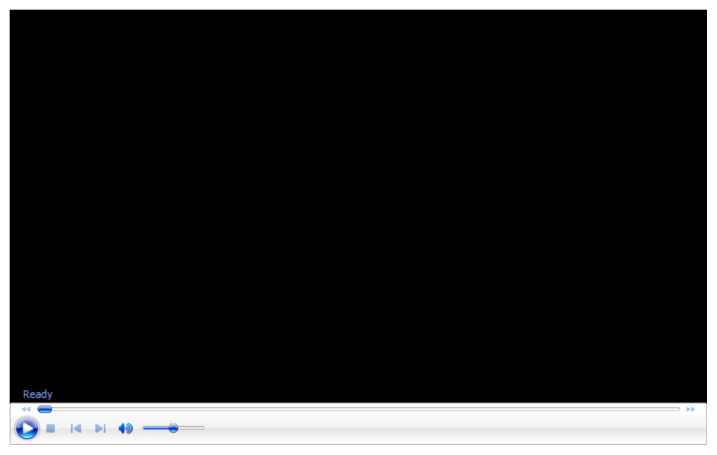
5)	Q5: In the subxiphoid view shown above, what organ is identified by the Orange Arrow?	○ Heart ○ Liver ○ Spleen ○ Kidney ○ Lung
	Question 6: Subxiphoid View (Author Owned)	
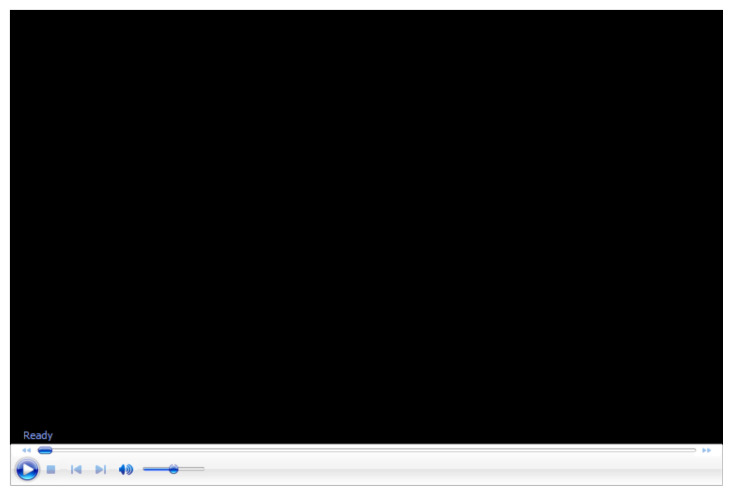
6)	Q6: In the subxiphoid view above, what organ is identified by the Yellow Stars?	○ Bladder ○ Liver ○ Heart ○ Kidney ○ Lung
	Question 7–8: Left Upper Quadrant (Author Owned)	
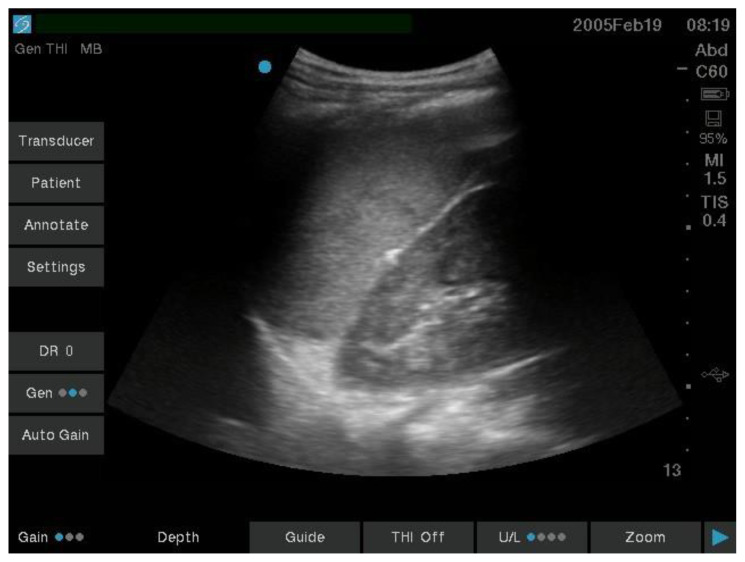
7)	Q7: In the Left Upper Quadrant View shown above, what potential space can free fluid collect in?	○ Splenorenal recess ○ Hepatorenal recess; aka Morison’s Pouch ○ Rectovesical/Rectouterine recess; aka Pouch of Douglas ○ Pericardial cavity
8)	Q8: In the Left Upper Quadrant view shown above, is there free fluid present?	○ Yes, positive E-FAST (+) free fluid in Morison’s Pouch. ○ Yes, positive E-FAST (+) free fluid in the pericardial sac. ○ No, negative E-FAST (−) no free fluid in splenorenal recess. ○ No, negative E-FAST (−) no free fluid in the Pouch of Douglas.
	Question 9: Suprapubic View (Author Owned)	
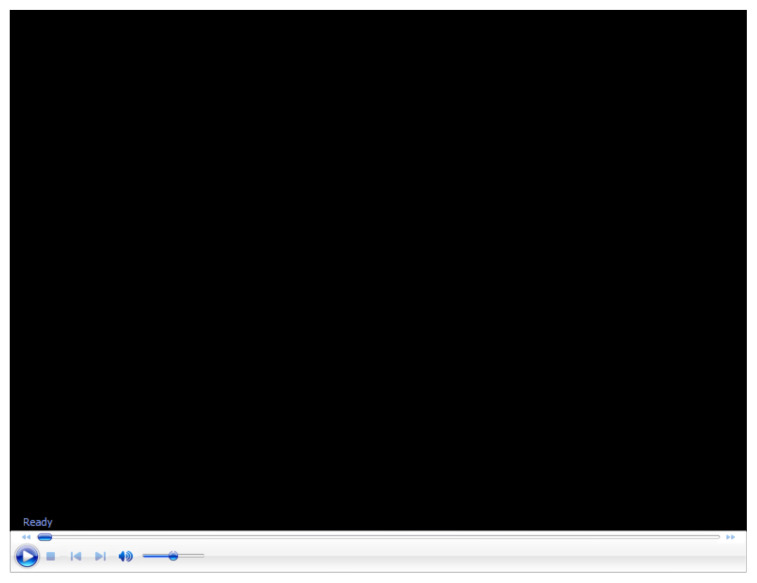
9)	Q9: In the Suprapubic View shown above, is there free fluid present?	○ Yes, positive E-FAST (+) free fluid in the pericardial sac. ○ Yes, positive E-FAST (+) free fluid in the rectovesical pouch. ○ No, negative E-FAST (−) no free fluid in the rectovesical pouch. ○ More information is needed to be determined.
	Question 10: Anterior Thoracic Window (Author Owned)	
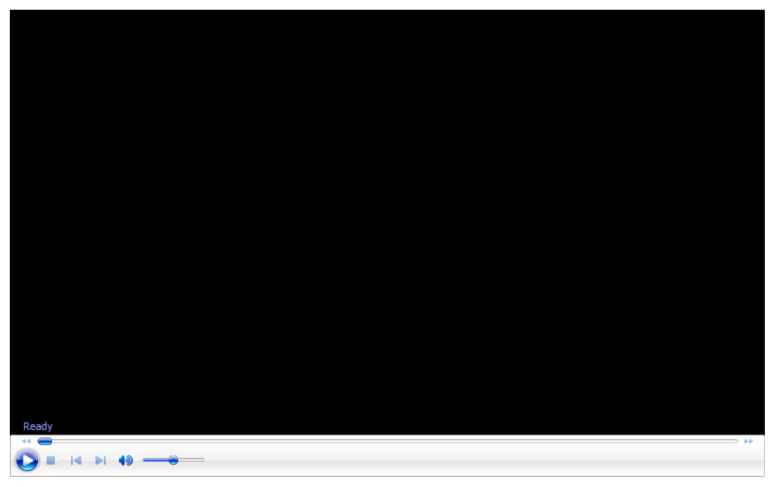
	Question 10: Anterior Thoracic View with M mode (Author Owned)	
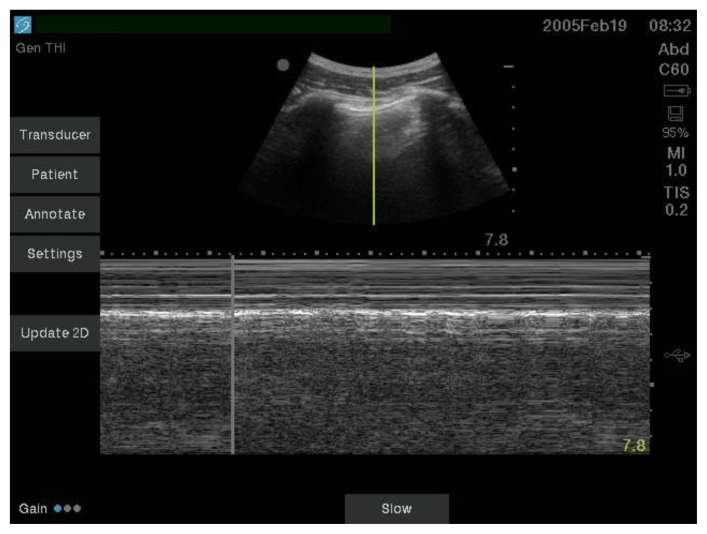
10)	Q10: From the 2 Lung Windows shown above (Video & M-Mode image), is there lung sliding present?	○ Yes, lung sliding present with sliding B-lines and Seashore Sign. ○ No, absent lung sliding with absent B-line sliding. ○ No, absent lung sliding with Barcode Sign. ○ More information is needed to be determined
	Question 11: Anterior Thoracic View (Author Owned)	
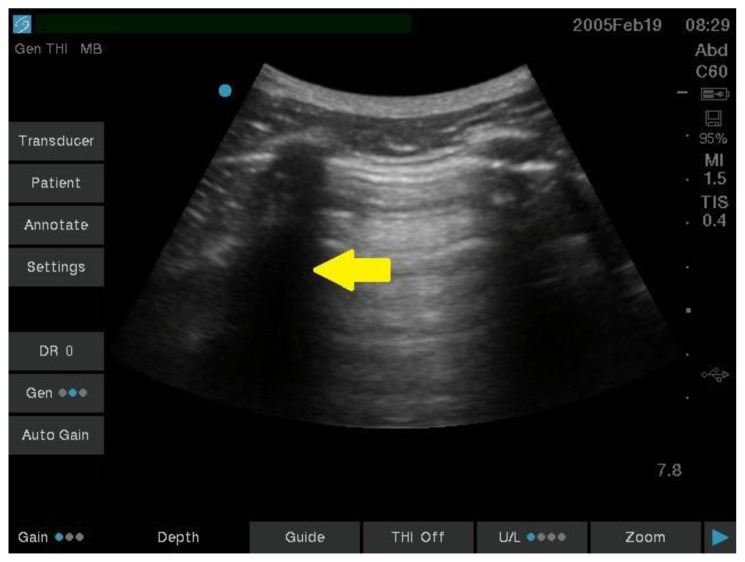
11)	Q11: In the anterior thoracic (lung) view shown above, what is the yellow arrow pointing to underneath the ribs?	○ Free Fluid ○ A-Line artifact ○ Sliding Lung Sign ○ Rib Shadowing Artifact ○ Pleural Line
	Question 12: Anterior Thoracic View (Author Owned)	
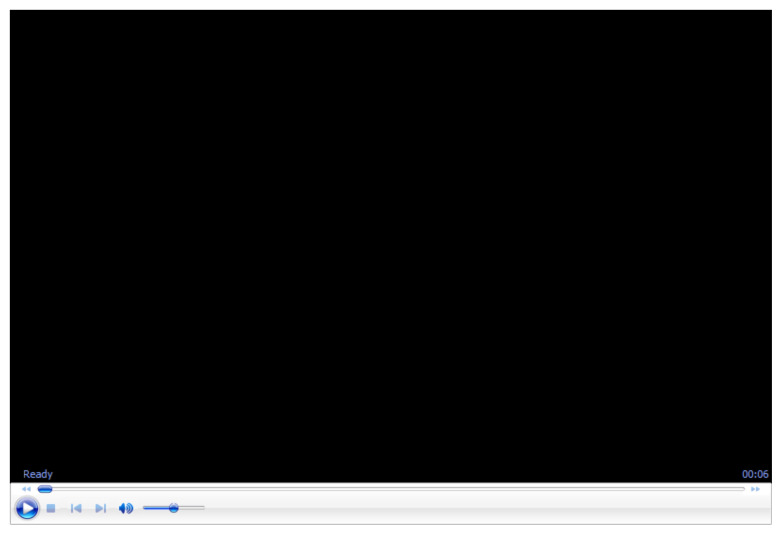
	Question 12: Anterior Thoracic View with M mode (Author Owned)	
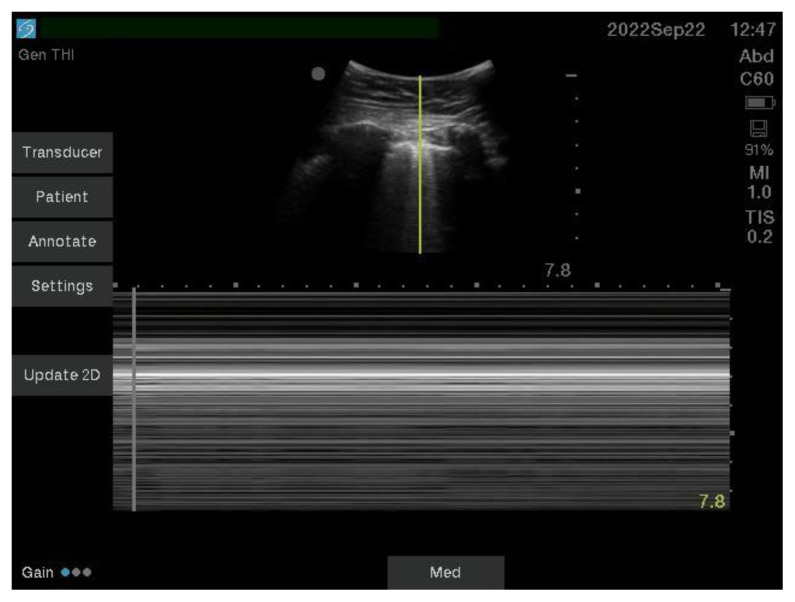
12)	Q12: From the 2 Lung Windows shown above (Video & M-Mode image), is there lung sliding present?	○ Yes, lung sliding present with sliding B-lines. ○ No, absent lung sliding with Seashore Sign. ○ No, absent lung sliding with absent B-line sliding and Barcode Sign. ○ More information is needed to be determined
	Question 13: Right Upper Quadrant (Author Owned)	
13)	Q13: A patient presents with altered mental status and appears diaphoretic. Blood pressure of 60/40. On a succinct eFAST exam, you find the following image above on the Right Upper Quadrant view. What disposition is best for the patient for definitive treatment?	○ Observation with adequate hydration by Normal Saline IV ○ Transport to the nearest Urgent Care clinic for further evaluation ○ Transport to the nearest Emergency Department for monitored blood transfusion IV. ○ Transport to the nearest Trauma Surgical Center for operative management.

### Learner’s Confidence Survey

Please complete the survey below.

Thank you![Table t8-jetem-9-1-c41]

**Table t8-jetem-9-1-c41:**

	As a participant in this study, please answer the questions below on a scale on a scale of 1 to 10. (1 = Not confident at all, 10 = Very confident)	
	E-FAST Ultrasound Exam Score (out of 13 questions)	_______________________
1)	How confident do you feel about using an ultrasound device?	○ 1 ○ 2 ○ 3 ○ 4 ○ 5 ○ 6 ○ 7 ○ 8 ○ 9 ○ 10
2)	How confident do you feel about understanding the purpose of the EFAST exam?	○ 1 ○ 2 ○ 3 ○ 4 ○ 5 ○ 6 ○ 7 ○ 8 ○ 9 ○ 10
3)	How confident do you feel about obtaining the views necessary for an eFAST?	○ 1 ○ 2 ○ 3 ○ 4 ○ 5 ○ 6 ○ 7 ○ 8 ○ 9 ○ 10
4)	How confident do you feel about identifying organs on an eFAST exam?	○ 1 ○ 2 ○ 3 ○ 4 ○ 5 ○ 6 ○ 7 ○ 8 ○ 9 ○ 10
5)	How confident do you feel about identifying pathologic free fluid on an eFAST exam?	○ 1 ○ 2 ○ 3 ○ 4 ○ 5 ○ 6 ○ 7 ○ 8 ○ 9 ○ 10

### Answer Key

1. Yes, positive E-FAST (+) free fluid in the pericardial sac.
2. Liver
3. Kidney
4. Yes, positive E-FAST (+) free fluid in Morison’s Pouch.
5. Liver
6. Heart
7. Splenorenal recess
8. No, negative E-FAST (−) no free fluid in splenorenal recess.
9. Yes, positive E-FAST (+) free fluid in the rectovesical pouch.
10. Yes, lung sliding present with sliding B-lines and Seashore Sign.
11. Rib Shadowing Artifact
12. No, absent lung sliding with absent B-line sliding and Barcode Sign.
13. Transport to the nearest Trauma Surgical Center for operative management

## Appendix S OSCE Rubric

**Prehospital Extended Focused Assessment with Sonography for Trauma (E-FAST) Objective Structured Clinical Examination**

Learner:______________________ Date:_____________

Evaluator:_____________________

ME = Meets Expectations (Student Completed Task Correctly)

DNM = Does Not Meet Expectations (Task Not Performed or Performed Incorrectly)[Table t9-jetem-9-1-c41]

**Table t9-jetem-9-1-c41:**

	ME (1 pt)	DNM (0 pts)
**Right upper quadrant view**		
Initially places the probe at the right mid-axillary line around the 10–11^th^ intercostal space.		
Places probe in a longitudinal orientation with the indicator towards the patient’s head.		
Manipulates probe to appropriate plane of view.		
Acquired appropriate window for the patient.		
Adjusts depth and gain appropriately.		
Able to identify key anatomy: right kidney, liver, and diaphragm.		
Verbalized site of clinical interest and how fluid accumulation in Morison’s pouch would appear.		
Fans through structures to assess all potential areas for fluid accumulation.		
**RUQ Total:____/8**		
**Subxiphoid (pericardial) view**		
Identifies xiphoid process and places probe directly inferior.		
Places probe in transverse orientation with indicator towards patient’s right.		
Manipulates probe to appropriate plane of view.		
Acquired appropriate window for the patient.		
Adjusts depth and gain appropriately.		
Identifies key anatomy: kidney, heart, and pericardial sac.		
Verbalizes how abnormal fluid accumulation in the pericardial sac would appear.		
Fans through structure to assess all potential areas for fluid accumulation.		
**Subxiphoid Total:____/8**		
**Left upper quadrant view**		
Initially places the probe at the left mid to posterior axillary line around the 8–9^th^ intercostal space.		
Places probe in longitudinal orientation with the indicator towards the patient’s head.		
Manipulates probe to appropriate plane of view.		
Acquired appropriate window for the patient.		
Adjusts depth and gain appropriately.		
Identifies key anatomy: left kidney, spleen, and diaphragm.		
Verbalizes how abnormal fluid accumulation would appear in the splenorenal interface.		
Fans through structures to assess all potential areas for fluid accumulation.		
**LUQ Total:____/8**		
**Suprapubic view**		
Initially places the probe at the hypogastric-suprapubic region.		
Places probe in transverse orientation with the indicator towards the patient’s right.		
Manipulates probe to appropriate plane of view.		
Acquired appropriate window for the patient.		
Adjusts depth and gain appropriately.		
Identifies key anatomy: bladder and prostate/uterus.		
Verbalizes how abnormal fluid accumulation would appear around the bladder.		
Fans through structures to assess all potential areas for fluid accumulation.		
Rotates probe to longitudinal orientation with indicator towards the patient’s head.		
Fans through structures again to assess all potential areas for fluid accumulation.		
**Suprapubic Total:____/10**		
**Anterior thoracic view**		
Initially places the probe at the midclavicular line around the third intercostal space.		
Places probe in longitudinal orientation with the indicator toward the patient’s head.		
Adjusts depth and gain appropriately.		
Identifies key anatomy and artifact: ribs, rib shadowing, pleural line, A-lines, B-lines/comet tails.		
Verbalizes the presence and absence of lung sliding.		
Demonstrates how to use M-mode.		
Verbalizes the presence and absence of lung sliding on M-mode.		
Scans both left and right.		
**Anterior Thoracic Total:____/8**		
**Total Score:____/42**		
