# Supplementary material for: E-FAST Ultrasound Training Curriculum for Prehospital Emergency Medical Service (EMS) Clinicians
Source: J Educ Teach Emerg Med. 2024 Jan 31;9(1):C41–97. doi: 10.21980/J8S060 (PMC10854885; doi:10.21980/J8S060)
Supplement: Supplementary file 11 — Please see associated Power Point Lecture Link: https://youtu.be/yvn98wL8UYE [file jetem-9-1-C41-AppendixD.pptx]

## Slide 1
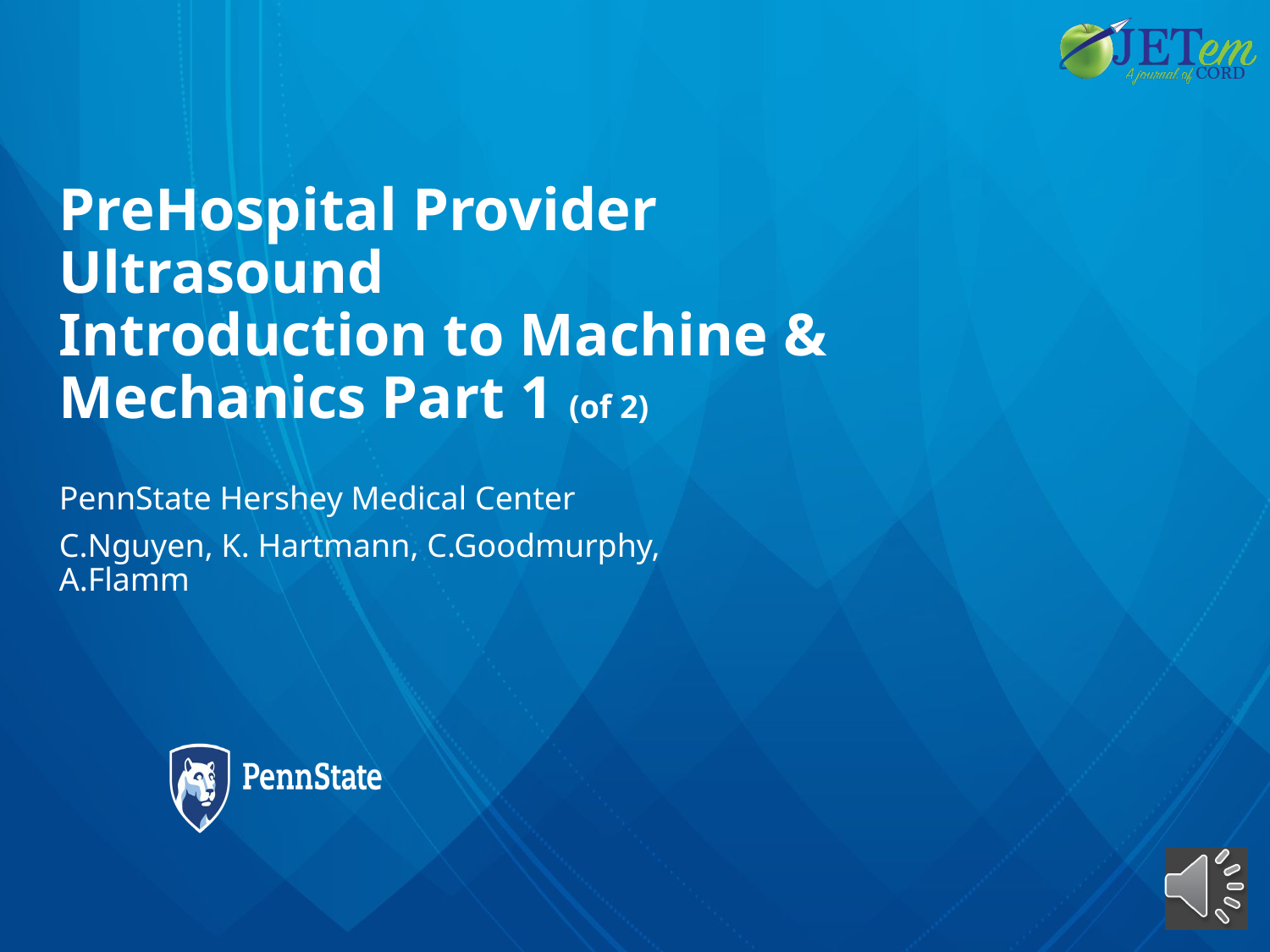

# PreHospital Provider Ultrasound Introduction to Machine & Mechanics Part 1 (of 2)
PennState Hershey Medical Center
C.Nguyen, K. Hartmann, C.Goodmurphy, A.Flamm

## Slide 2
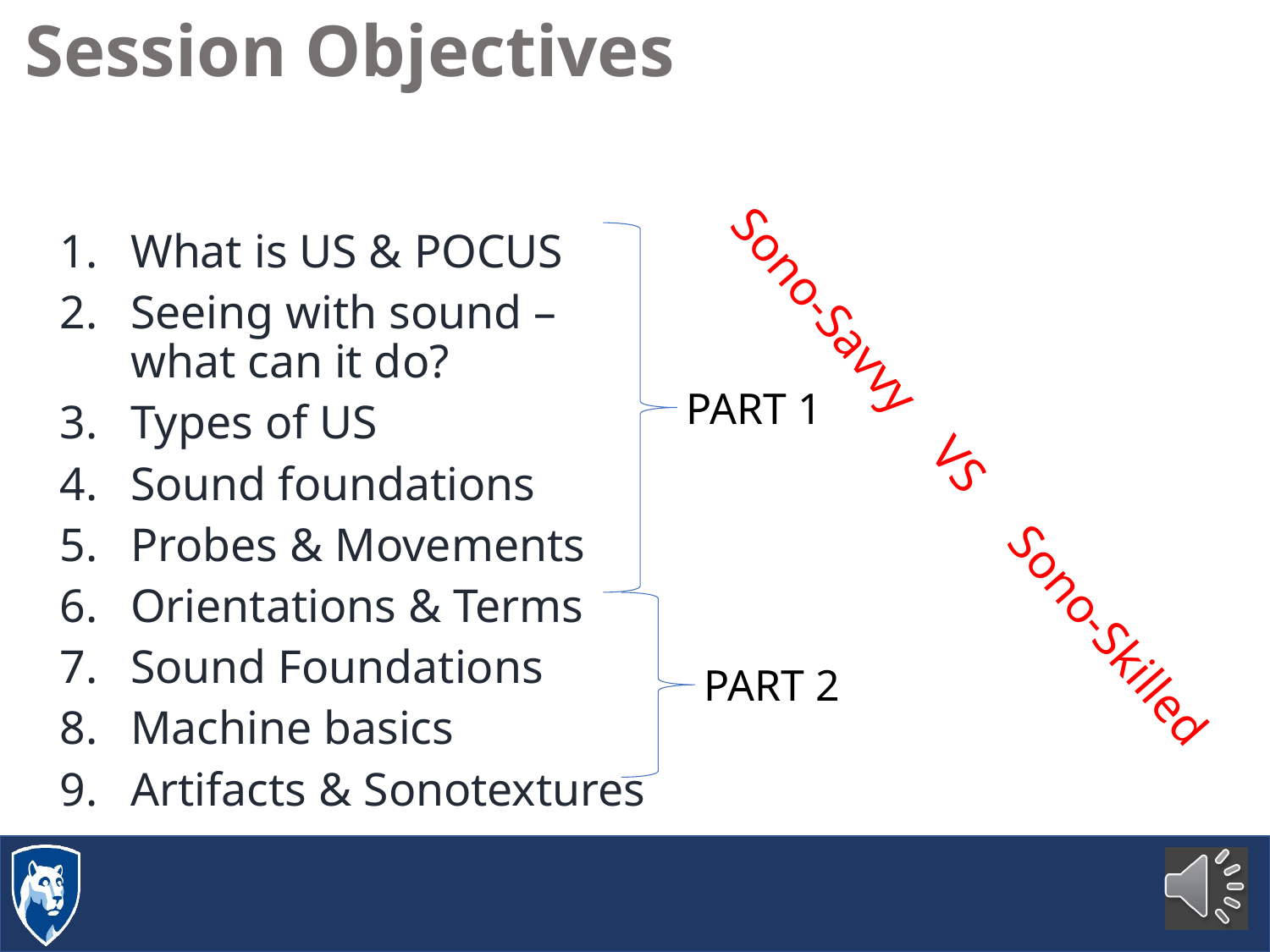

# Session Objectives
What is US & POCUS
Seeing with sound – what can it do?
Types of US
Sound foundations
Probes & Movements
Orientations & Terms
Sound Foundations
Machine basics
Artifacts & Sonotextures
PART 1
Sono-Savvy VS Sono-Skilled
PART 2

## Slide 3
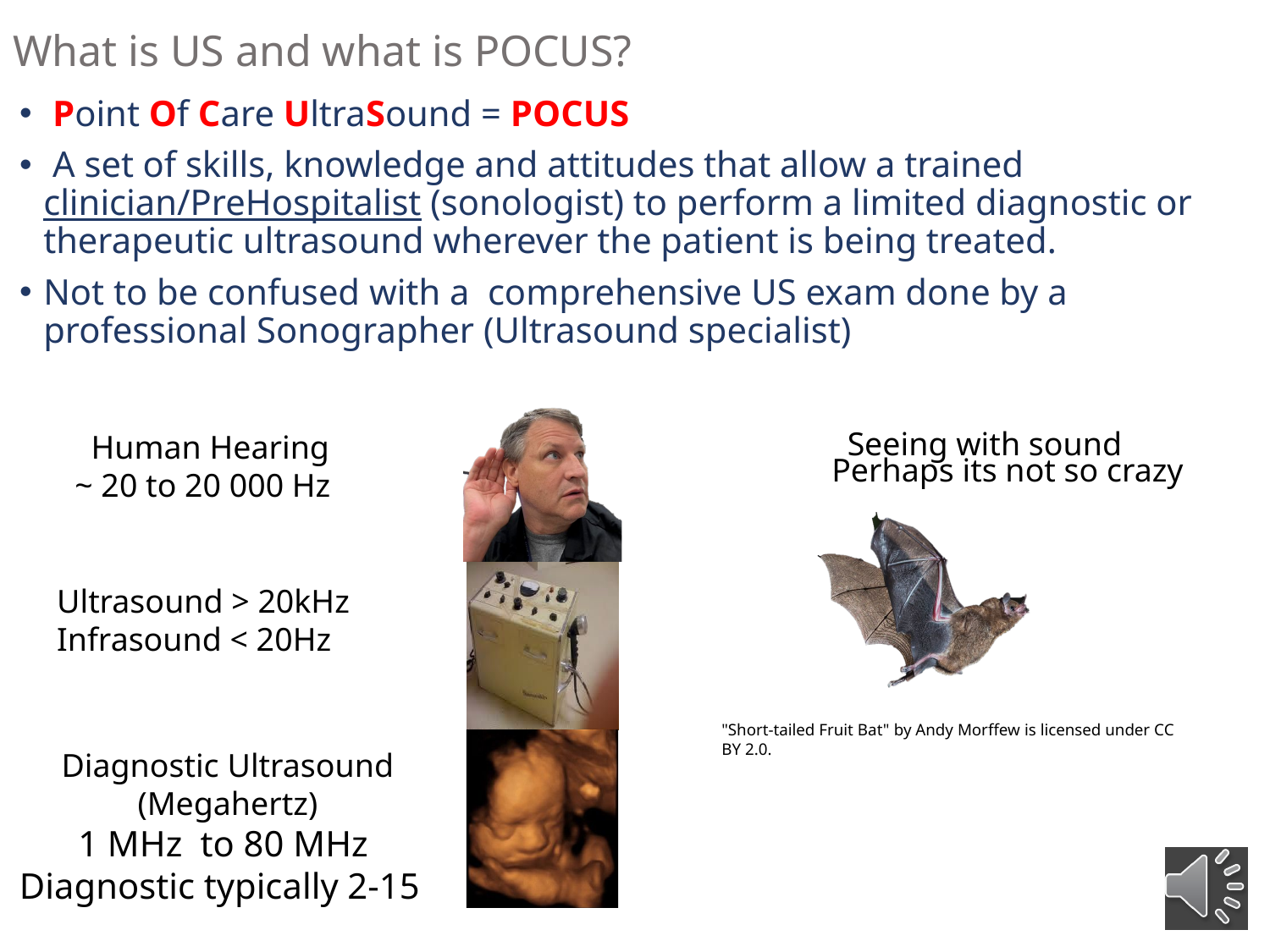

What is US and what is POCUS?
 Point Of Care UltraSound = POCUS
 A set of skills, knowledge and attitudes that allow a trained clinician/PreHospitalist (sonologist) to perform a limited diagnostic or therapeutic ultrasound wherever the patient is being treated.
Not to be confused with a comprehensive US exam done by a professional Sonographer (Ultrasound specialist)
Seeing with sound
 Human Hearing
~ 20 to 20 000 Hz
Perhaps its not so crazy
Ultrasound > 20kHz
Infrasound < 20Hz
"Short-tailed Fruit Bat" by Andy Morffew is licensed under CC BY 2.0.
Diagnostic Ultrasound (Megahertz)
1 MHz to 80 MHz
Diagnostic typically 2-15

## Slide 4
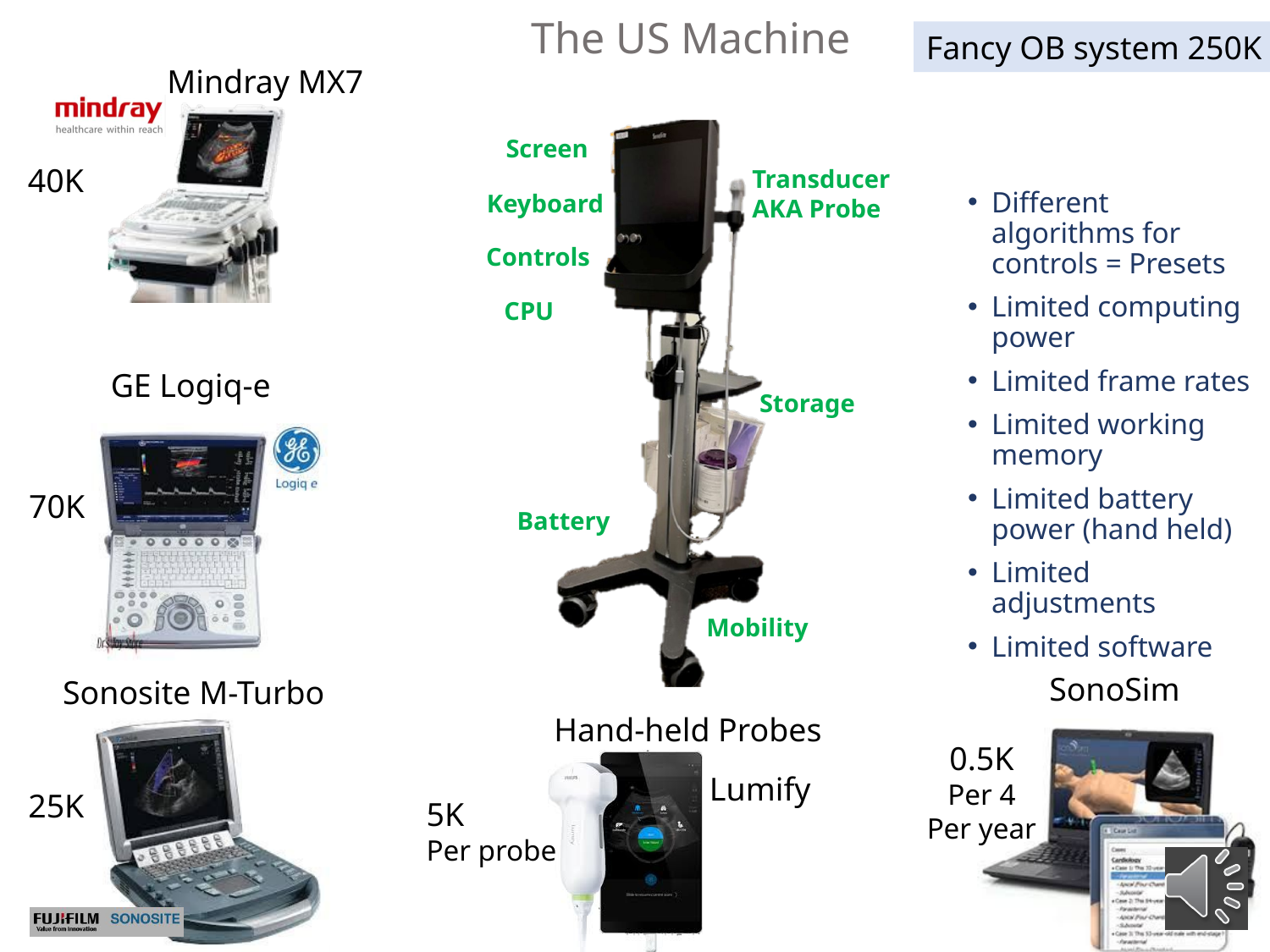

The US Machine
Fancy OB system 250K
Mindray MX7
Screen
40K
Transducer
AKA Probe
Keyboard
Different algorithms for controls = Presets
Limited computing power
Limited frame rates
Limited working memory
Limited battery power (hand held)
Limited adjustments
Limited software
Controls
CPU
GE Logiq-e
Storage
70K
Battery
Mobility
SonoSim
Sonosite M-Turbo
Hand-held Probes
0.5K
Per 4
Per year
Lumify
25K
5K
Per probe

## Slide 5
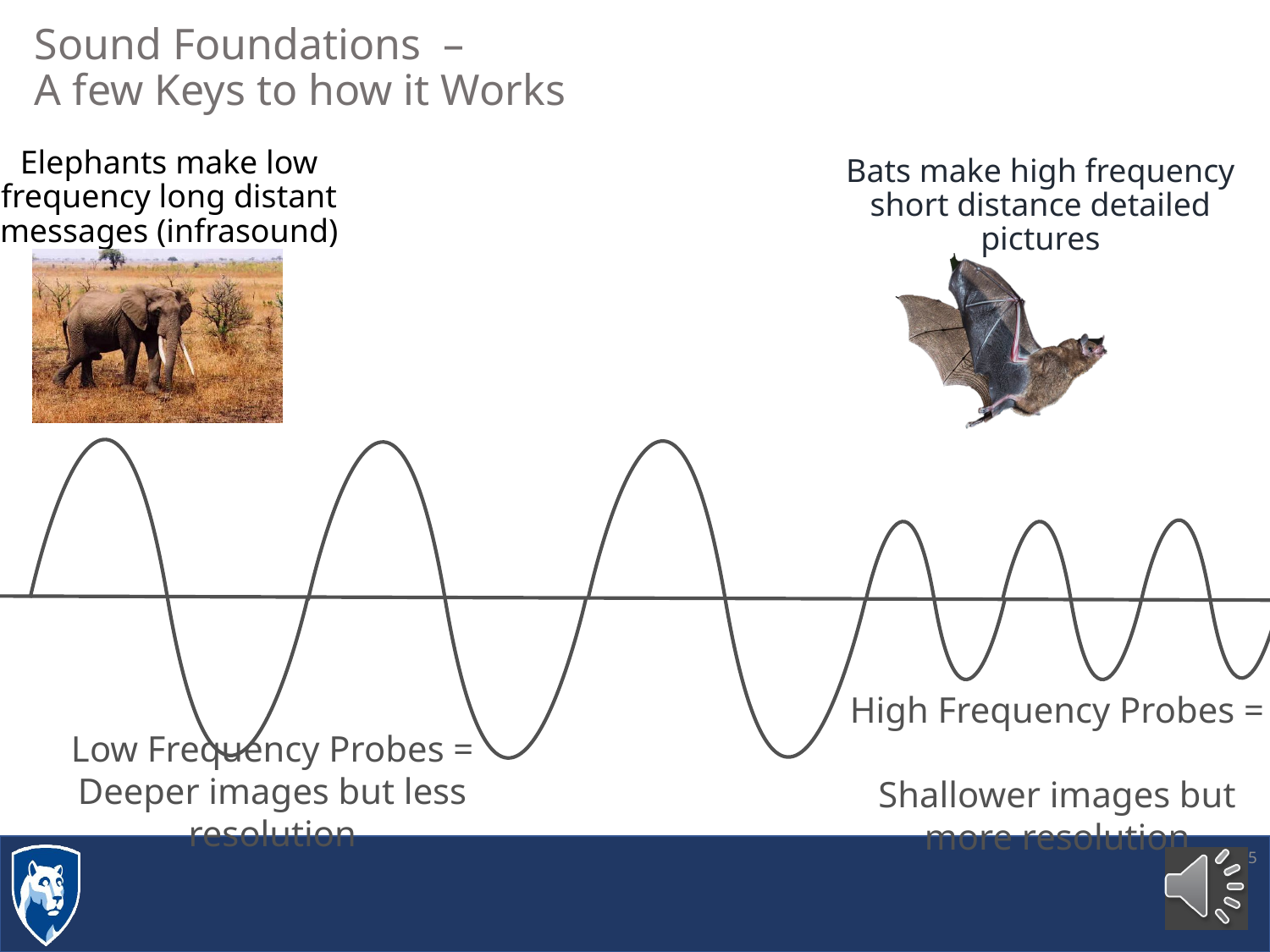

# Sound Foundations – A few Keys to how it Works
Elephants make low frequency long distant messages (infrasound)
Bats make high frequency short distance detailed pictures
High Frequency Probes =
Shallower images but more resolution
Low Frequency Probes =
Deeper images but less resolution
5

## Slide 6
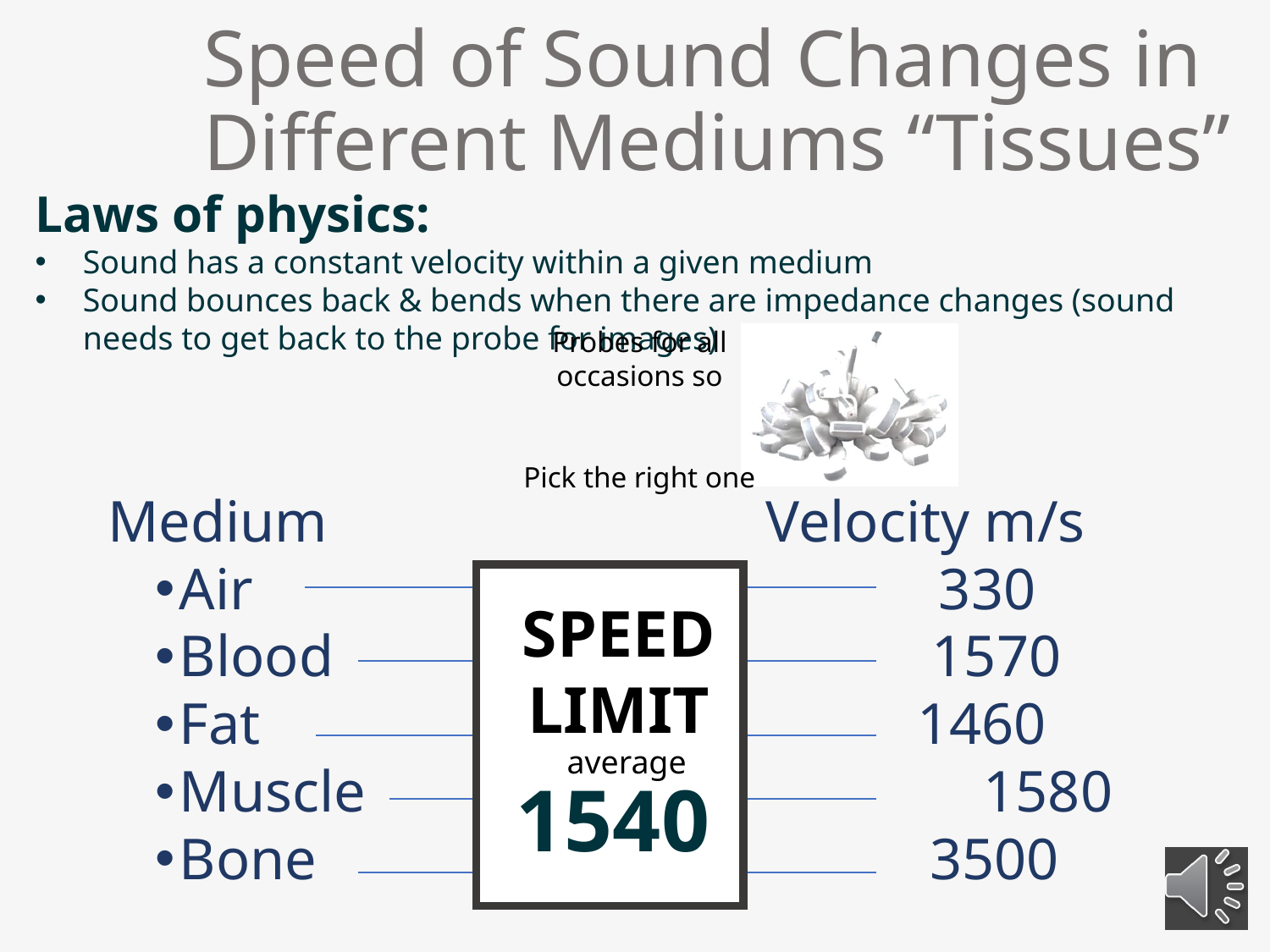

Speed of Sound Changes in
Different Mediums “Tissues”
Laws of physics:
Sound has a constant velocity within a given medium
Sound bounces back & bends when there are impedance changes (sound needs to get back to the probe for images)
Probes for all
occasions so
Pick the right one
Medium Velocity m/s
Air 330
Blood 1570
Fat 1460
Muscle		 1580
Bone 3500
SPEED LIMIT
average
1540

## Slide 7
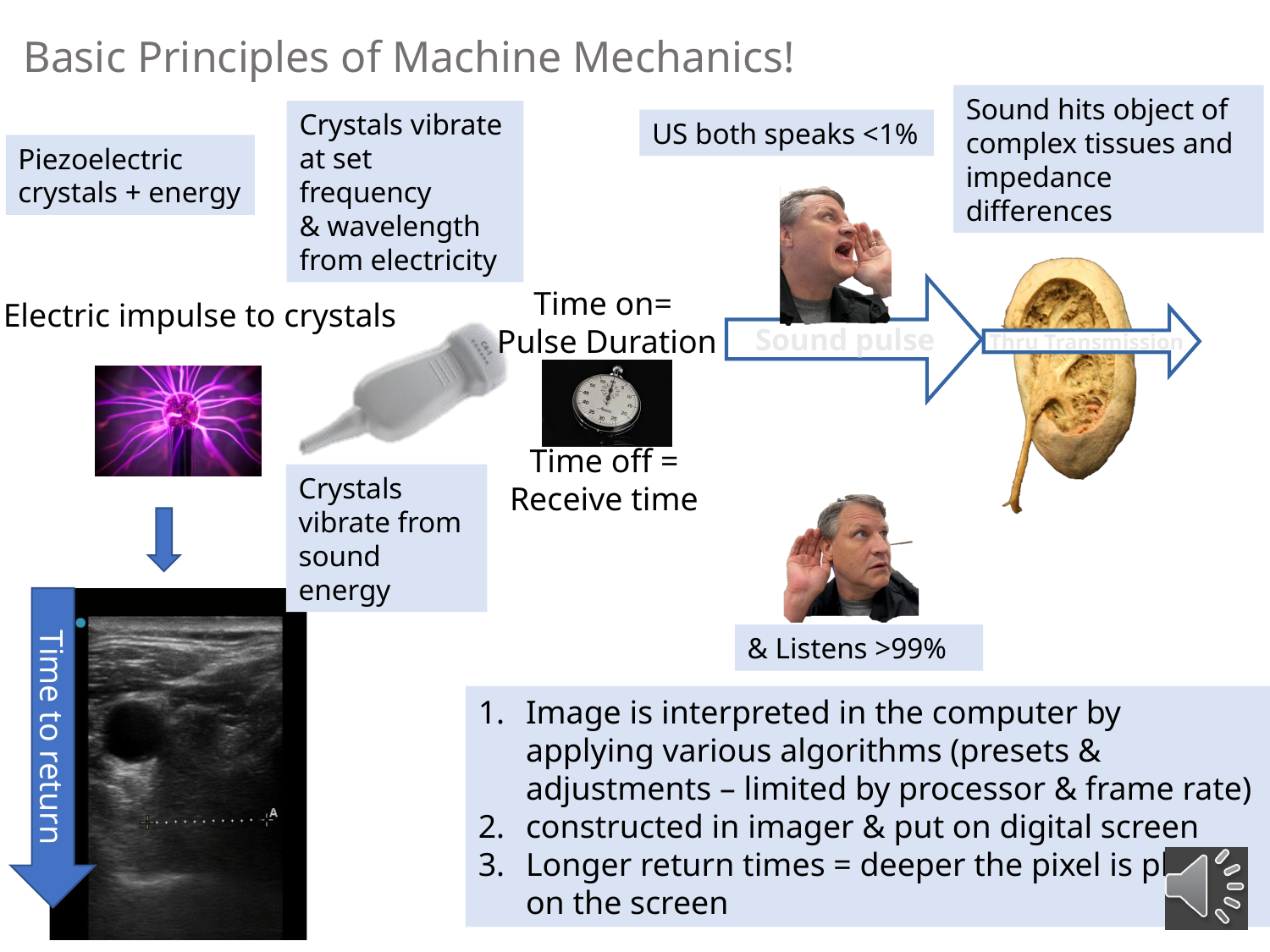

# Basic Principles of Machine Mechanics!
Sound hits object of complex tissues and impedance differences
Crystals vibrate at set frequency
& wavelength from electricity
US both speaks <1%
Piezoelectric crystals + energy
Sound pulse
Time on=
Pulse Duration
Electric impulse to crystals
Thru Transmission
Bounce Back
Time off =
Receive time
Crystals vibrate from sound energy
& Listens >99%
Image is interpreted in the computer by applying various algorithms (presets & adjustments – limited by processor & frame rate)
constructed in imager & put on digital screen
Longer return times = deeper the pixel is placed on the screen
Time to return
7

## Slide 8
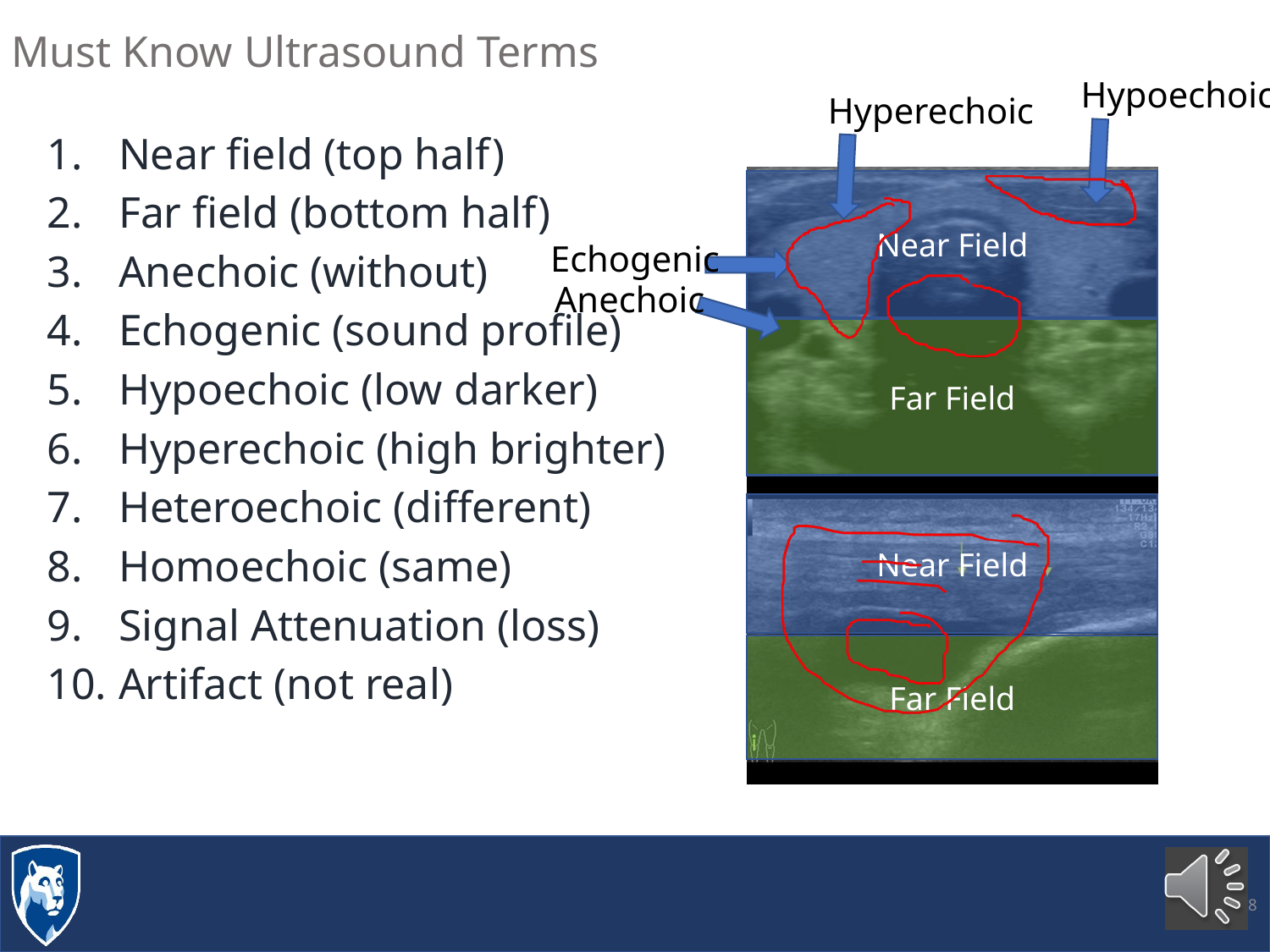

# Must Know Ultrasound Terms
Hypoechoic
Hyperechoic
Near field (top half)
Far field (bottom half)
Anechoic (without)
Echogenic (sound profile)
Hypoechoic (low darker)
Hyperechoic (high brighter)
Heteroechoic (different)
Homoechoic (same)
Signal Attenuation (loss)
Artifact (not real)
Near Field
Echogenic
Anechoic
Far Field
Near Field
Far Field
8

## Slide 9
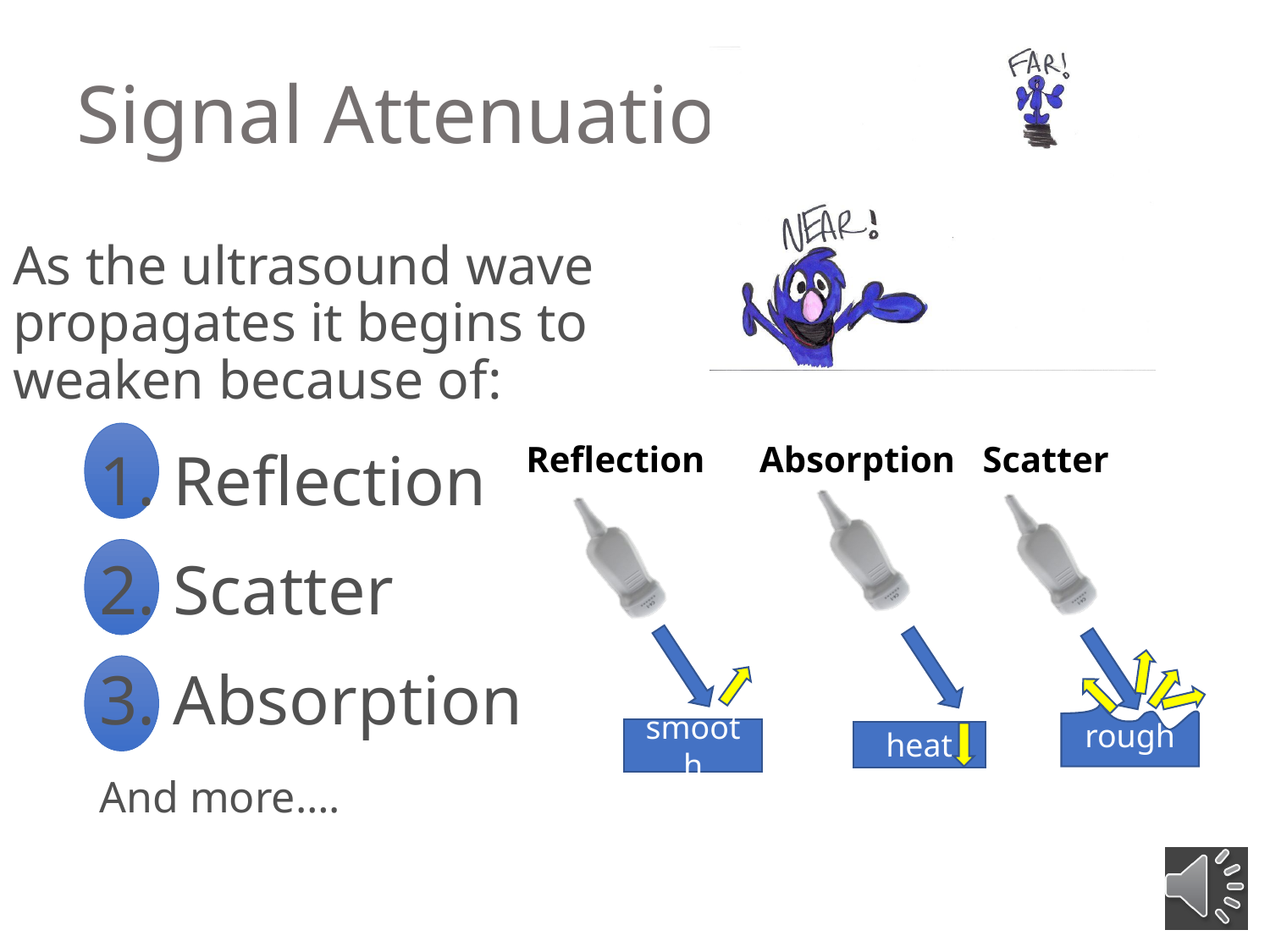

# Signal Attenuation
As the ultrasound wave propagates it begins to weaken because of:
1. Reflection
2. Scatter
3. Absorption
And more….
Reflection Absorption Scatter
rough
smooth
heat

## Slide 10
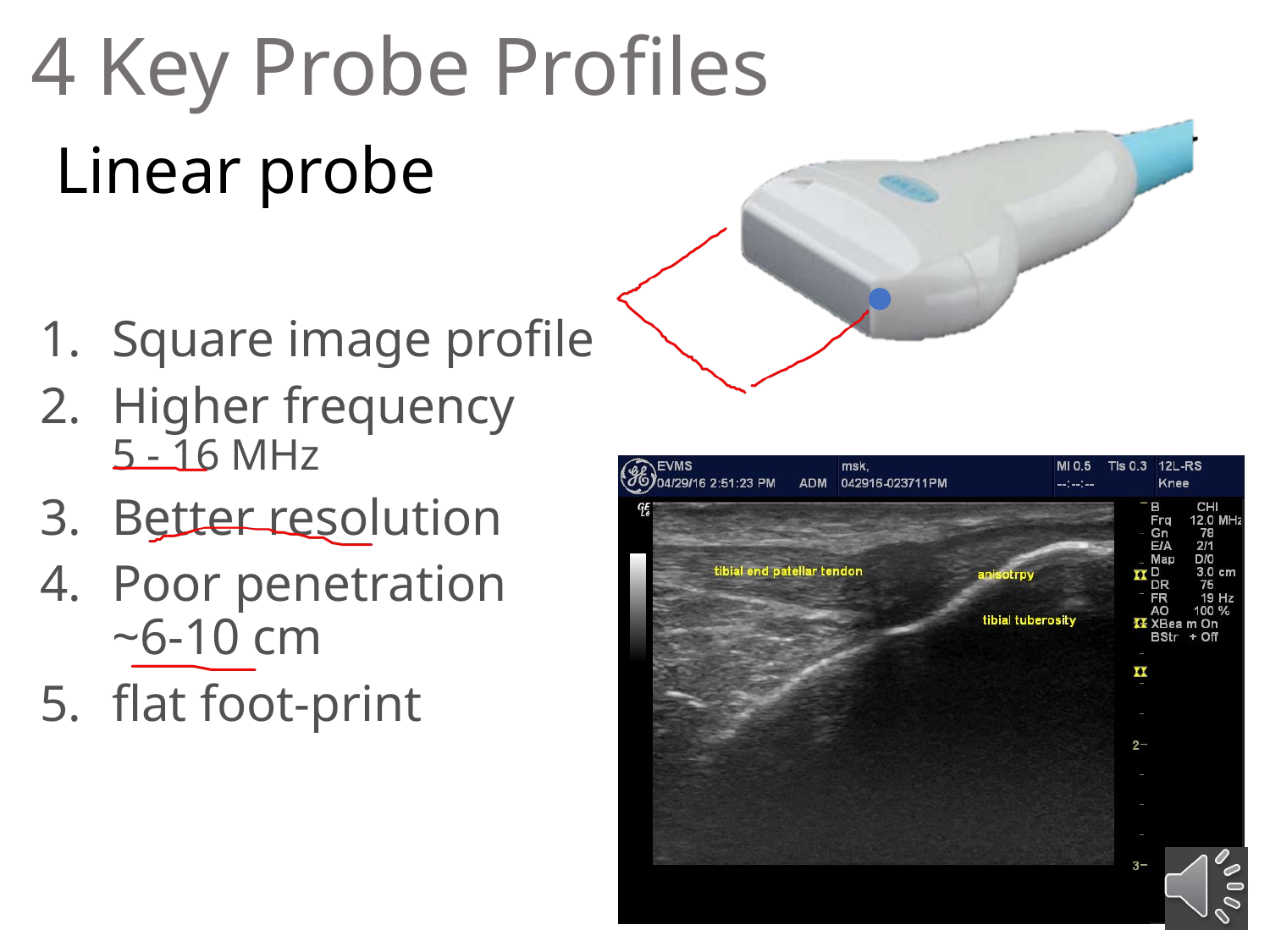

# 4 Key Probe Profiles
Linear probe
Square image profile
Higher frequency
5 - 16 MHz
Better resolution
Poor penetration
~6-10 cm
flat foot-print

## Slide 11
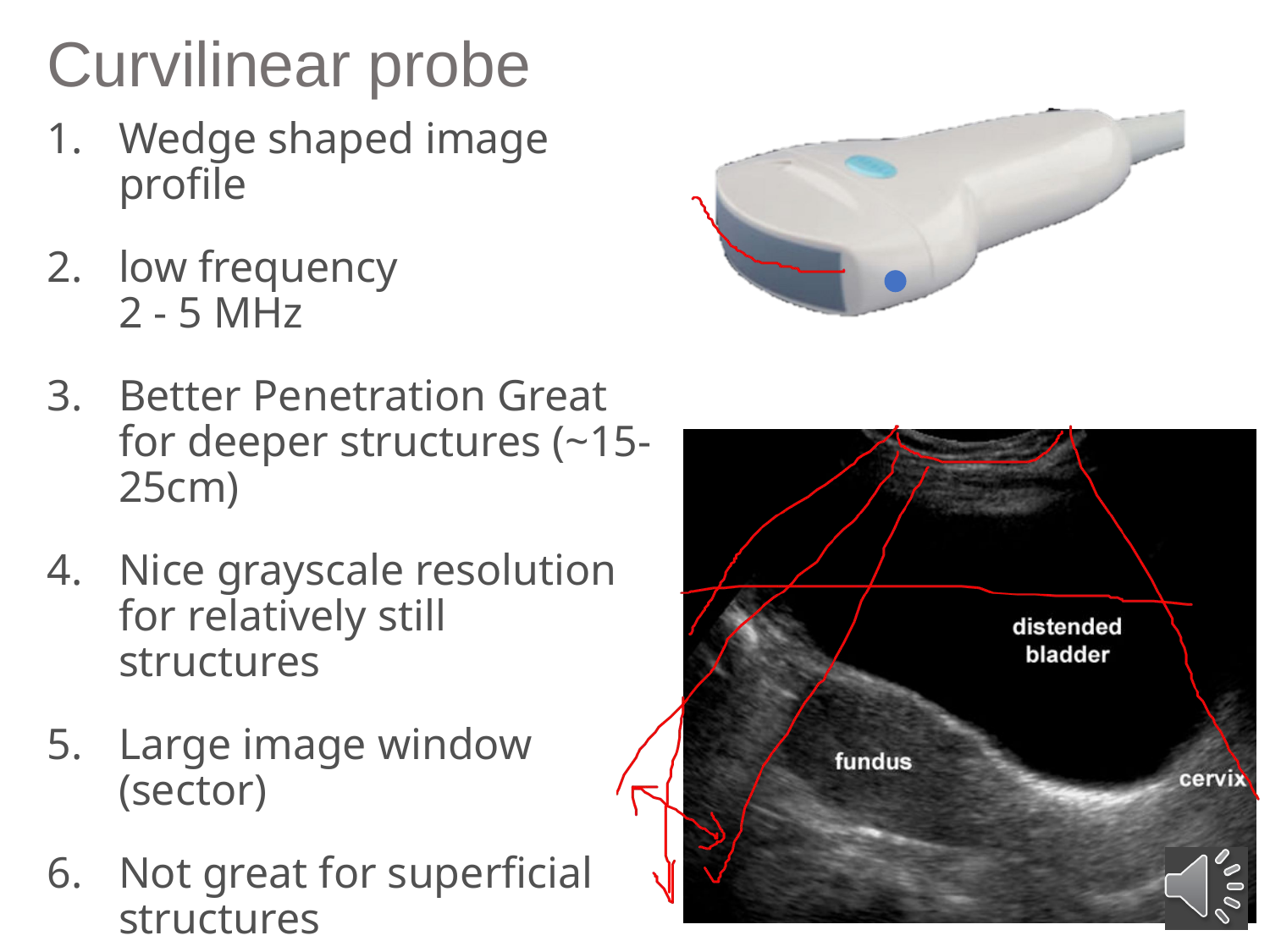

# Curvilinear probe
Wedge shaped image profile
low frequency
2 - 5 MHz
Better Penetration Great for deeper structures (~15-25cm)
Nice grayscale resolution for relatively still structures
Large image window (sector)
Not great for superficial structures
Curved footprint can make contact harder

## Slide 12
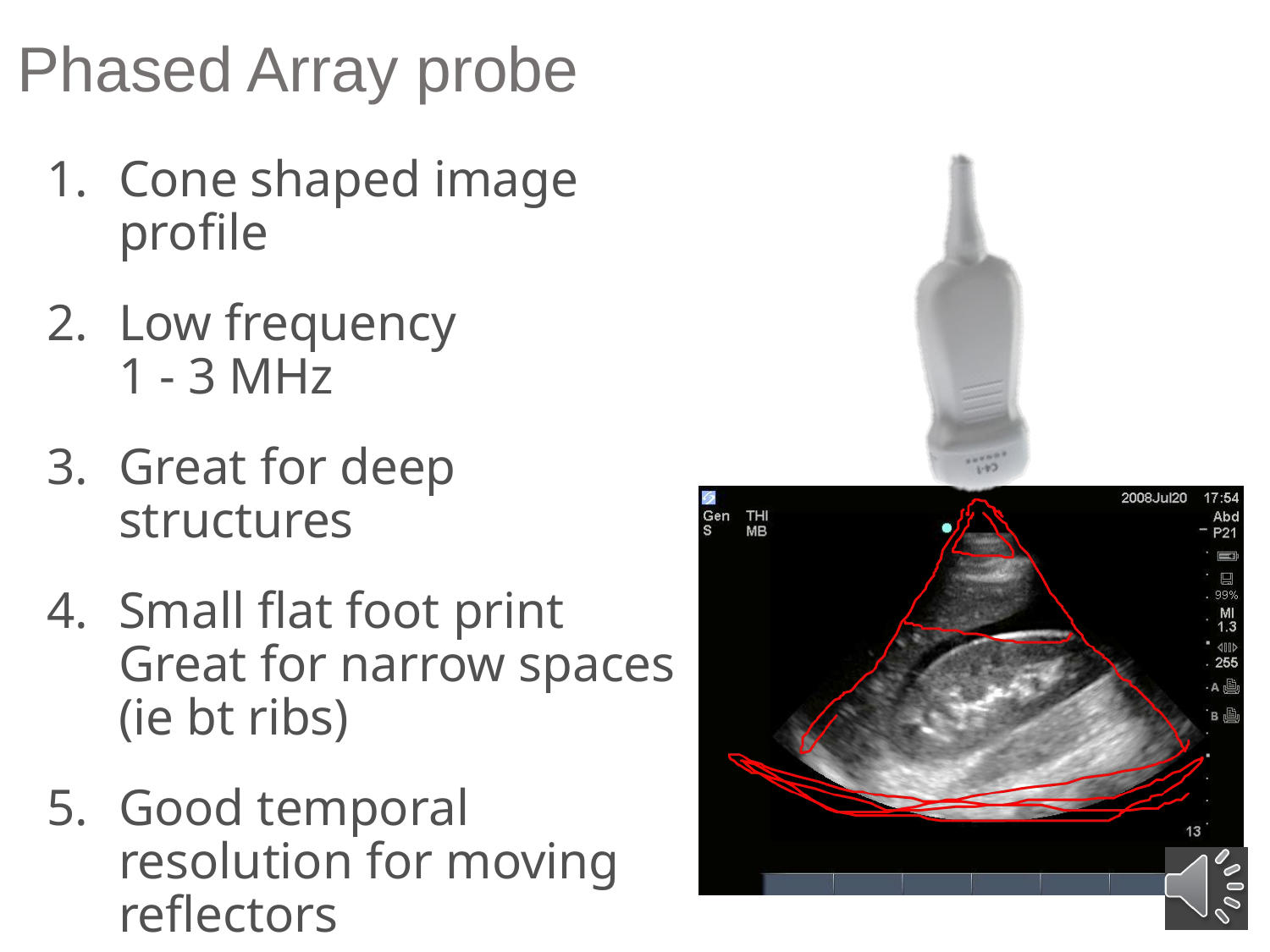

# Phased Array probe
Cone shaped image profile
Low frequency
1 - 3 MHz
Great for deep structures
Small flat foot print Great for narrow spaces (ie bt ribs)
Good temporal resolution for moving reflectors
Not good for superficial stuff at all
Terrible near field resolution

## Slide 13
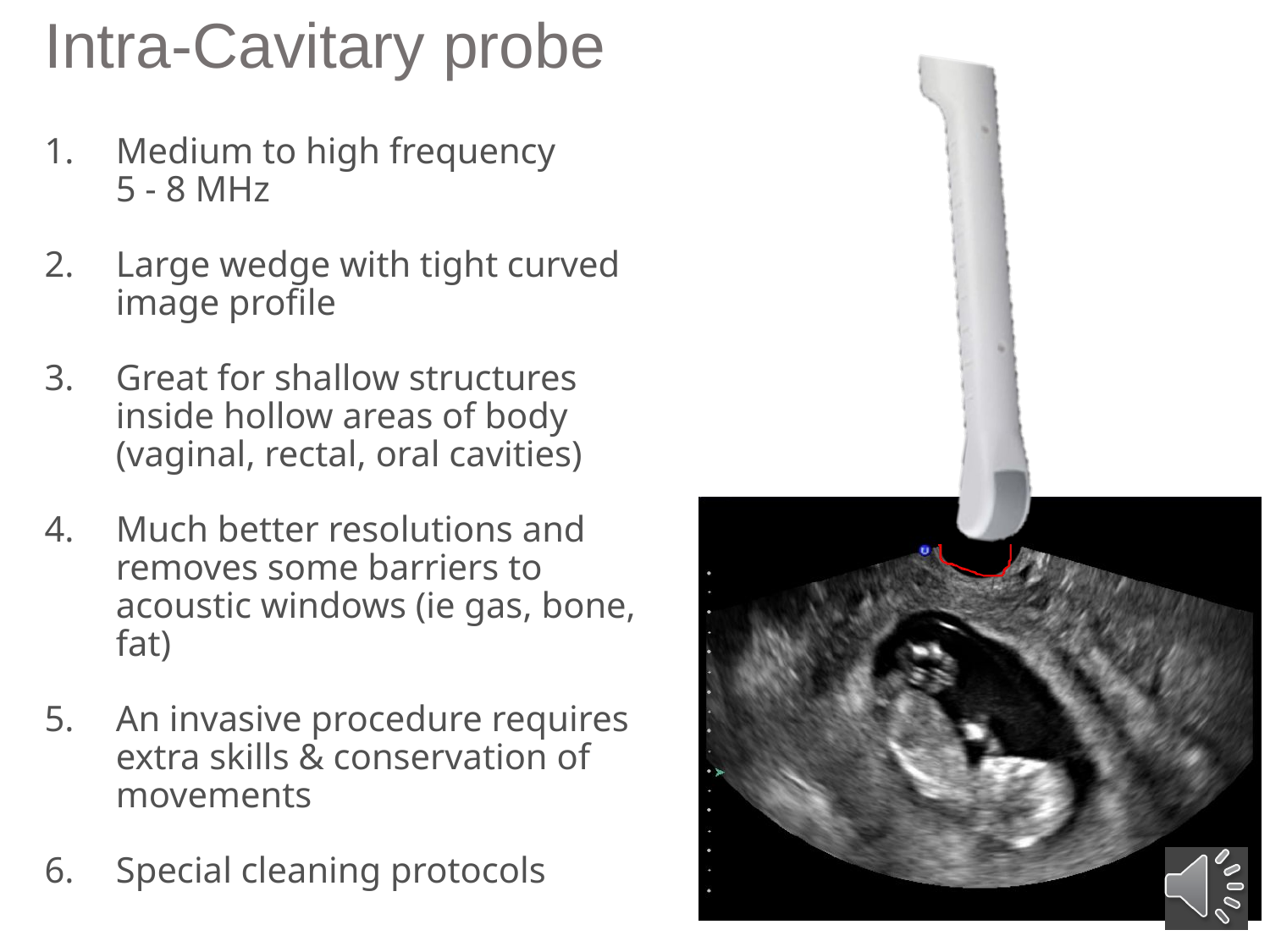

# Intra-Cavitary probe
Medium to high frequency
5 - 8 MHz
Large wedge with tight curved image profile
Great for shallow structures inside hollow areas of body (vaginal, rectal, oral cavities)
Much better resolutions and removes some barriers to acoustic windows (ie gas, bone, fat)
An invasive procedure requires extra skills & conservation of movements
Special cleaning protocols

## Slide 14
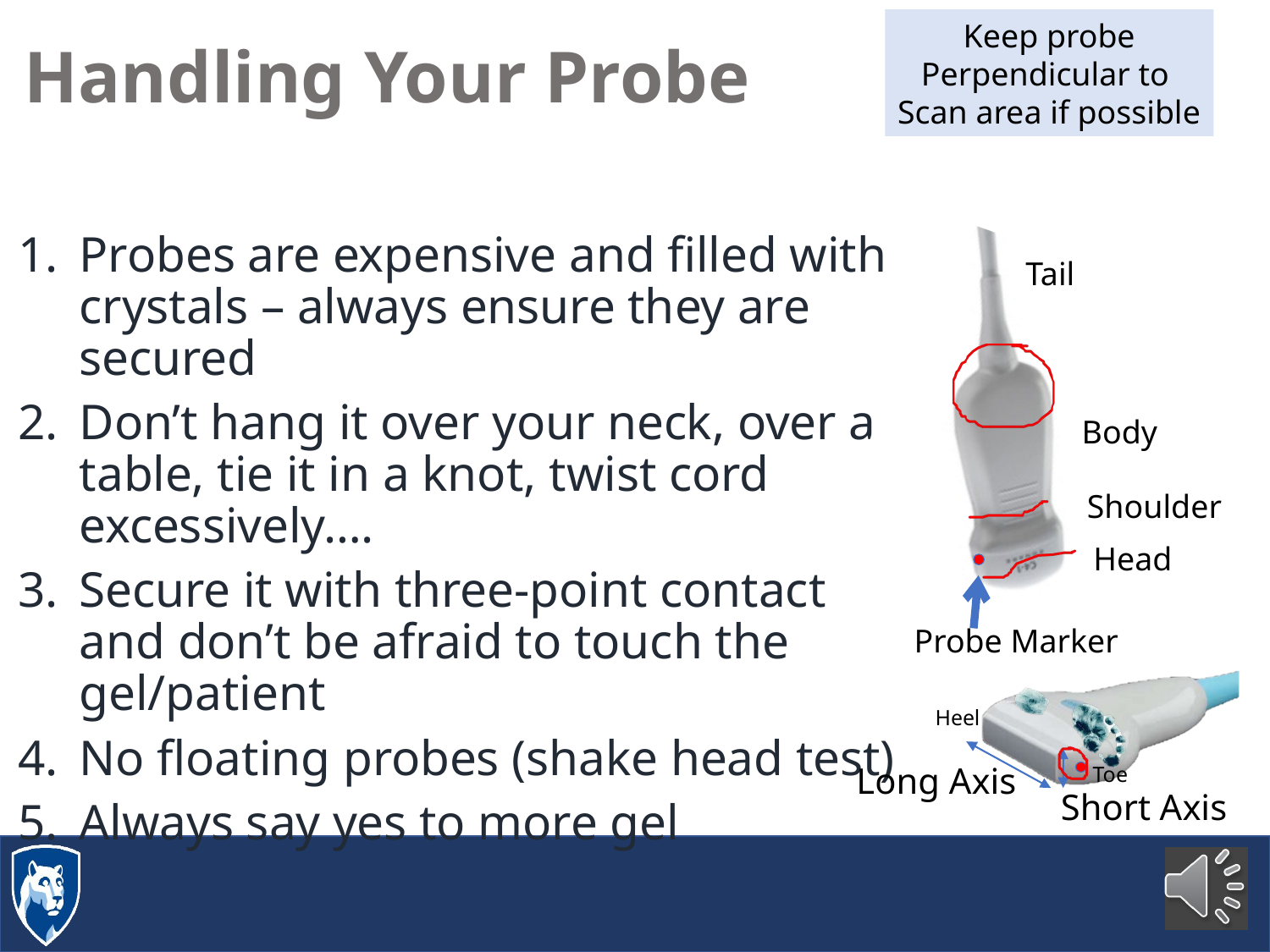

# Handling Your Probe
Keep probe
Perpendicular to
Scan area if possible
Probes are expensive and filled with crystals – always ensure they are secured
Don’t hang it over your neck, over a table, tie it in a knot, twist cord excessively….
Secure it with three-point contact and don’t be afraid to touch the gel/patient
No floating probes (shake head test)
Always say yes to more gel
Tail
Body
Shoulder
Head
Probe Marker
Heel
Long Axis
Toe
Short Axis

## Slide 15
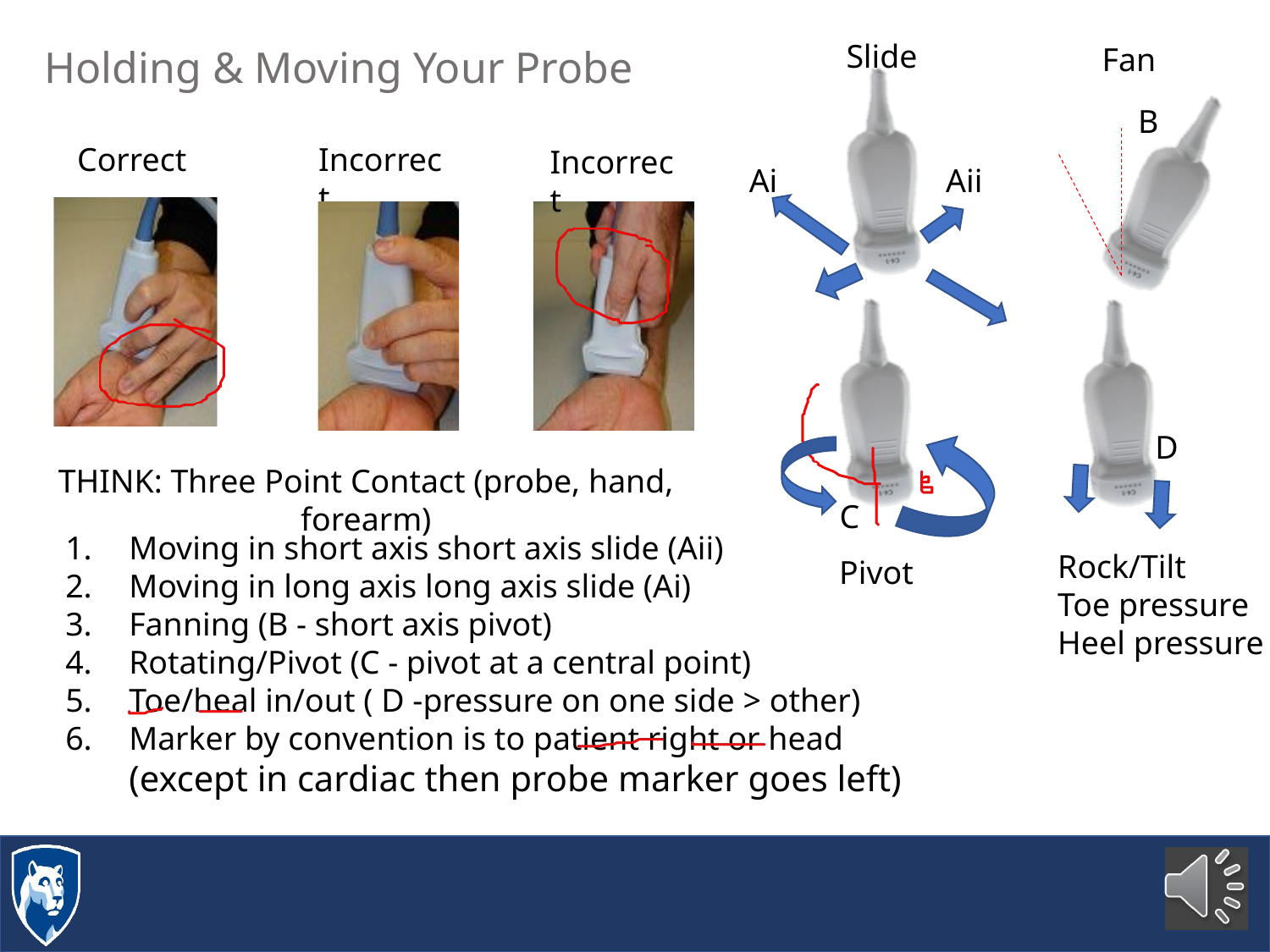

# Holding & Moving Your Probe
Slide
Fan
B
Correct
Incorrect
Incorrect
Ai
Aii
D
THINK: Three Point Contact (probe, hand, forearm)
C
Moving in short axis short axis slide (Aii)
Moving in long axis long axis slide (Ai)
Fanning (B - short axis pivot)
Rotating/Pivot (C - pivot at a central point)
Toe/heal in/out ( D -pressure on one side > other)
Marker by convention is to patient right or head
(except in cardiac then probe marker goes left)
Rock/Tilt
Toe pressure
Heel pressure
Pivot

## Slide 16
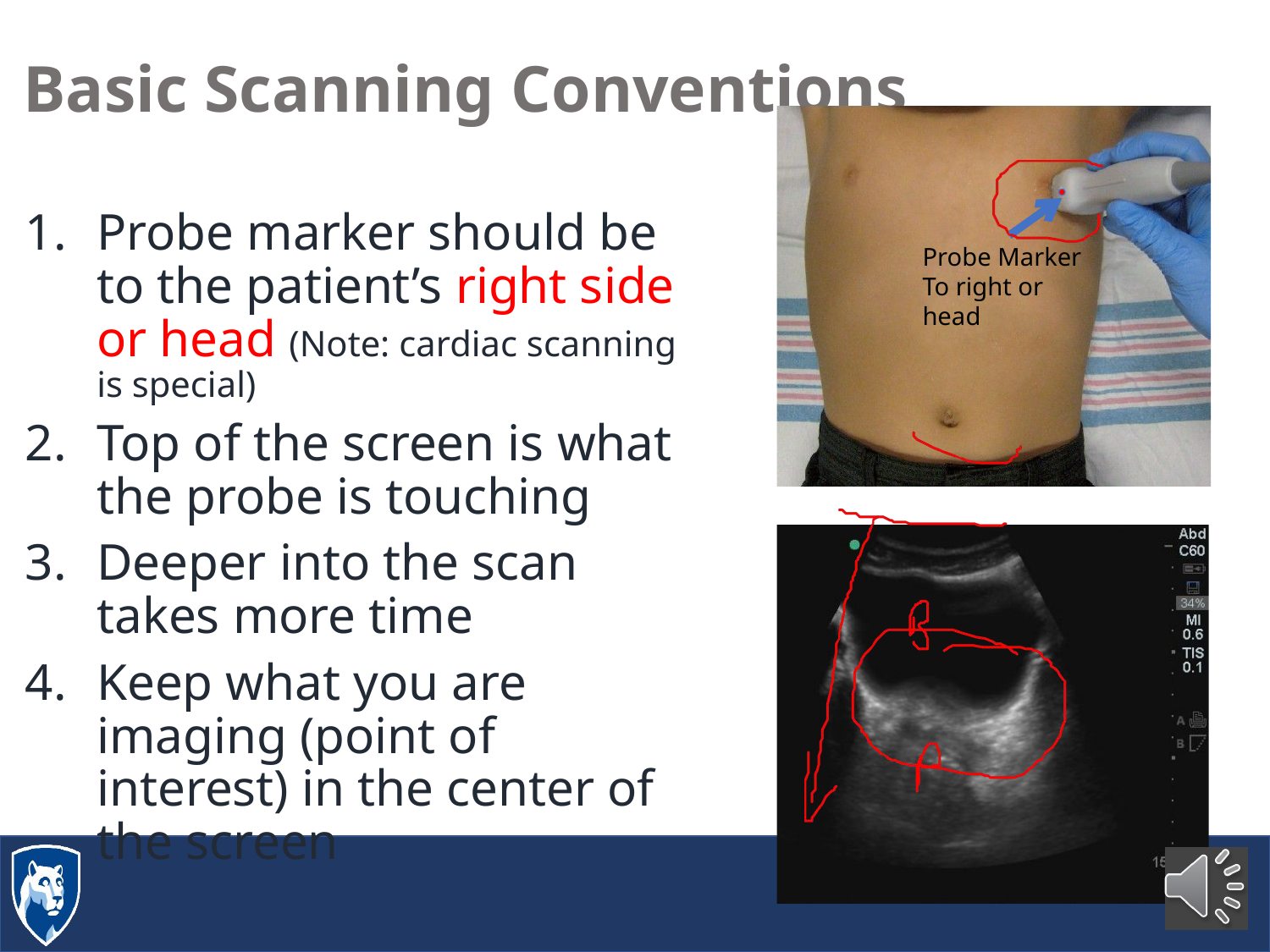

# Basic Scanning Conventions
Probe Marker
To right or head
Probe marker should be to the patient’s right side or head (Note: cardiac scanning is special)
Top of the screen is what the probe is touching
Deeper into the scan takes more time
Keep what you are imaging (point of interest) in the center of the screen

## Slide 17
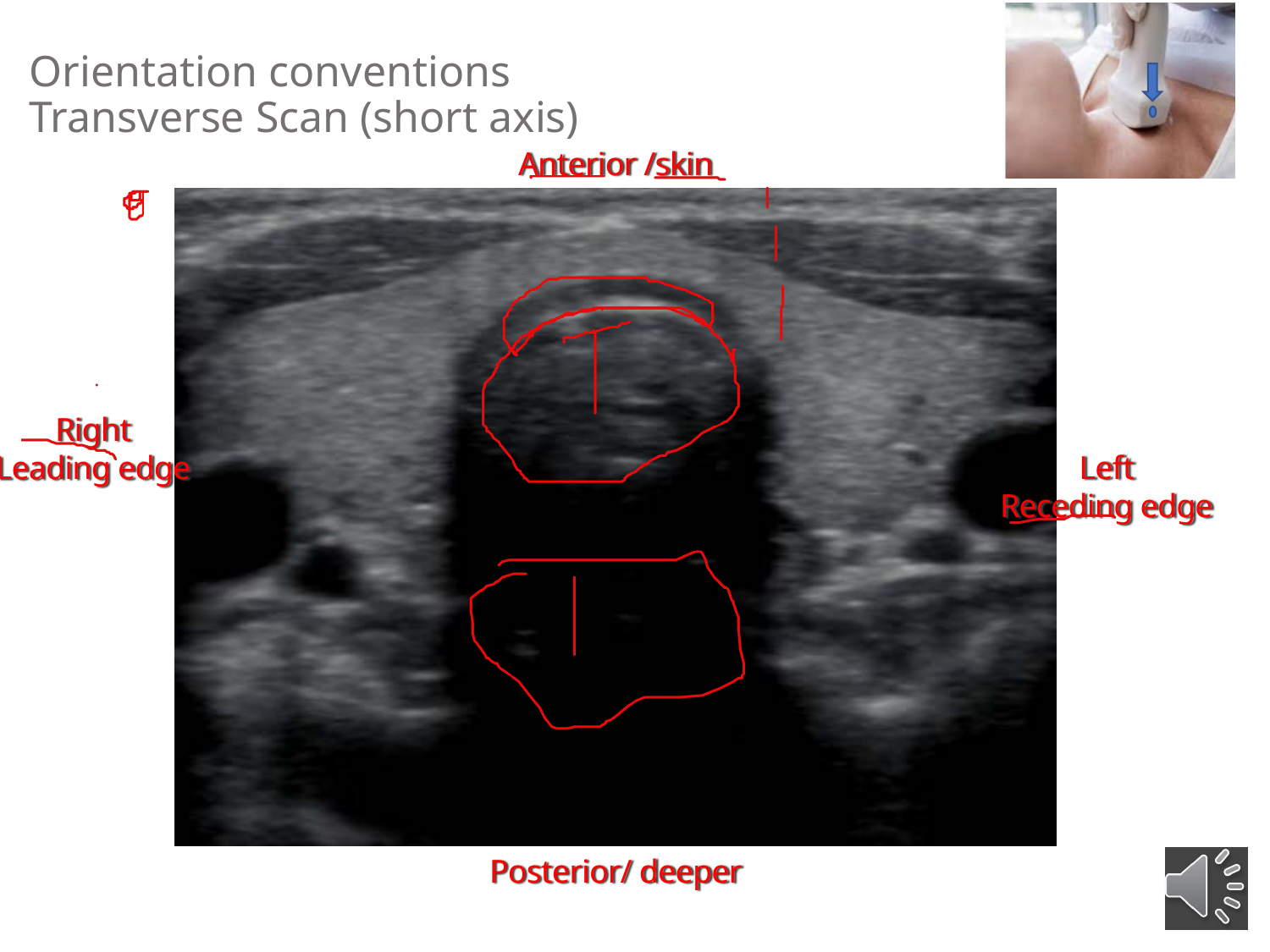

# Orientation conventions Transverse Scan (short axis)
Anterior /skin
Right
Leading edge
Left
Receding edge
Posterior/ deeper

## Slide 18
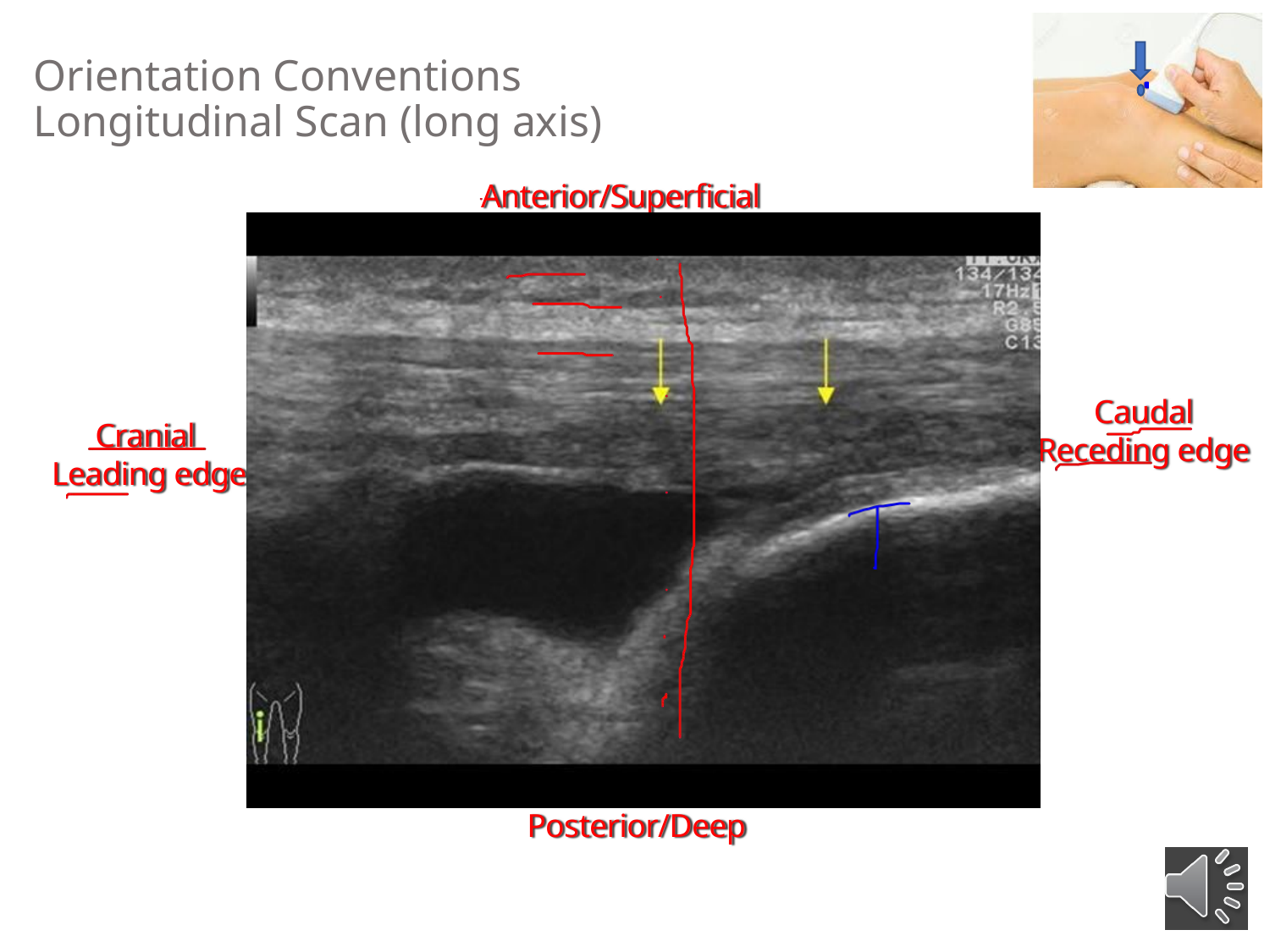

# Orientation Conventions Longitudinal Scan (long axis)
Anterior/Superficial
Caudal
Receding edge
Cranial
Leading edge
Posterior/Deep

## Slide 19
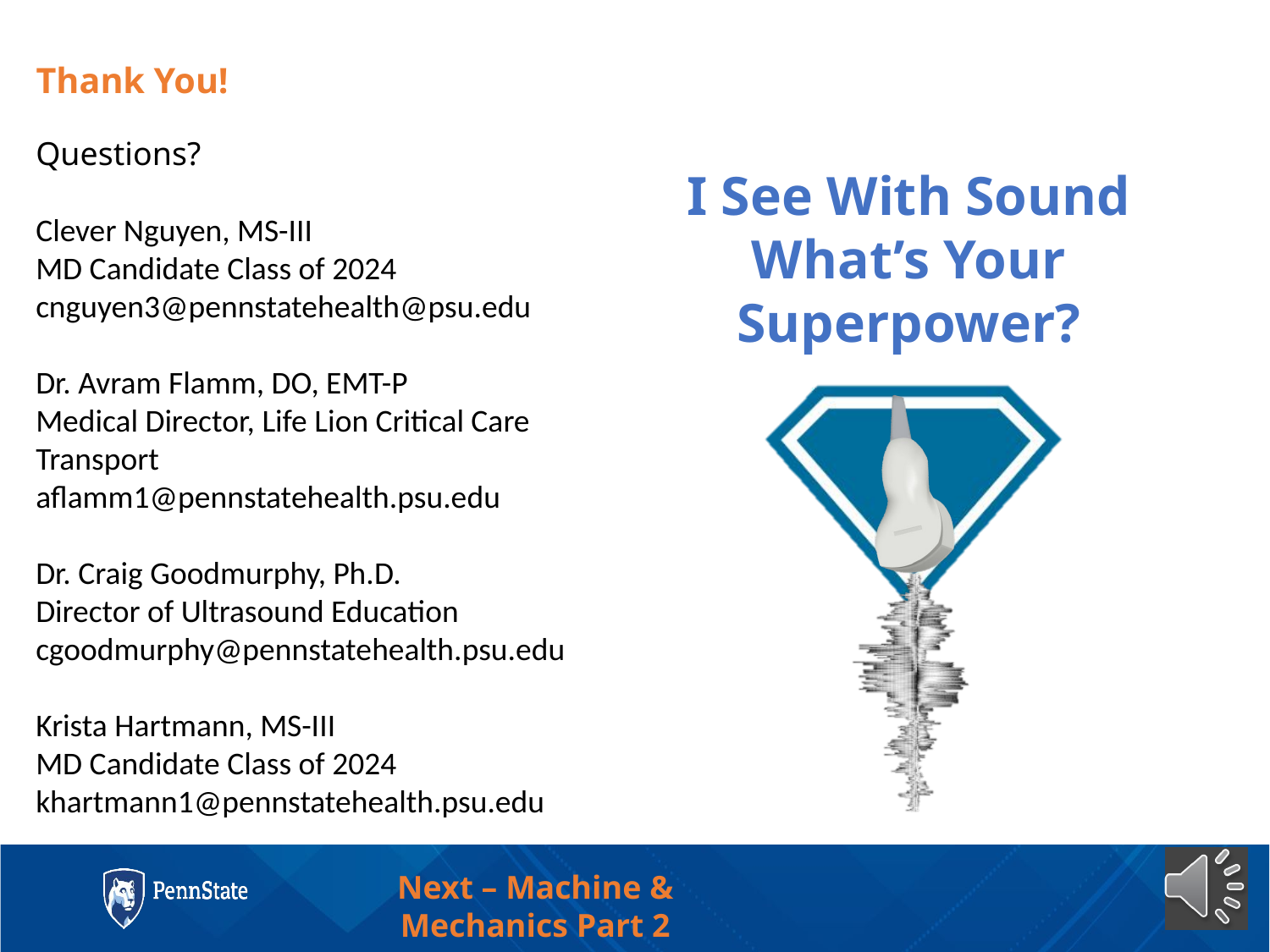

# Thank You!
Questions?
Clever Nguyen, MS-III
MD Candidate Class of 2024
cnguyen3@pennstatehealth@psu.edu
Dr. Avram Flamm, DO, EMT-P
Medical Director, Life Lion Critical Care Transport
aflamm1@pennstatehealth.psu.edu
Dr. Craig Goodmurphy, Ph.D.
Director of Ultrasound Education
cgoodmurphy@pennstatehealth.psu.edu
Krista Hartmann, MS-III
MD Candidate Class of 2024
khartmann1@pennstatehealth.psu.edu
I See With Sound What’s Your Superpower?
Next – Machine & Mechanics Part 2
