# Supplementary material for: E-FAST Ultrasound Training Curriculum for Prehospital Emergency Medical Service (EMS) Clinicians
Source: J Educ Teach Emerg Med. 2024 Jan 31;9(1):C41–97. doi: 10.21980/J8S060 (PMC10854885; doi:10.21980/J8S060)
Supplement: Supplementary file 13 — Please see associated Power Point Lecture Link: https://youtu.be/oc2v2DbcZyg [file jetem-9-1-C41-AppendixF.pptx]

## Slide 1
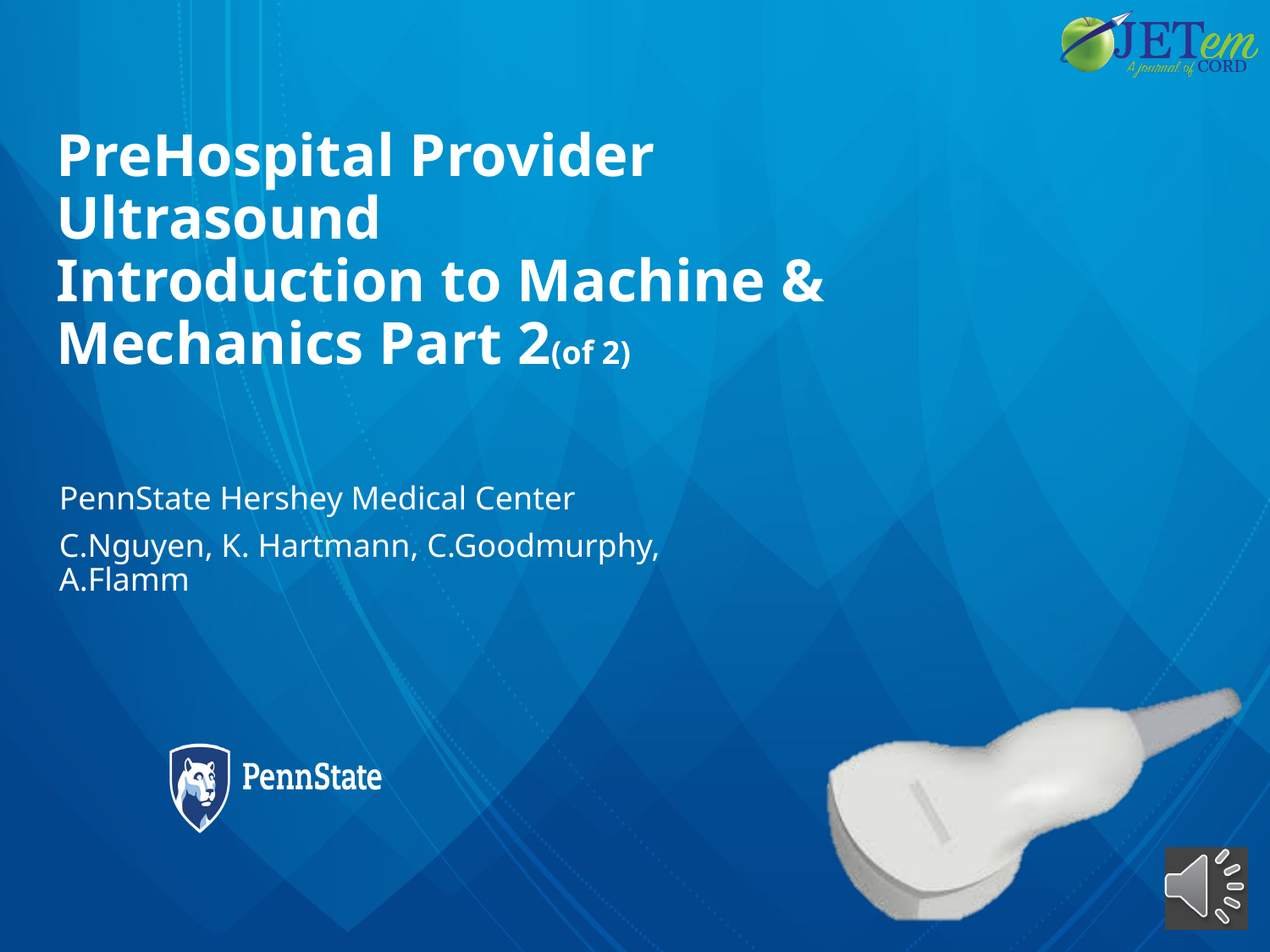

# PreHospital Provider Ultrasound Introduction to Machine & Mechanics Part 2(of 2)
PennState Hershey Medical Center
C.Nguyen, K. Hartmann, C.Goodmurphy, A.Flamm

## Slide 2
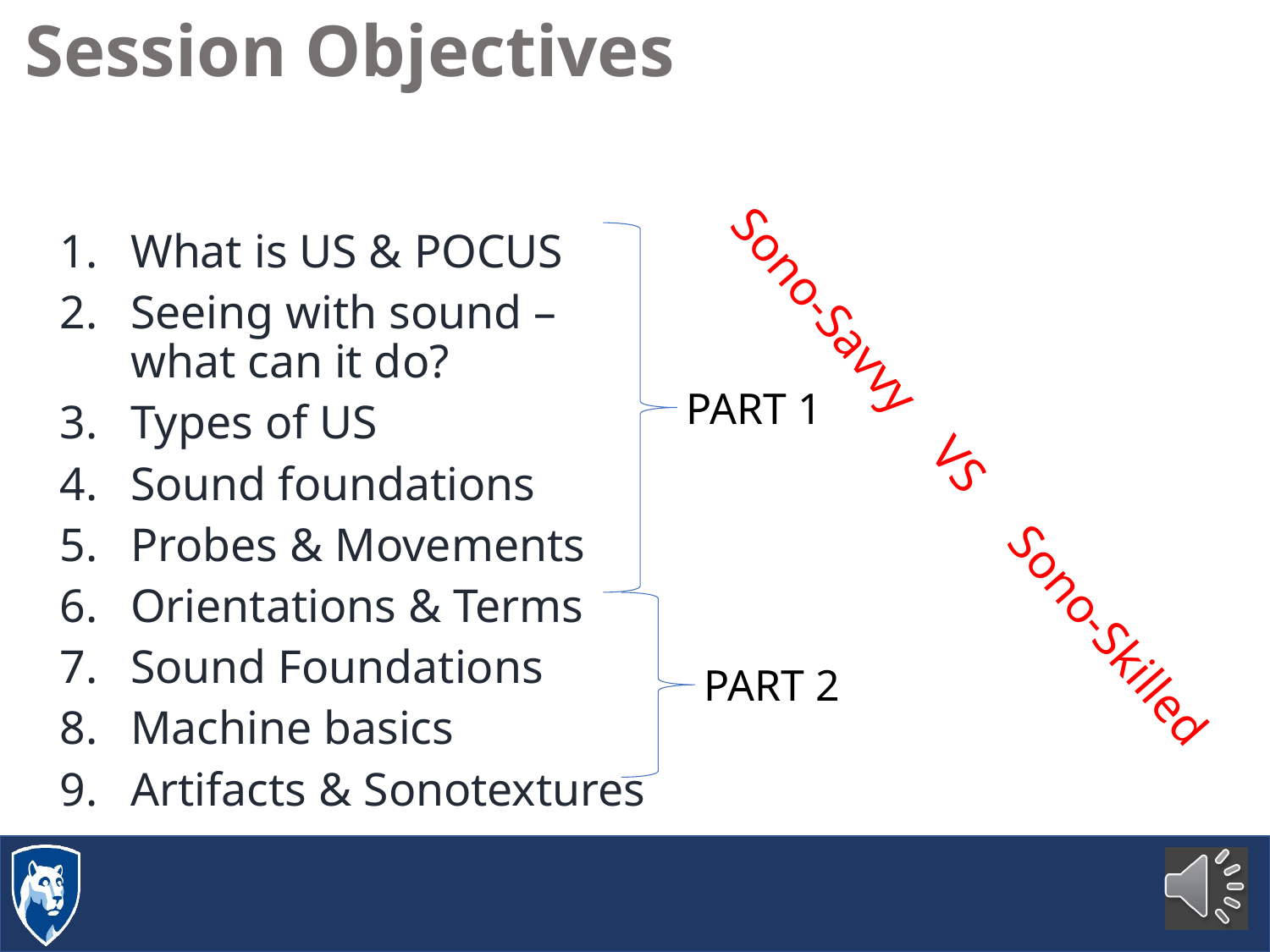

# Session Objectives
What is US & POCUS
Seeing with sound – what can it do?
Types of US
Sound foundations
Probes & Movements
Orientations & Terms
Sound Foundations
Machine basics
Artifacts & Sonotextures
PART 1
Sono-Savvy VS Sono-Skilled
PART 2

## Slide 3
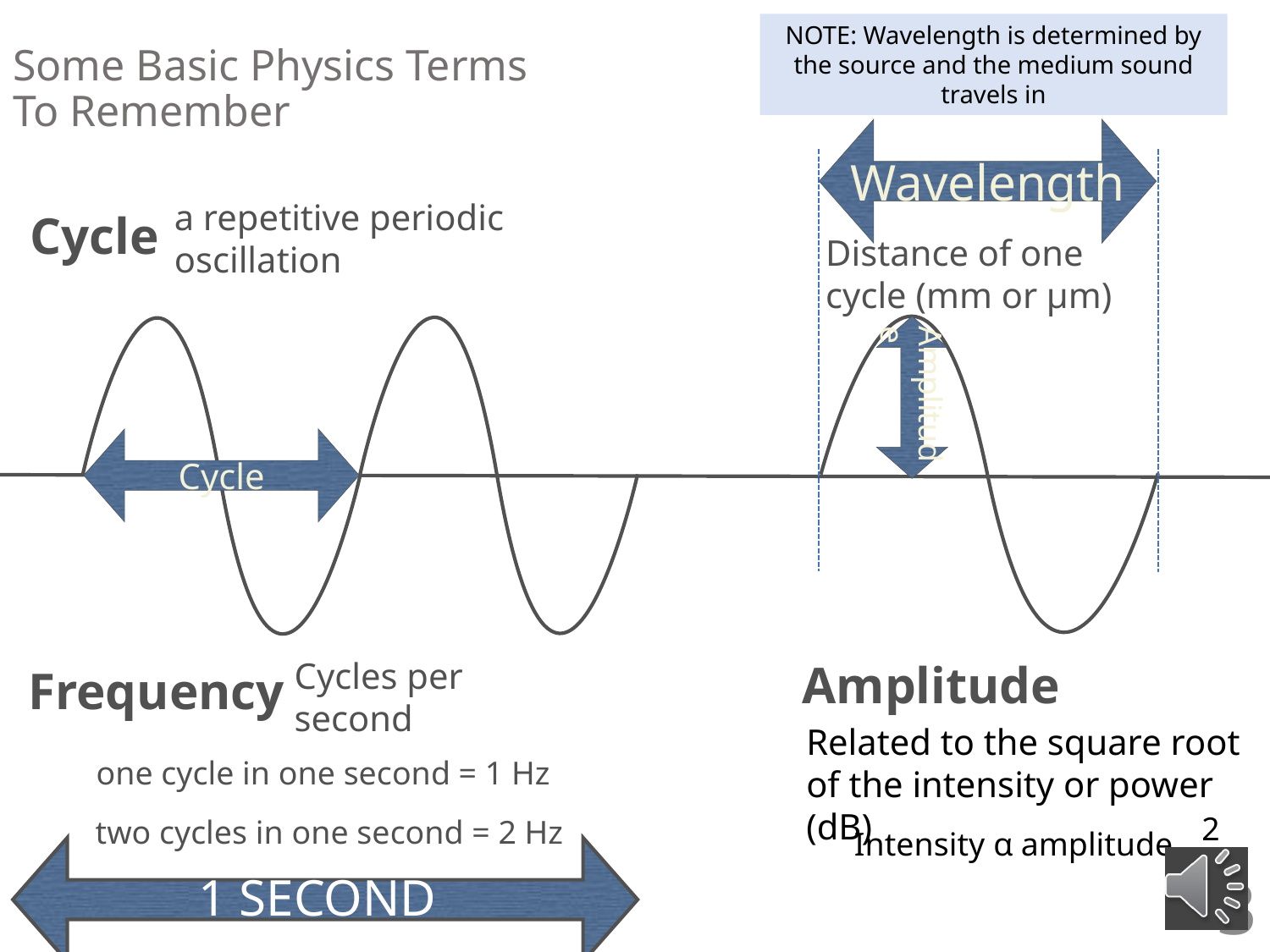

Some Basic Physics Terms To Remember
NOTE: Wavelength is determined by the source and the medium sound travels in
Wavelength
Cycle
a repetitive periodic oscillation
Distance of one cycle (mm or µm)
Amplitude
Cycle
Amplitude
Frequency
Cycles per second
Related to the square root of the intensity or power (dB)
one cycle in one second = 1 Hz
2
two cycles in one second = 2 Hz
Intensity α amplitude
1 SECOND
3

## Slide 4
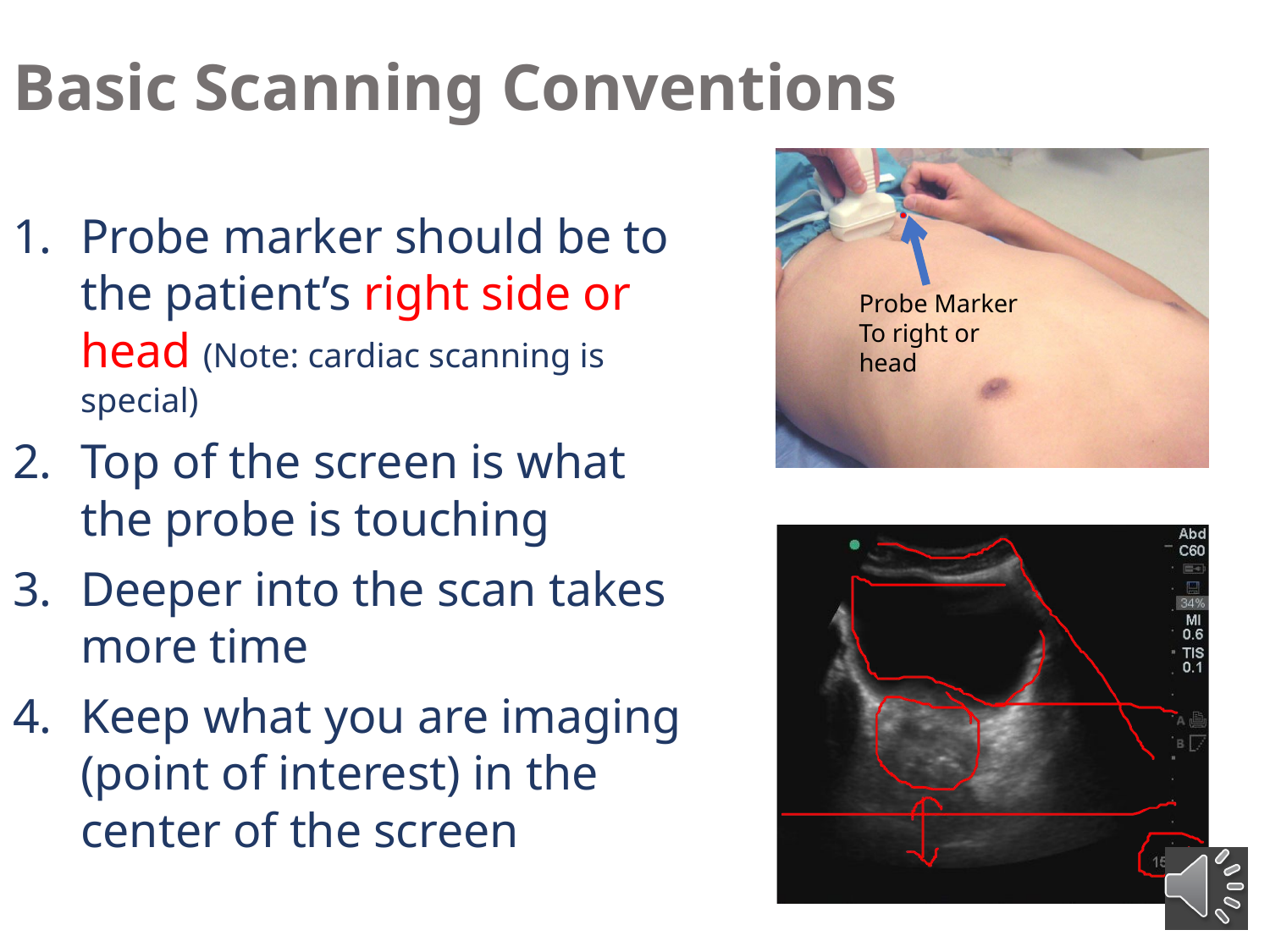

Basic Scanning Conventions
Probe marker should be to the patient’s right side or head (Note: cardiac scanning is special)
Top of the screen is what the probe is touching
Deeper into the scan takes more time
Keep what you are imaging (point of interest) in the center of the screen
Probe Marker
To right or head

## Slide 5
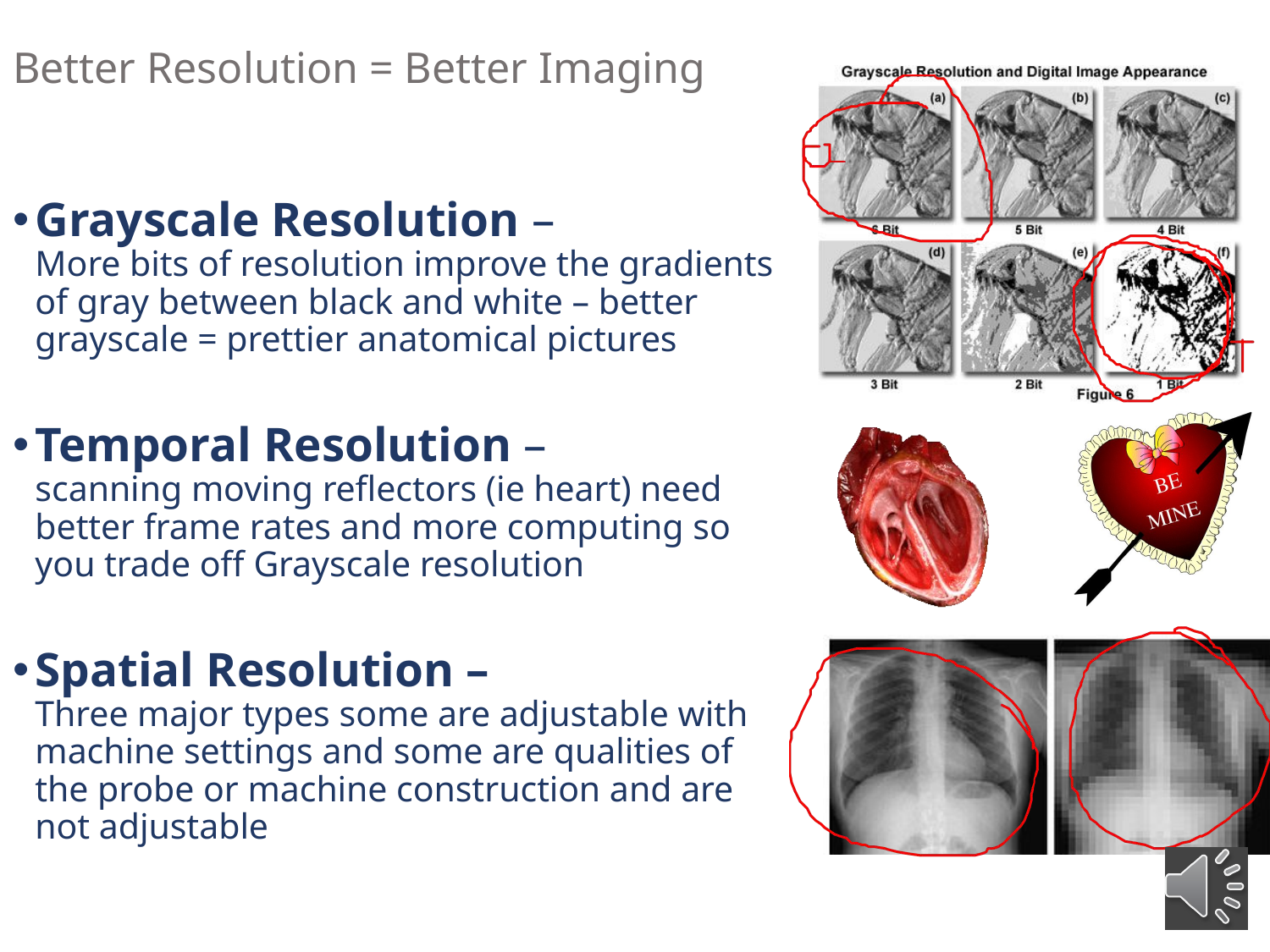

Better Resolution = Better Imaging
Grayscale Resolution –
More bits of resolution improve the gradients of gray between black and white – better grayscale = prettier anatomical pictures
Temporal Resolution –
scanning moving reflectors (ie heart) need better frame rates and more computing so you trade off Grayscale resolution
Spatial Resolution –
Three major types some are adjustable with machine settings and some are qualities of the probe or machine construction and are not adjustable

## Slide 6
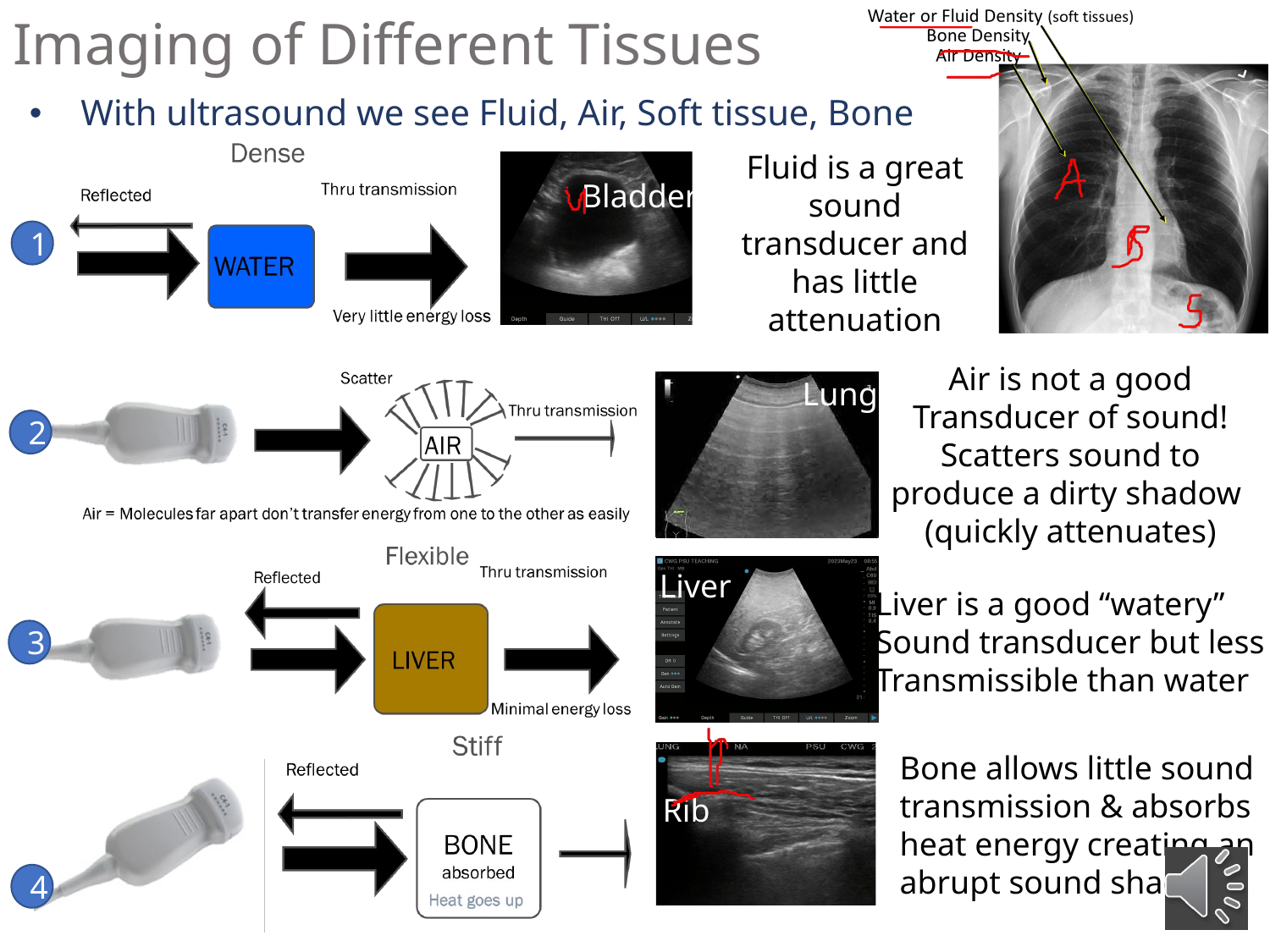

# Imaging of Different Tissues
With ultrasound we see Fluid, Air, Soft tissue, Bone
Fluid is a great
sound transducer and has little attenuation
Bladder
1
Air is not a good
Transducer of sound! Scatters sound to produce a dirty shadow
(quickly attenuates)
Lung
2
Liver
Liver is a good “watery”
Sound transducer but less
Transmissible than water
3
Bone allows little sound transmission & absorbs heat energy creating an abrupt sound shadow
Rib
4

## Slide 7
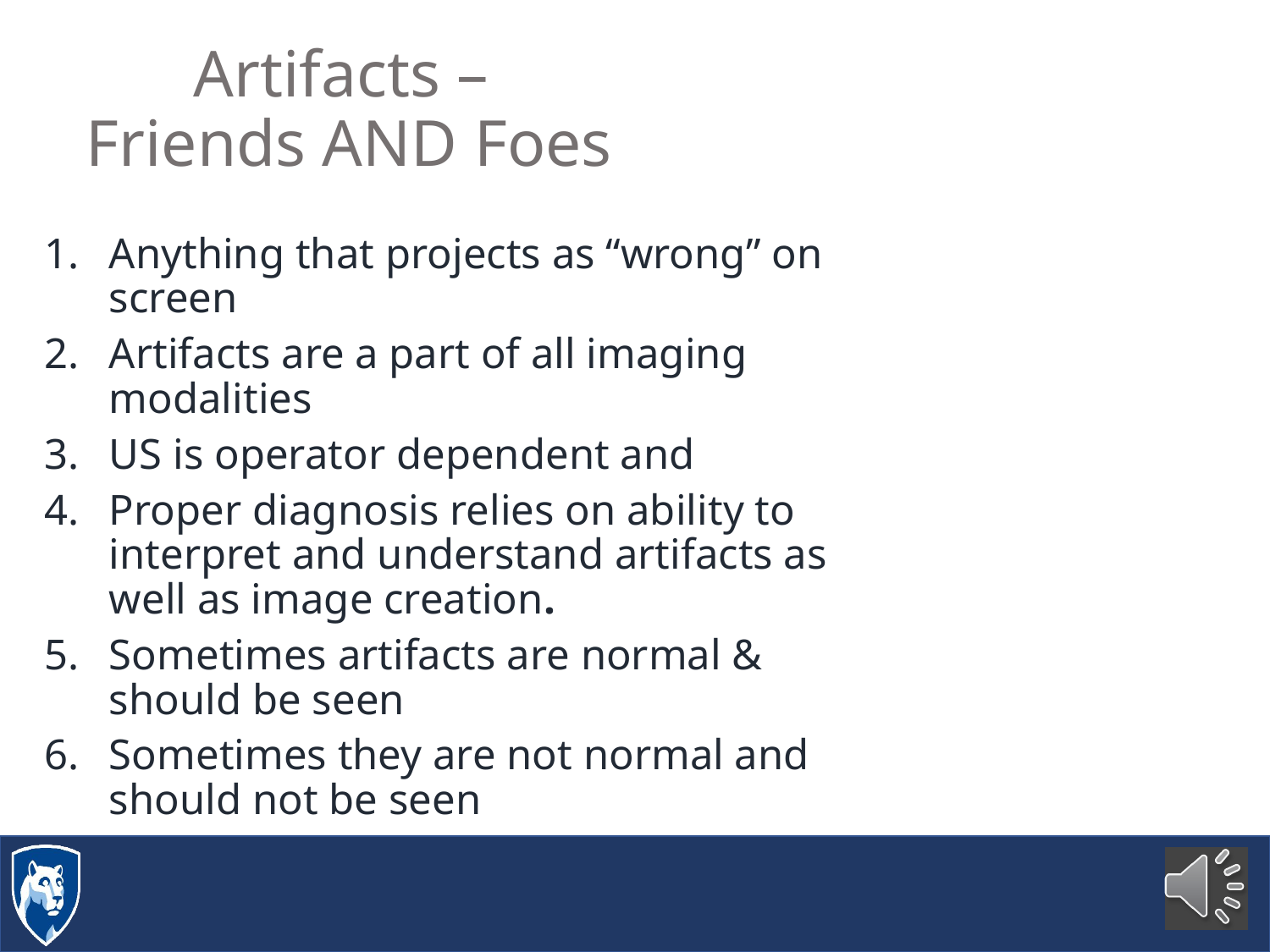

# Artifacts – Friends AND Foes
Anything that projects as “wrong” on screen
Artifacts are a part of all imaging modalities
US is operator dependent and
Proper diagnosis relies on ability to interpret and understand artifacts as well as image creation.
Sometimes artifacts are normal & should be seen
Sometimes they are not normal and should not be seen

## Slide 8
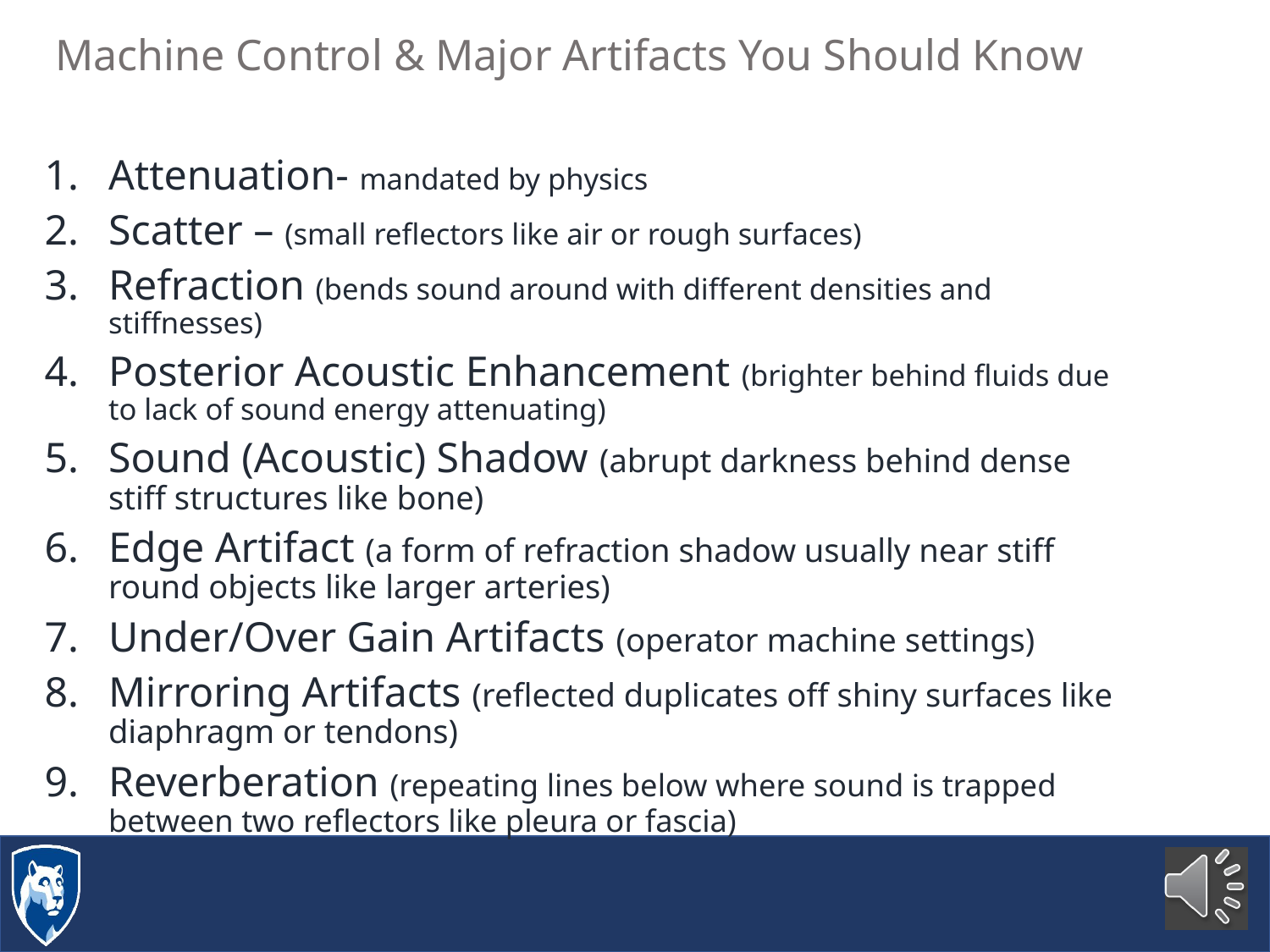

# Machine Control & Major Artifacts You Should Know
Attenuation- mandated by physics
Scatter – (small reflectors like air or rough surfaces)
Refraction (bends sound around with different densities and stiffnesses)
Posterior Acoustic Enhancement (brighter behind fluids due to lack of sound energy attenuating)
Sound (Acoustic) Shadow (abrupt darkness behind dense stiff structures like bone)
Edge Artifact (a form of refraction shadow usually near stiff round objects like larger arteries)
Under/Over Gain Artifacts (operator machine settings)
Mirroring Artifacts (reflected duplicates off shiny surfaces like diaphragm or tendons)
Reverberation (repeating lines below where sound is trapped between two reflectors like pleura or fascia)

## Slide 9
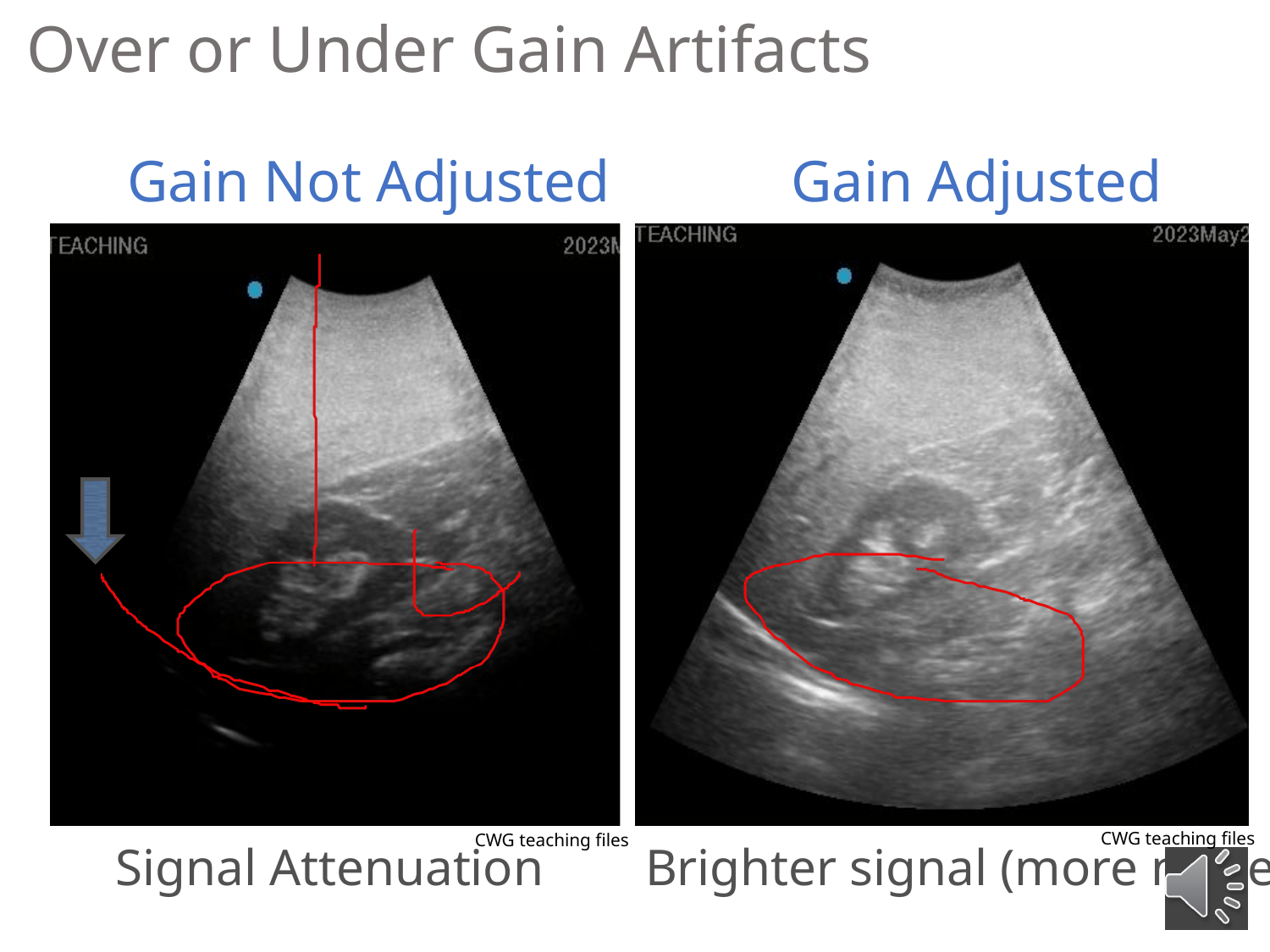

# Over or Under Gain Artifacts
Gain Adjusted
Gain Not Adjusted
CWG teaching files
CWG teaching files
Signal Attenuation
Brighter signal (more noise)

## Slide 10
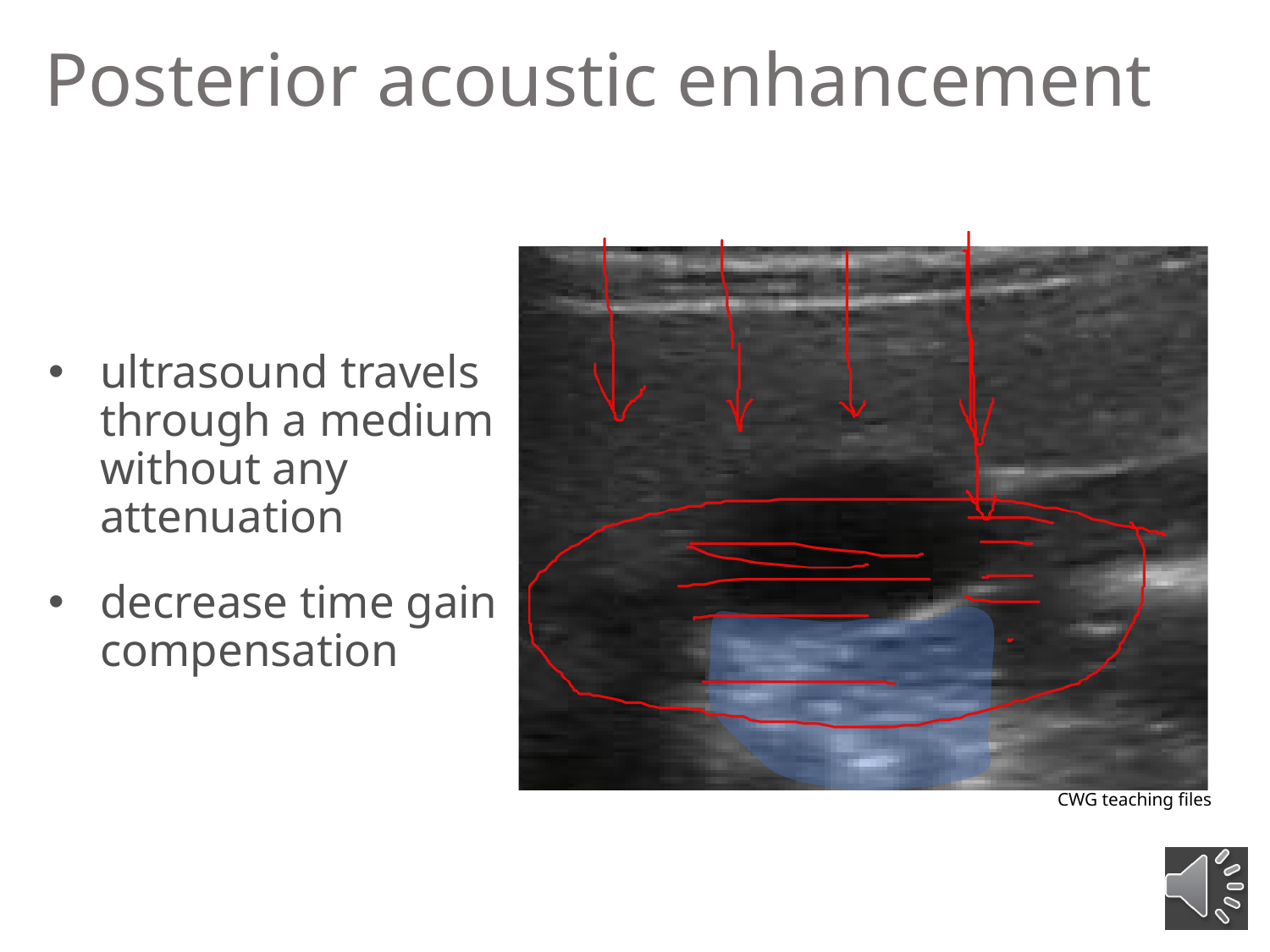

# Posterior acoustic enhancement
ultrasound travels through a medium without any attenuation
decrease time gain compensation
CWG teaching files

## Slide 11
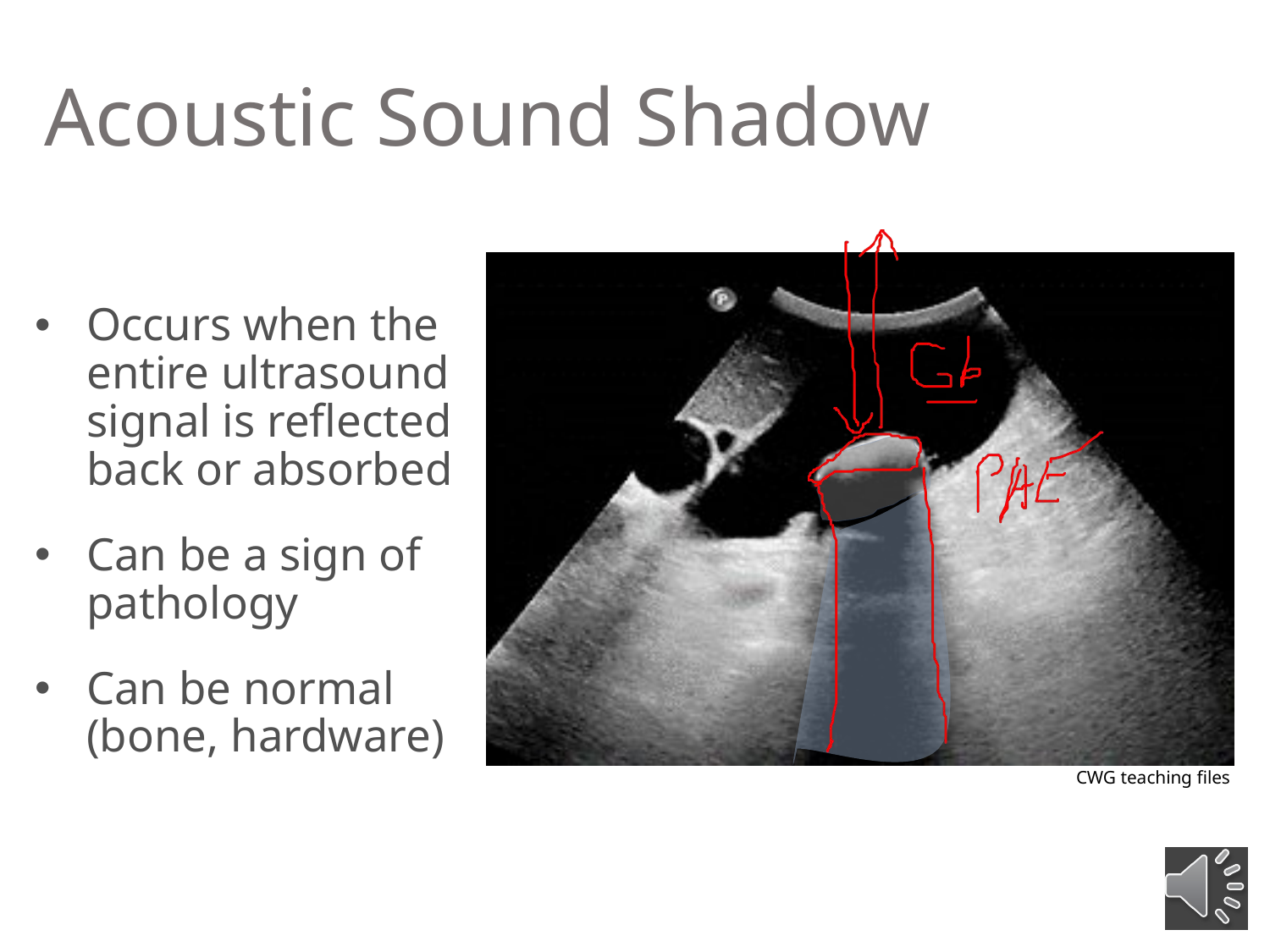

# Acoustic Sound Shadow
Occurs when the entire ultrasound signal is reflected back or absorbed
Can be a sign of pathology
Can be normal (bone, hardware)
CWG teaching files

## Slide 12
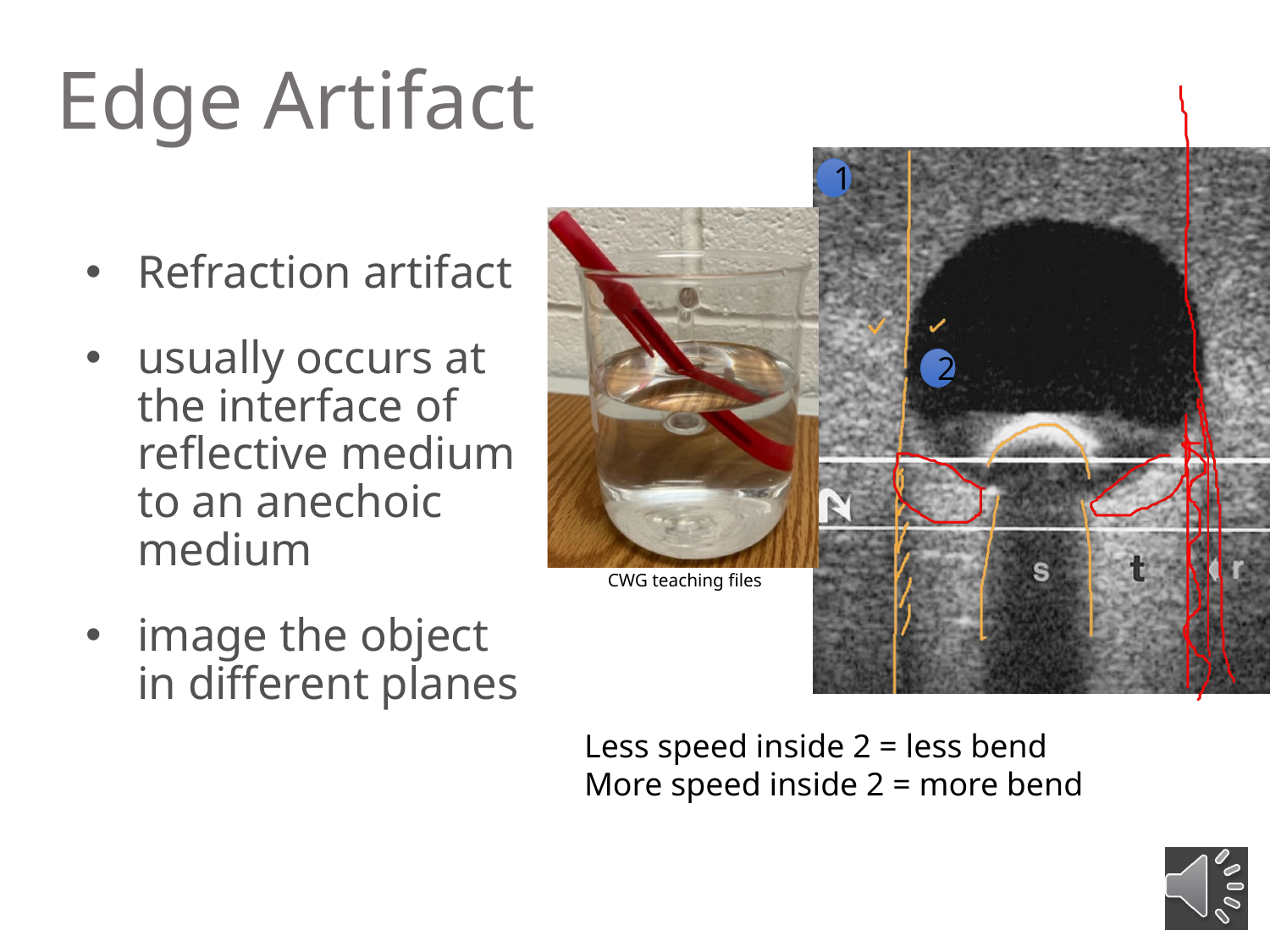

# Edge Artifact
1
Refraction artifact
usually occurs at the interface of reflective medium to an anechoic medium
image the object in different planes
2
CWG teaching files
Less speed inside 2 = less bend
More speed inside 2 = more bend

## Slide 13
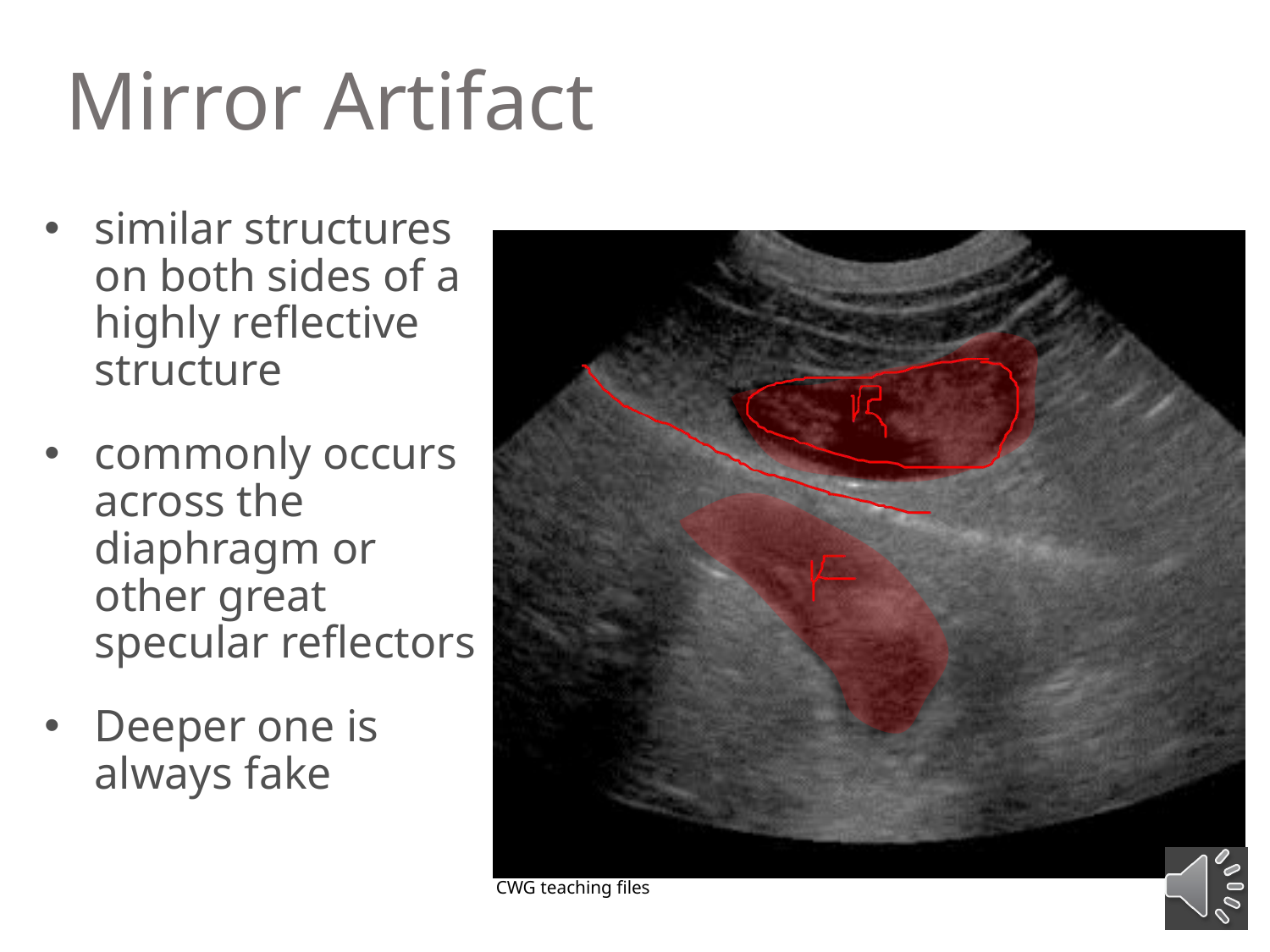

# Mirror Artifact
similar structures on both sides of a highly reflective structure
commonly occurs across the diaphragm or other great specular reflectors
Deeper one is always fake
CWG teaching files

## Slide 14
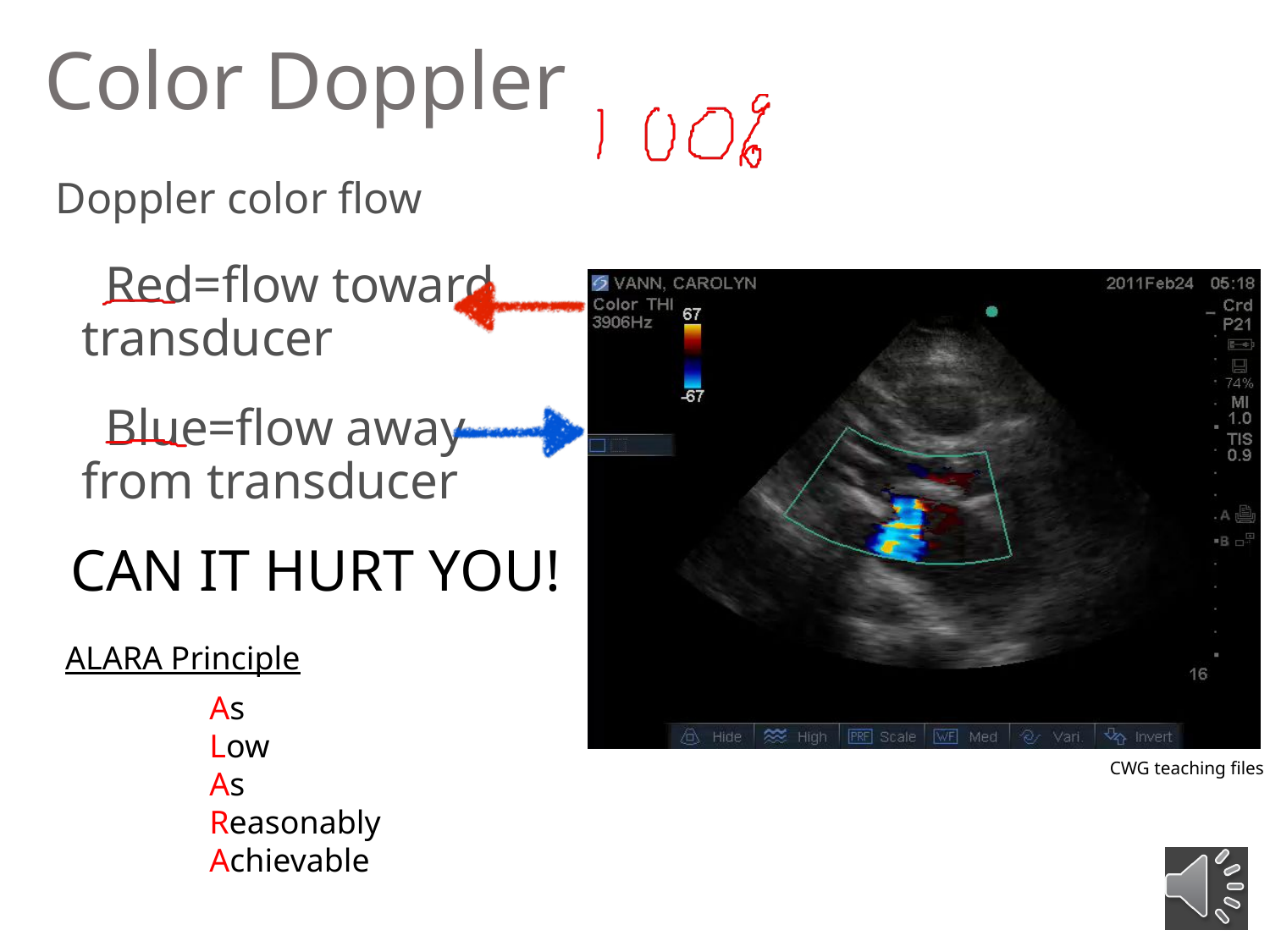

# Color Doppler
Doppler color flow
Red=flow toward transducer
Blue=flow away from transducer
CAN IT HURT YOU!
ALARA Principle
As
Low
As
Reasonably
Achievable
CWG teaching files

## Slide 15
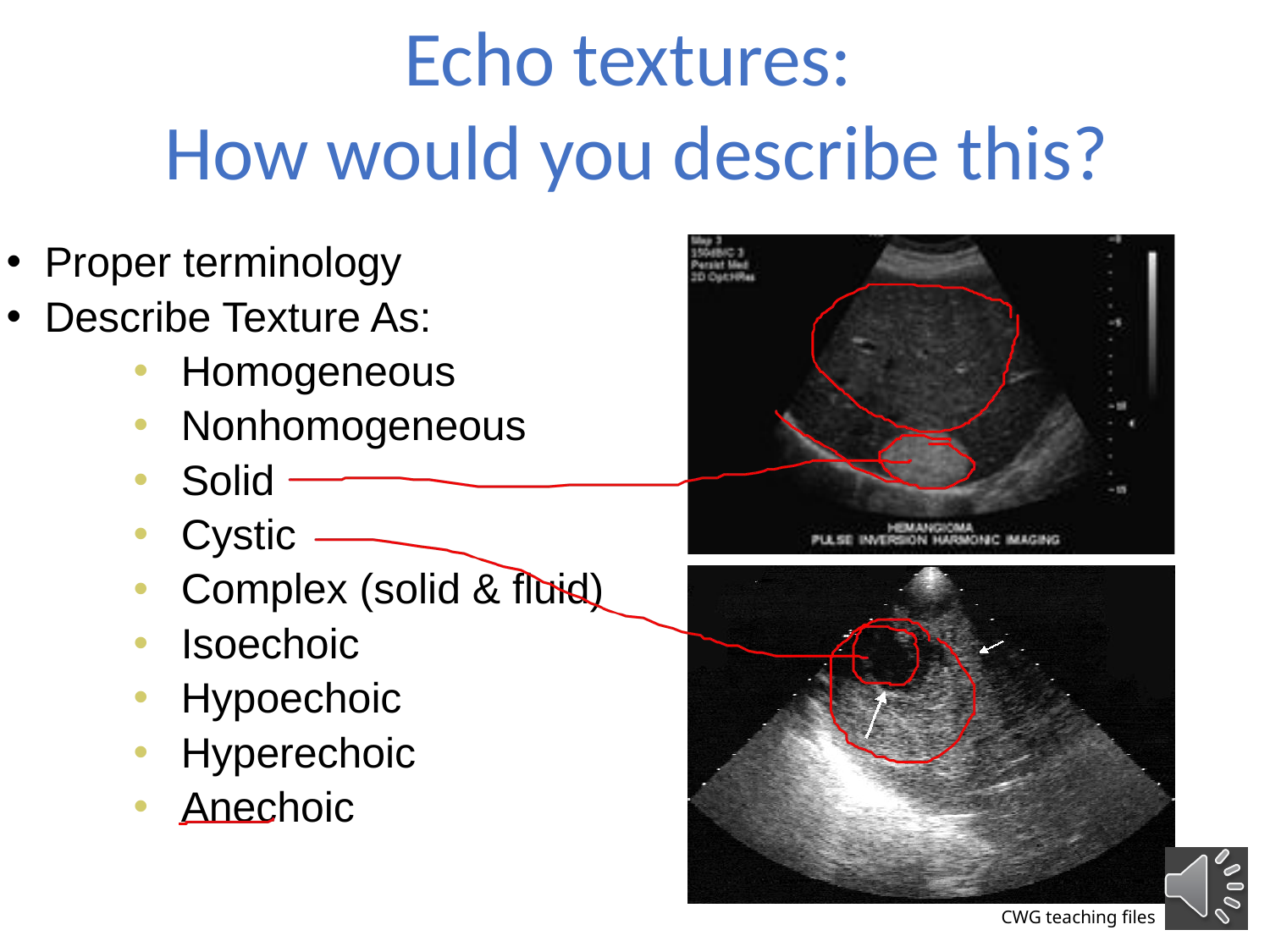

Echo textures:
How would you describe this?
Proper terminology
Describe Texture As:
Homogeneous
Nonhomogeneous
Solid
Cystic
Complex (solid & fluid)
Isoechoic
Hypoechoic
Hyperechoic
Anechoic
CWG teaching files

## Slide 16
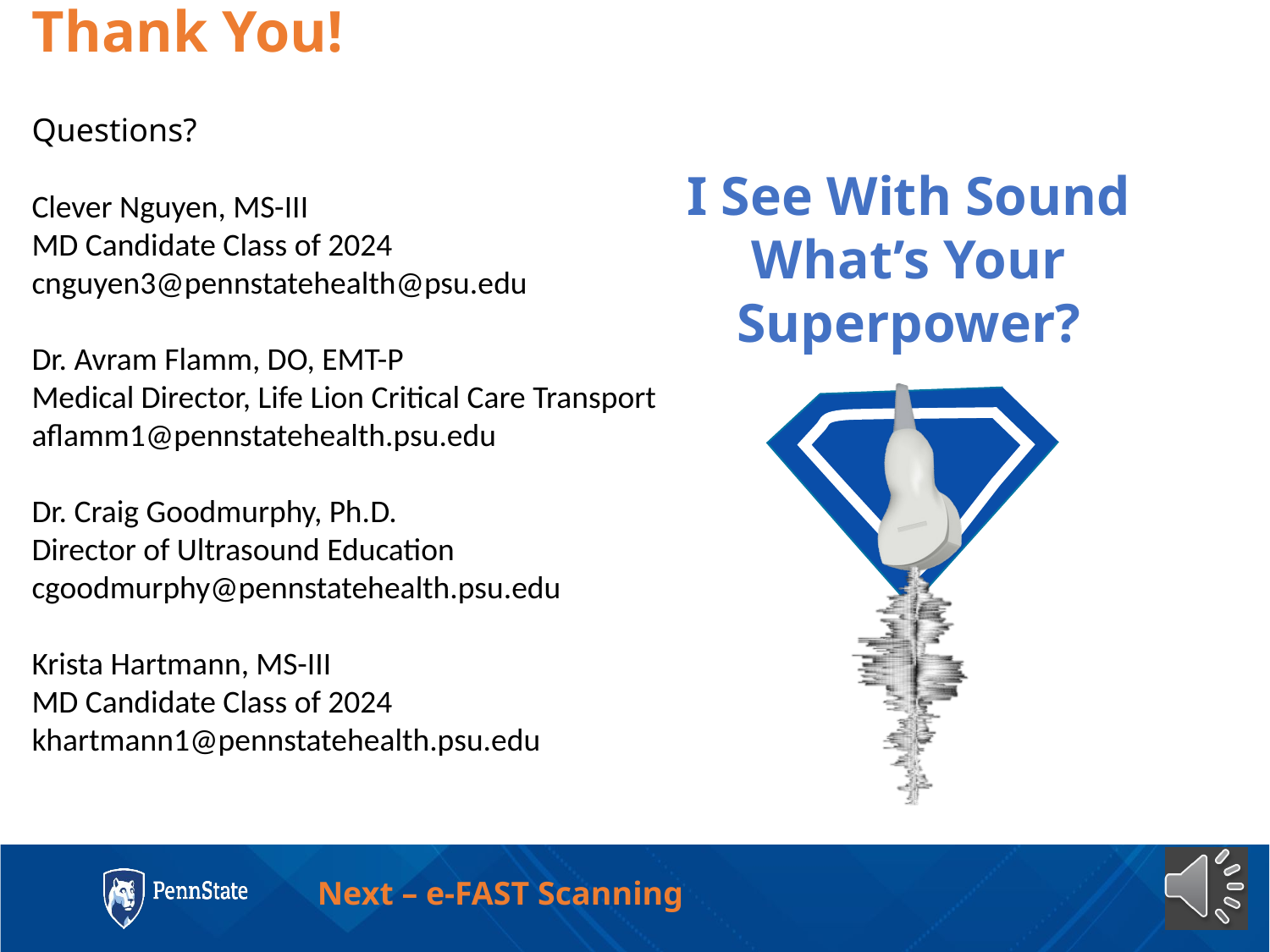

# Thank You!
Questions?
Clever Nguyen, MS-III
MD Candidate Class of 2024
cnguyen3@pennstatehealth@psu.edu
Dr. Avram Flamm, DO, EMT-P
Medical Director, Life Lion Critical Care Transport
aflamm1@pennstatehealth.psu.edu
Dr. Craig Goodmurphy, Ph.D.
Director of Ultrasound Education
cgoodmurphy@pennstatehealth.psu.edu
Krista Hartmann, MS-III
MD Candidate Class of 2024
khartmann1@pennstatehealth.psu.edu
I See With Sound What’s Your Superpower?
Next – e-FAST Scanning
