# Supplementary material for: E-FAST Ultrasound Training Curriculum for Prehospital Emergency Medical Service (EMS) Clinicians
Source: J Educ Teach Emerg Med. 2024 Jan 31;9(1):C41–97. doi: 10.21980/J8S060 (PMC10854885; doi:10.21980/J8S060)
Supplement: Supplementary file 19 — Please see associated Power Point Lecture Link: https://youtu.be/M57N0Gx4sWQ [file jetem-9-1-C41-AppendixL.pptx]

## Slide 1
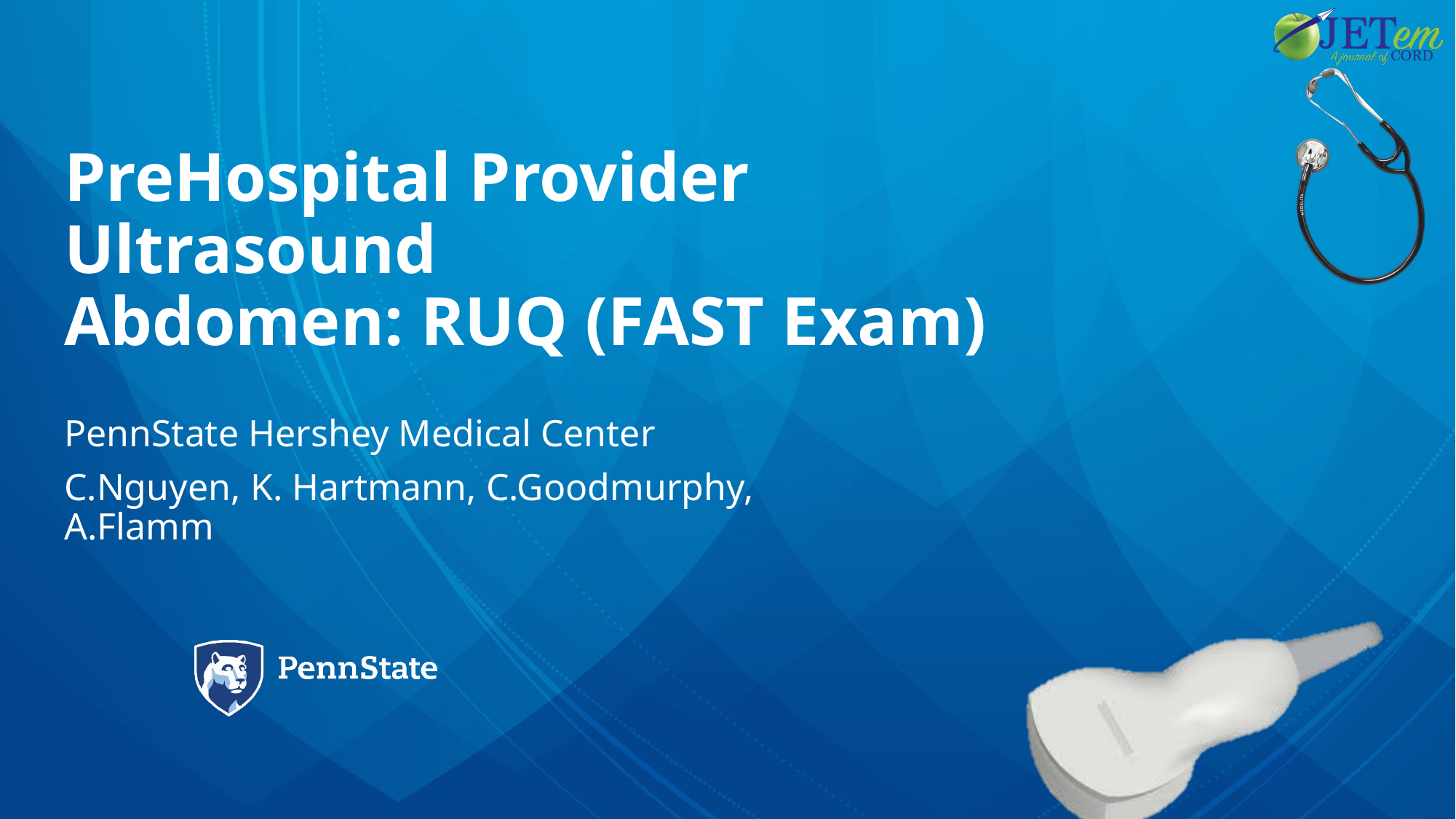

# PreHospital Provider Ultrasound Abdomen: RUQ (FAST Exam)
PennState Hershey Medical Center
C.Nguyen, K. Hartmann, C.Goodmurphy, A.Flamm

## Slide 2
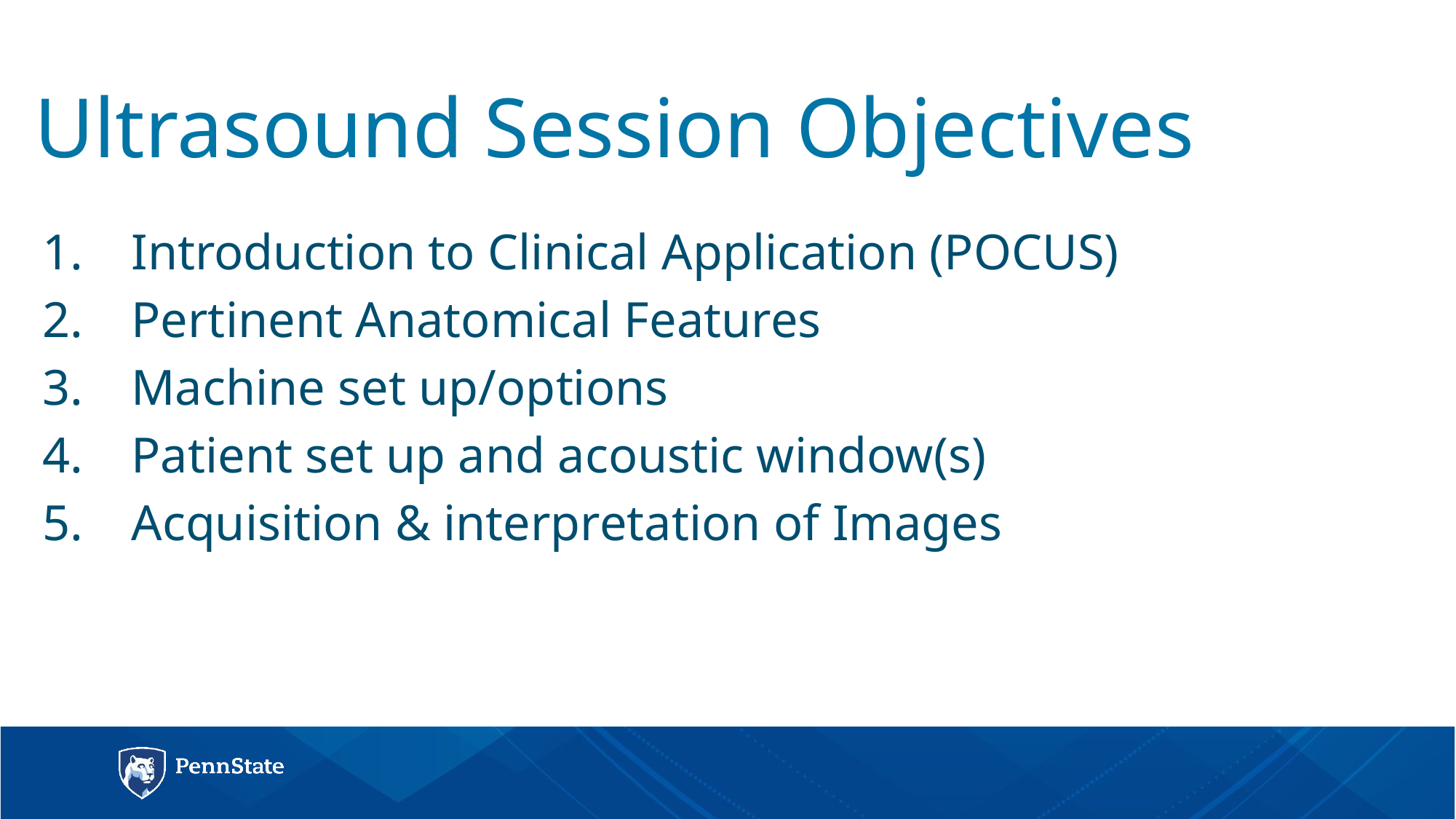

# Ultrasound Session Objectives
Introduction to Clinical Application (POCUS)
Pertinent Anatomical Features
Machine set up/options
Patient set up and acoustic window(s)
Acquisition & interpretation of Images

## Slide 3
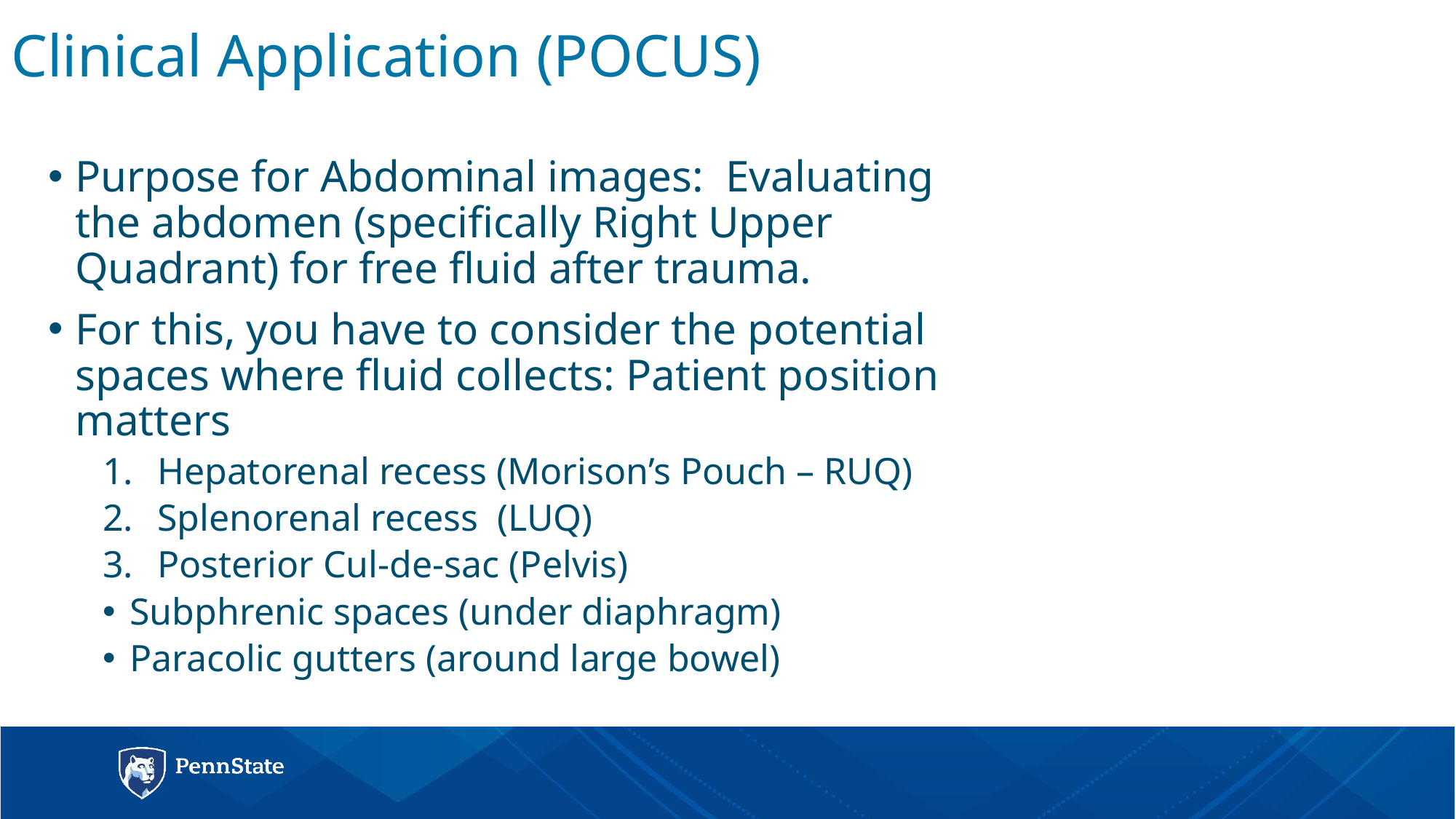

# Clinical Application (POCUS)
Purpose for Abdominal images: Evaluating the abdomen (specifically Right Upper Quadrant) for free fluid after trauma.
For this, you have to consider the potential spaces where fluid collects: Patient position matters
Hepatorenal recess (Morison’s Pouch – RUQ)
Splenorenal recess (LUQ)
Posterior Cul-de-sac (Pelvis)
Subphrenic spaces (under diaphragm)
Paracolic gutters (around large bowel)

## Slide 4
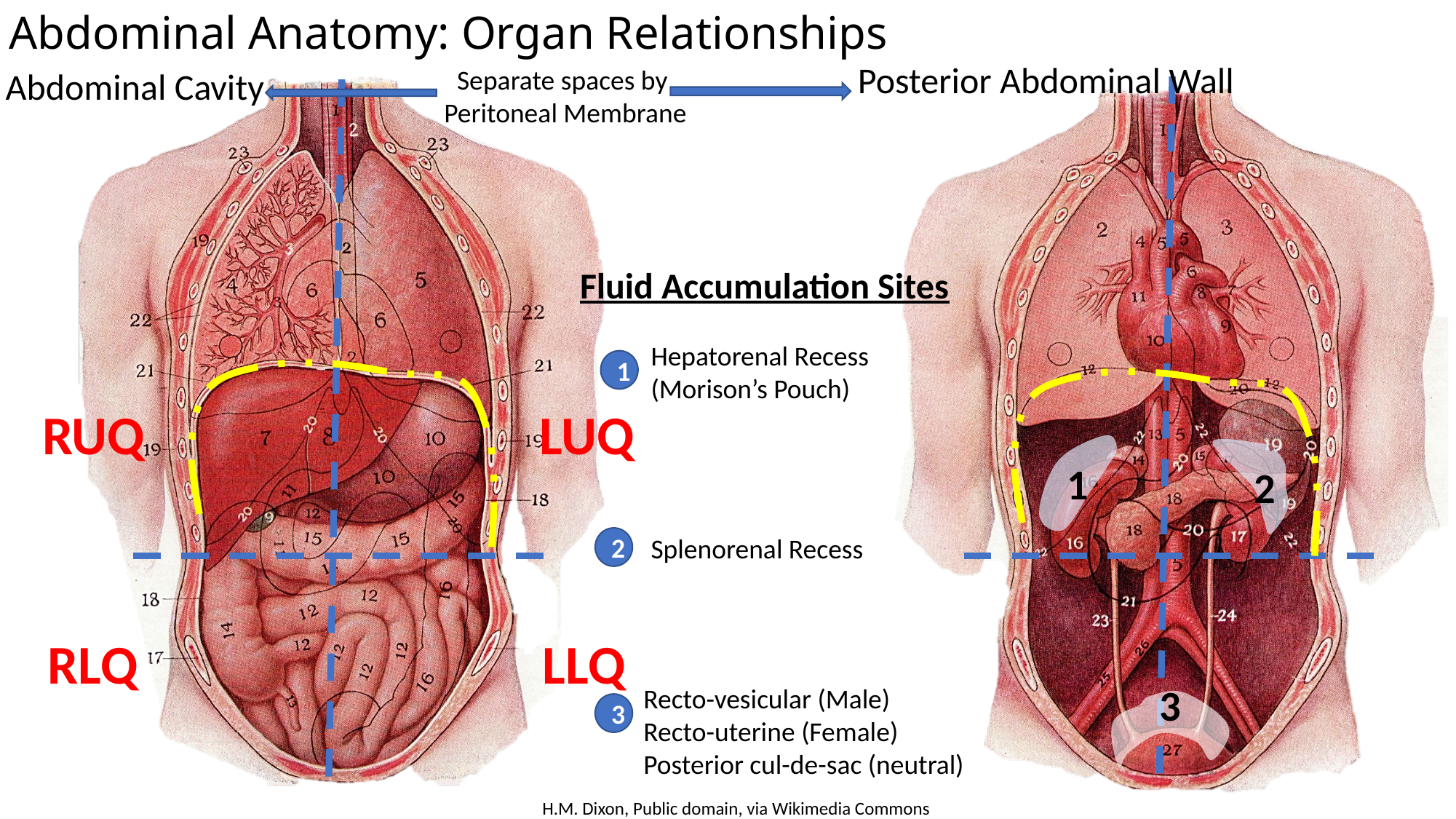

Abdominal Anatomy: Organ Relationships
Posterior Abdominal Wall
Separate spaces by
Peritoneal Membrane
Abdominal Cavity
Fluid Accumulation Sites
Hepatorenal Recess
(Morison’s Pouch)
1
RUQ
LUQ
1
 2
Splenorenal Recess
2
RLQ
LLQ
Recto-vesicular (Male)
Recto-uterine (Female)
Posterior cul-de-sac (neutral)
3
3
H.M. Dixon, Public domain, via Wikimedia Commons

## Slide 5
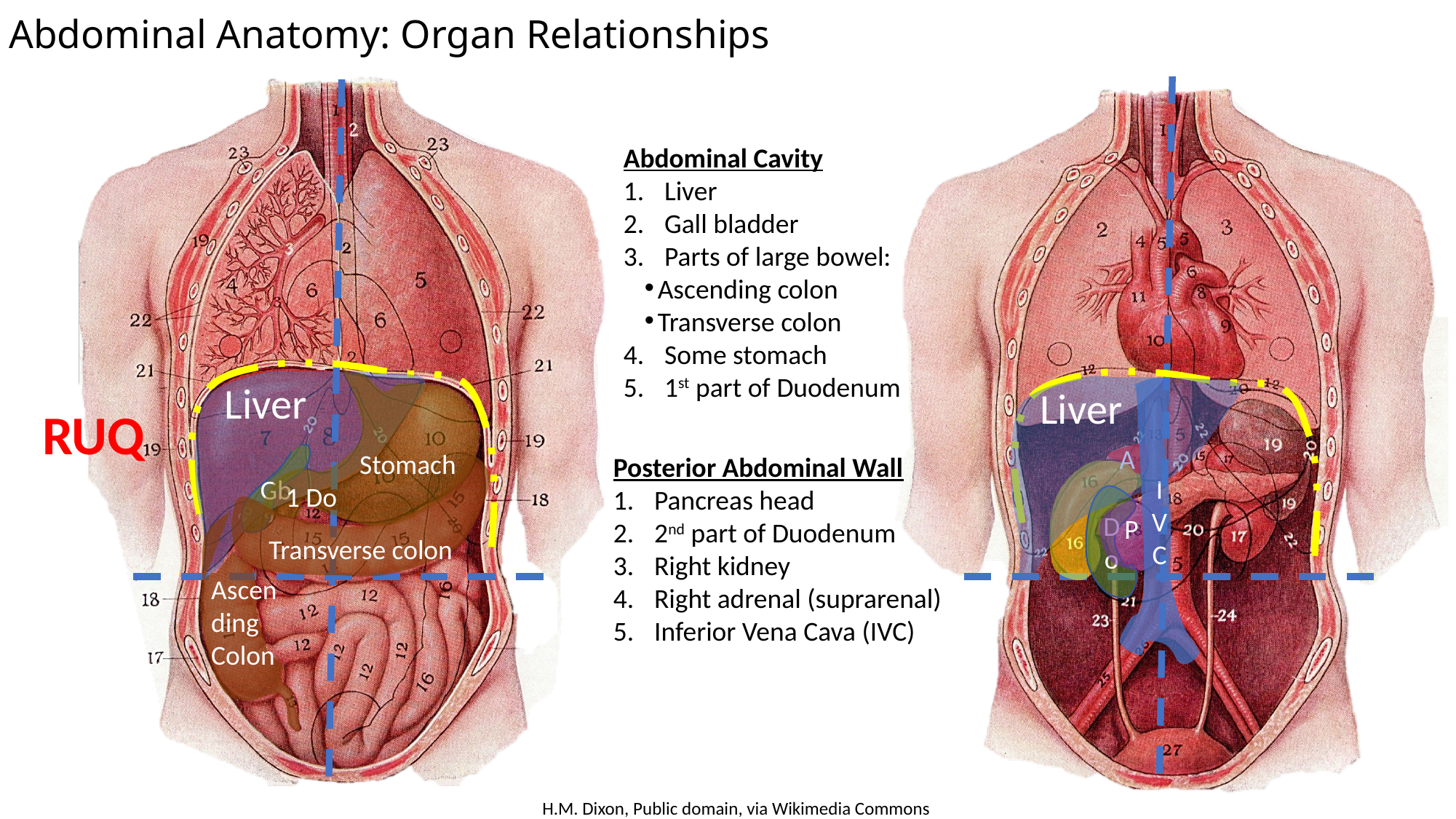

Abdominal Anatomy: Organ Relationships
Abdominal Cavity
Liver
Gall bladder
Parts of large bowel:
Ascending colon
Transverse colon
Some stomach
1st part of Duodenum
 Stomach
 1 Do
 Liver
 Liver
I
V
C
RUQ
A
Gb
Posterior Abdominal Wall
Pancreas head
2nd part of Duodenum
Right kidney
Right adrenal (suprarenal)
Inferior Vena Cava (IVC)
Transverse colon
P
Do
Ascending
Colon
H.M. Dixon, Public domain, via Wikimedia Commons

## Slide 6
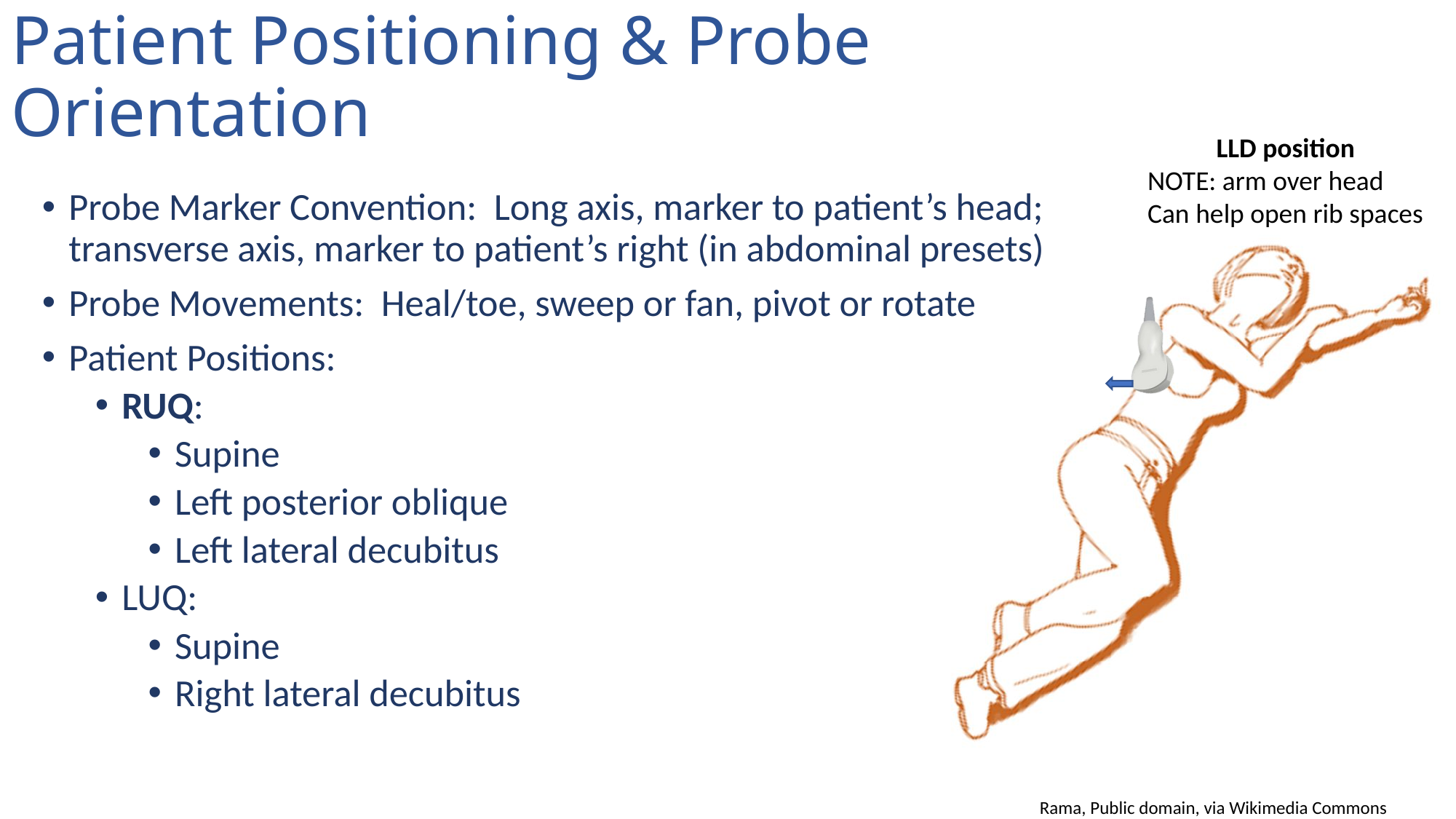

Patient Positioning & Probe Orientation
LLD position
NOTE: arm over head
Can help open rib spaces
Probe Marker Convention: Long axis, marker to patient’s head; transverse axis, marker to patient’s right (in abdominal presets)
Probe Movements: Heal/toe, sweep or fan, pivot or rotate
Patient Positions:
RUQ:
Supine
Left posterior oblique
Left lateral decubitus
LUQ:
Supine
Right lateral decubitus
Rama, Public domain, via Wikimedia Commons

## Slide 7
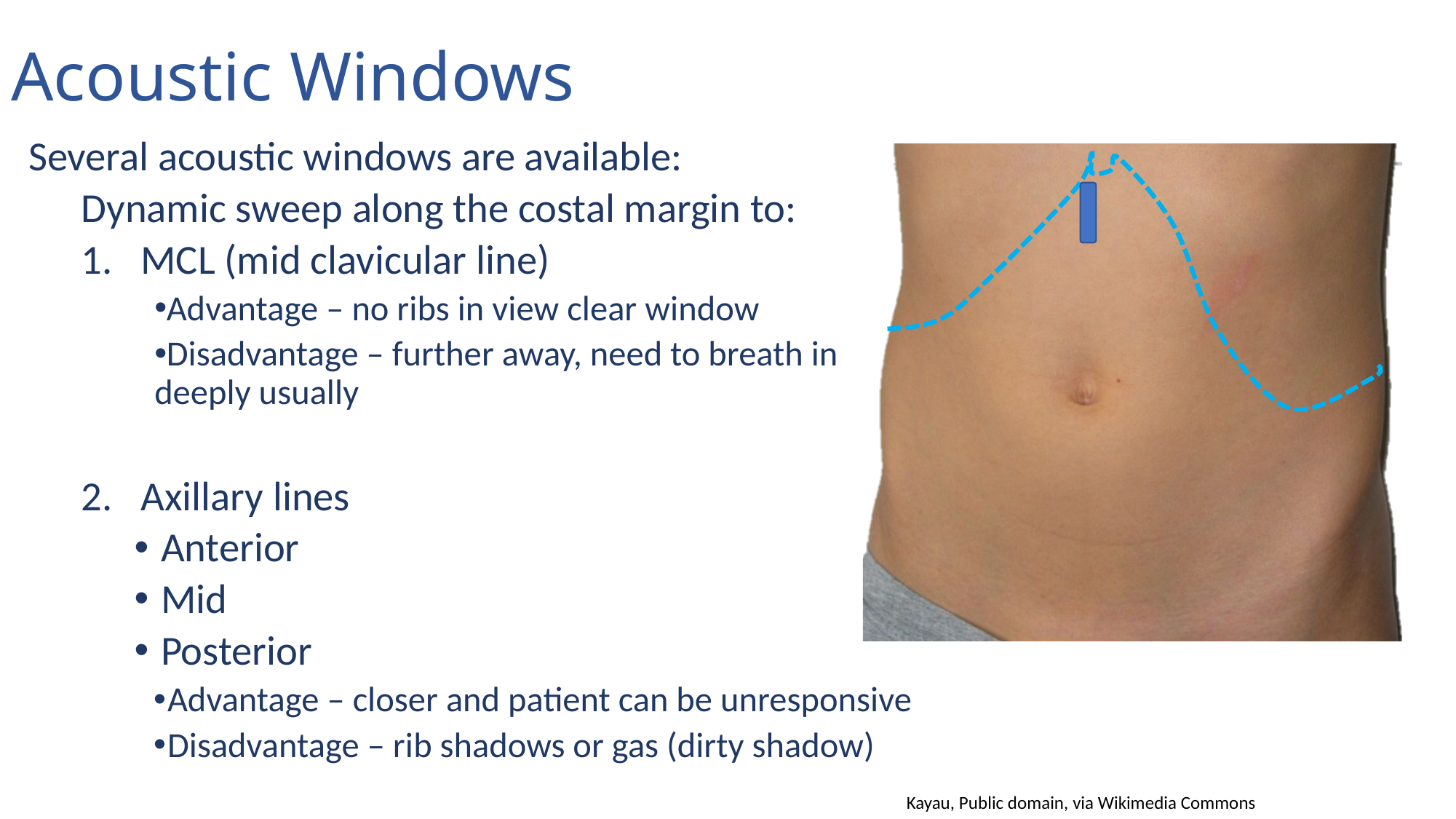

Acoustic Windows
Several acoustic windows are available:
Dynamic sweep along the costal margin to:
MCL (mid clavicular line)
Advantage – no ribs in view clear window
Disadvantage – further away, need to breath in deeply usually
Axillary lines
Anterior
Mid
Posterior
Advantage – closer and patient can be unresponsive
Disadvantage – rib shadows or gas (dirty shadow)
Kayau, Public domain, via Wikimedia Commons

## Slide 8
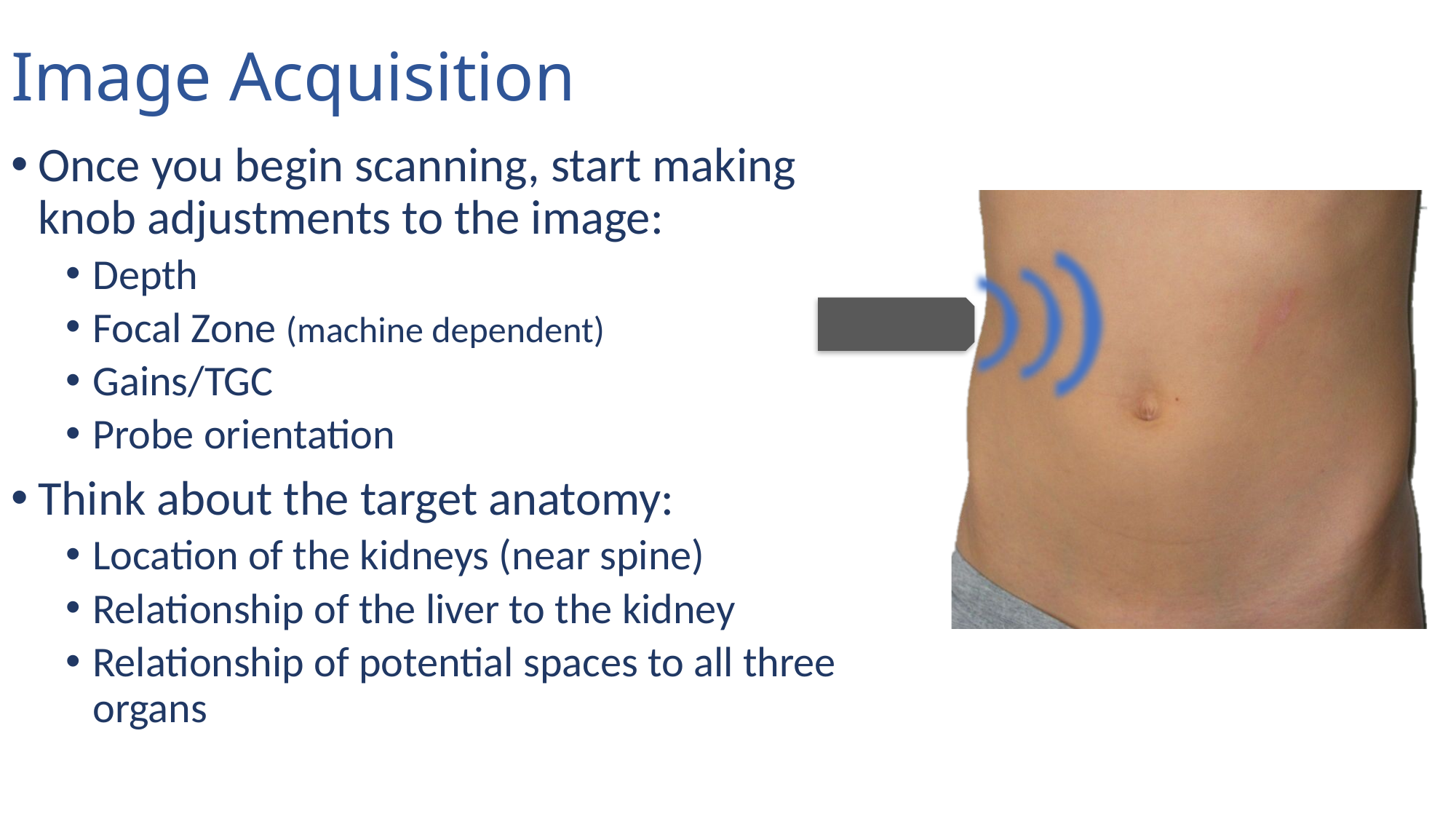

Image Acquisition
Once you begin scanning, start making knob adjustments to the image:
Depth
Focal Zone (machine dependent)
Gains/TGC
Probe orientation
Think about the target anatomy:
Location of the kidneys (near spine)
Relationship of the liver to the kidney
Relationship of potential spaces to all three organs

## Slide 9
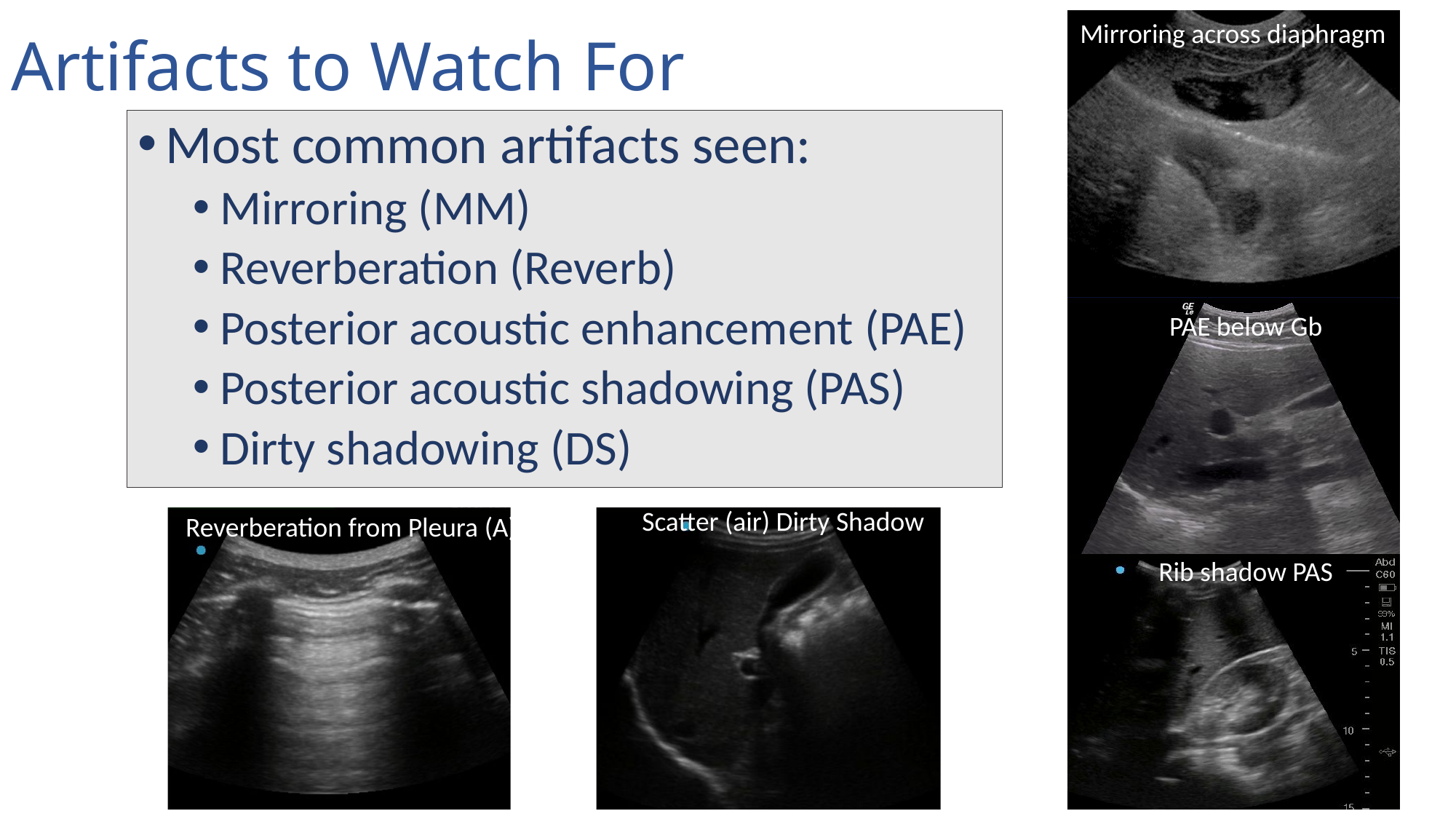

Artifacts to Watch For
Mirroring across diaphragm
Most common artifacts seen:
Mirroring (MM)
Reverberation (Reverb)
Posterior acoustic enhancement (PAE)
Posterior acoustic shadowing (PAS)
Dirty shadowing (DS)
PAE below Gb
Scatter (air) Dirty Shadow
Reverberation from Pleura (A)
Rib shadow PAS

## Slide 10
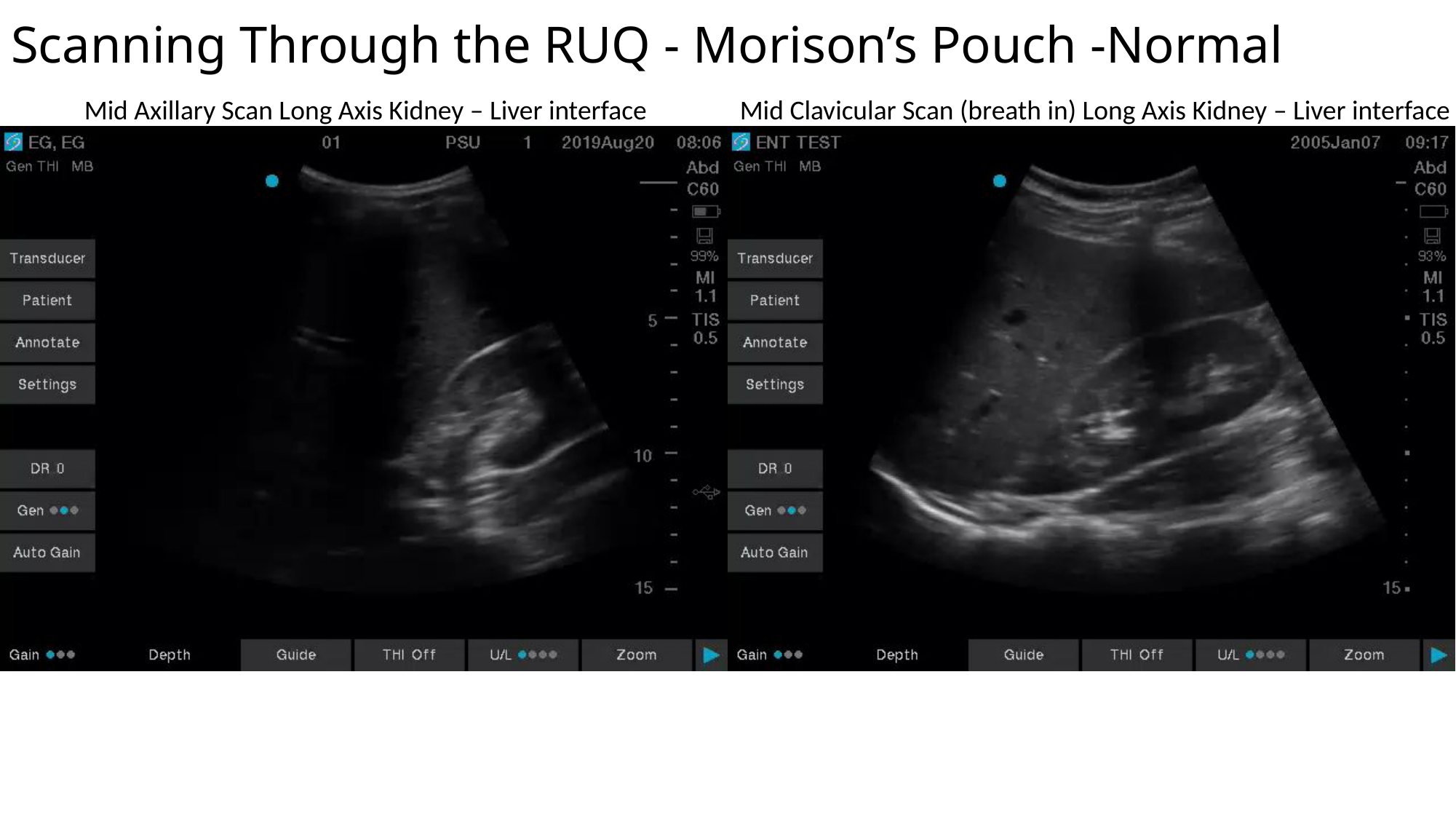

# Scanning Through the RUQ - Morison’s Pouch -Normal
Mid Axillary Scan Long Axis Kidney – Liver interface
Mid Clavicular Scan (breath in) Long Axis Kidney – Liver interface

## Slide 11
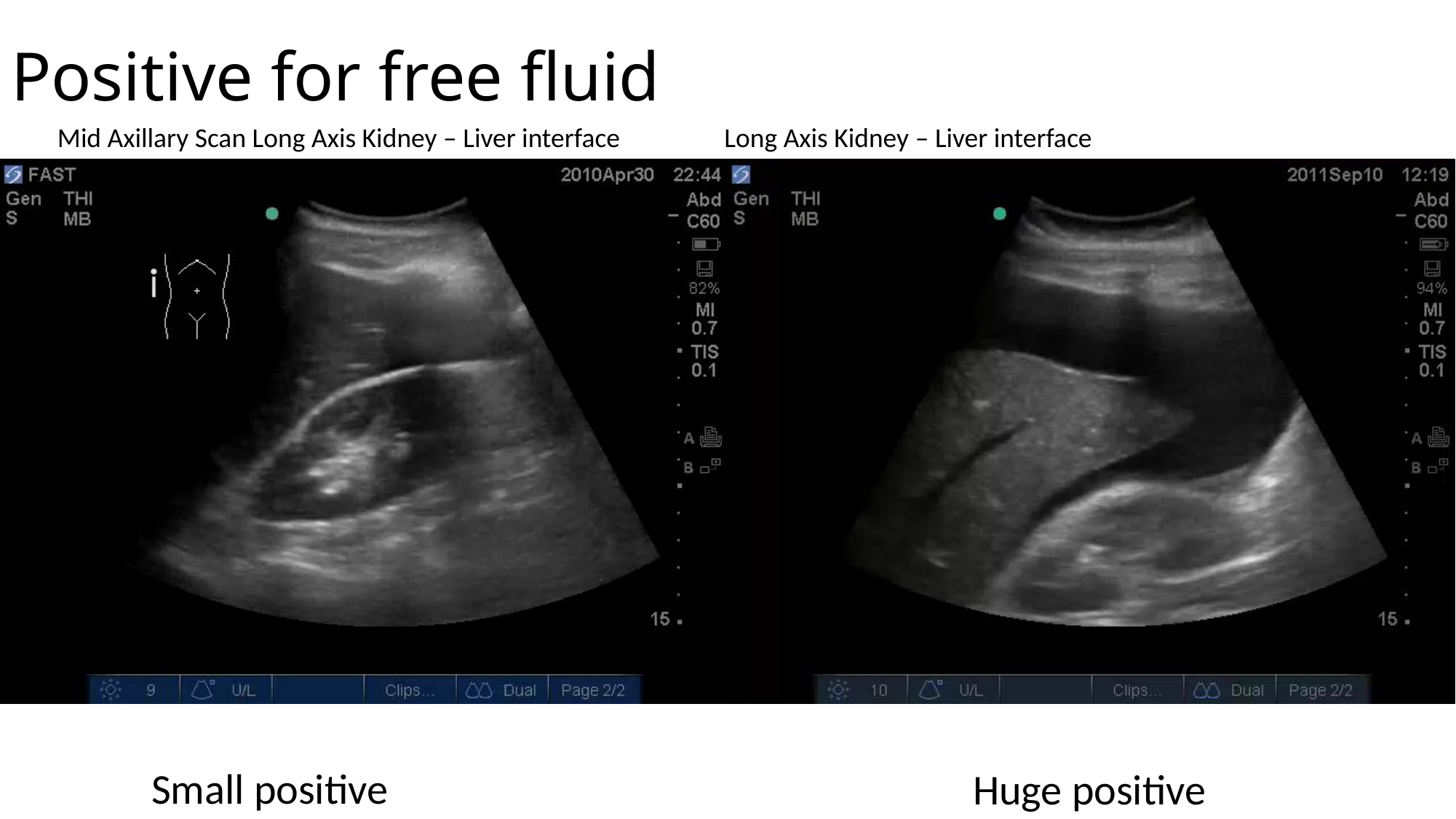

# Positive for free fluid
Mid Axillary Scan Long Axis Kidney – Liver interface
 Long Axis Kidney – Liver interface
Small positive
Huge positive

## Slide 12
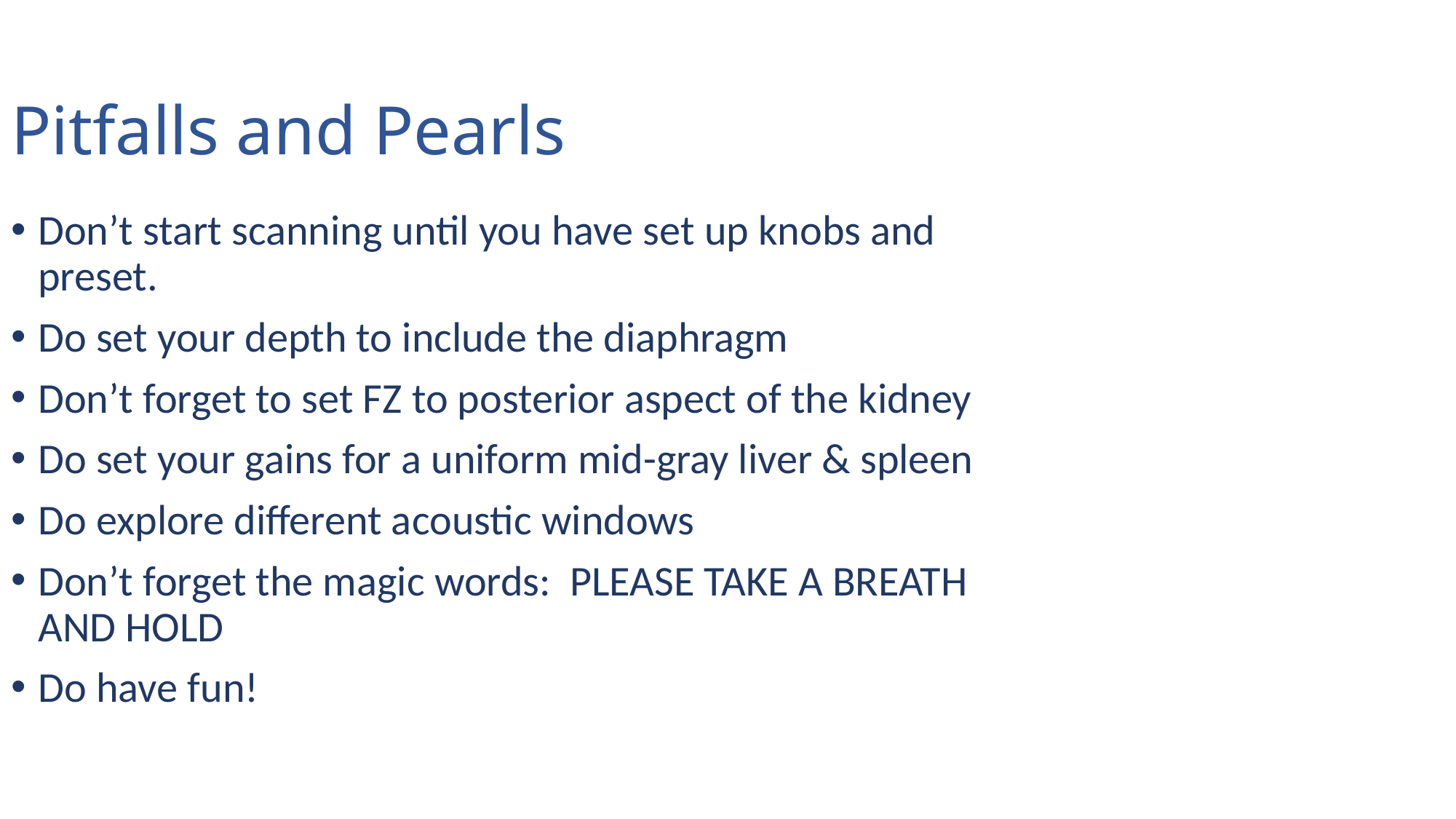

Pitfalls and Pearls
Don’t start scanning until you have set up knobs and preset.
Do set your depth to include the diaphragm
Don’t forget to set FZ to posterior aspect of the kidney
Do set your gains for a uniform mid-gray liver & spleen
Do explore different acoustic windows
Don’t forget the magic words: PLEASE TAKE A BREATH AND HOLD
Do have fun!

## Slide 13
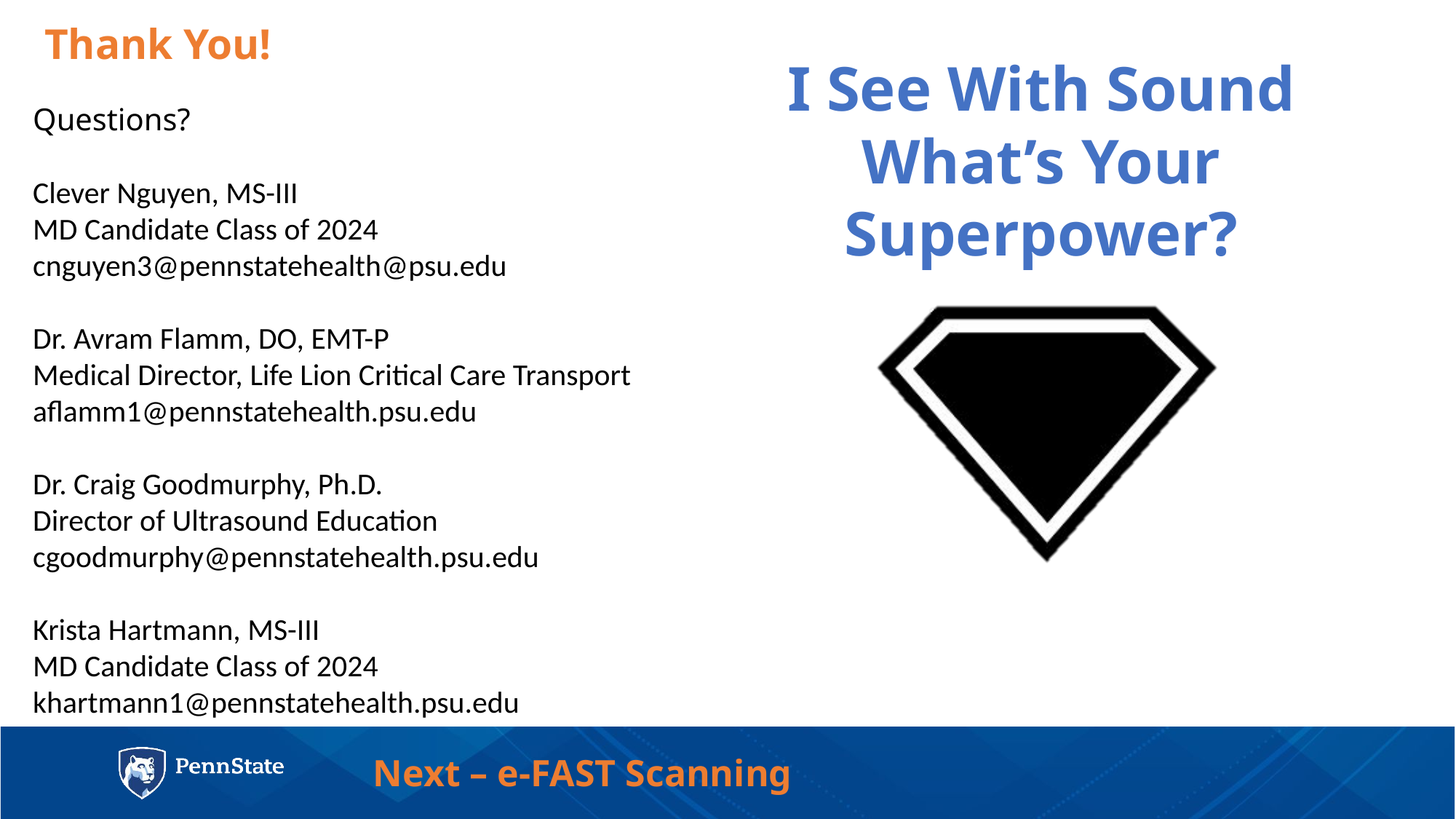

# Thank You!
I See With Sound What’s Your Superpower?
Questions?
Clever Nguyen, MS-III
MD Candidate Class of 2024
cnguyen3@pennstatehealth@psu.edu
Dr. Avram Flamm, DO, EMT-P
Medical Director, Life Lion Critical Care Transport
aflamm1@pennstatehealth.psu.edu
Dr. Craig Goodmurphy, Ph.D.
Director of Ultrasound Education
cgoodmurphy@pennstatehealth.psu.edu
Krista Hartmann, MS-III
MD Candidate Class of 2024
khartmann1@pennstatehealth.psu.edu
Next – e-FAST Scanning
