# Supplementary material for: E-FAST Ultrasound Training Curriculum for Prehospital Emergency Medical Service (EMS) Clinicians
Source: J Educ Teach Emerg Med. 2024 Jan 31;9(1):C41–97. doi: 10.21980/J8S060 (PMC10854885; doi:10.21980/J8S060)
Supplement: Supplementary file 23 — Please see associated Power Point Lecture Link: https://youtu.be/rVHFzFKGVV0 [file jetem-9-1-C41-AppendixP.pptx]

## Slide 1
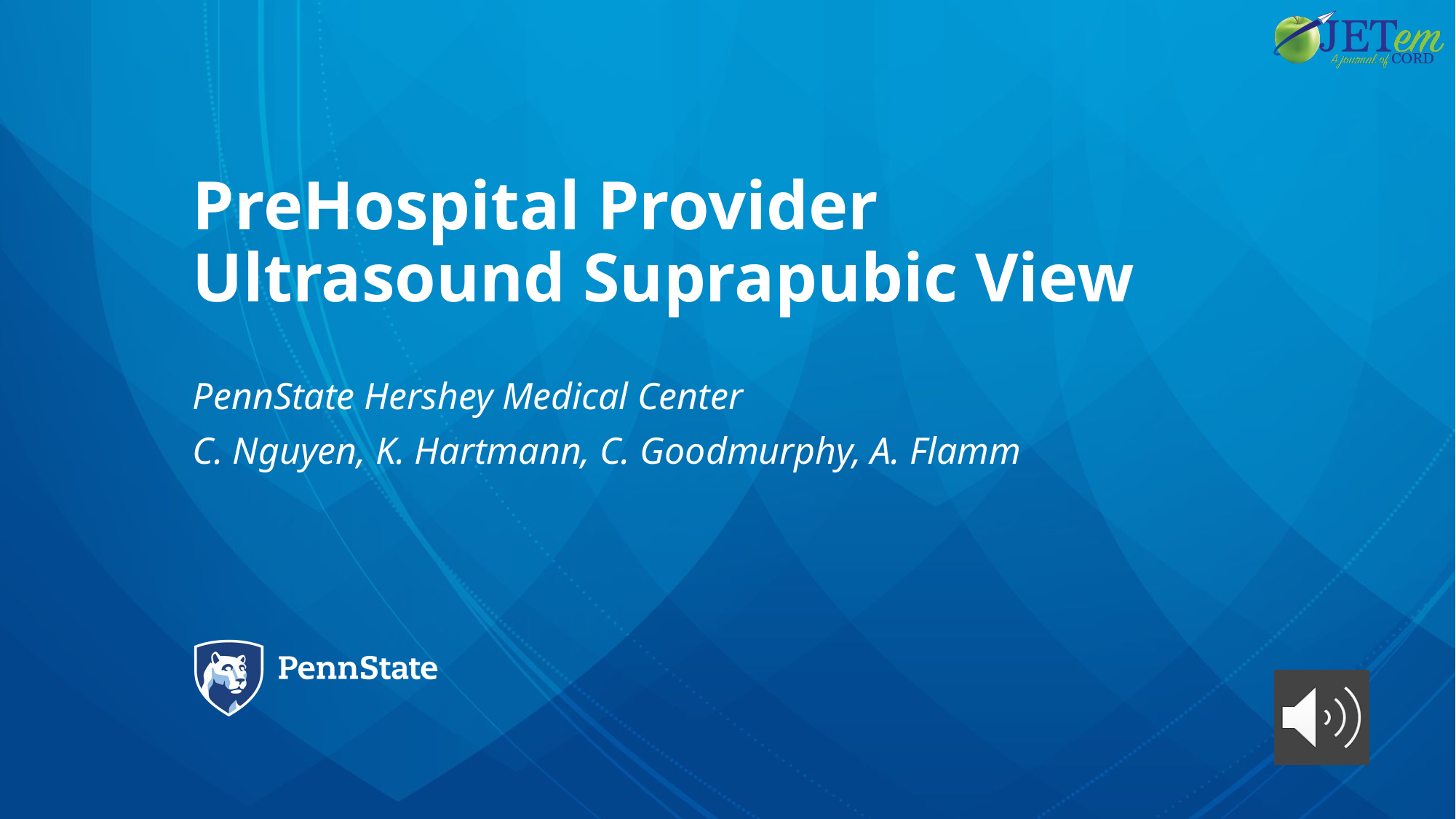

PreHospital Provider Ultrasound Suprapubic View
PennState Hershey Medical Center
C. Nguyen, K. Hartmann, C. Goodmurphy, A. Flamm

## Slide 2
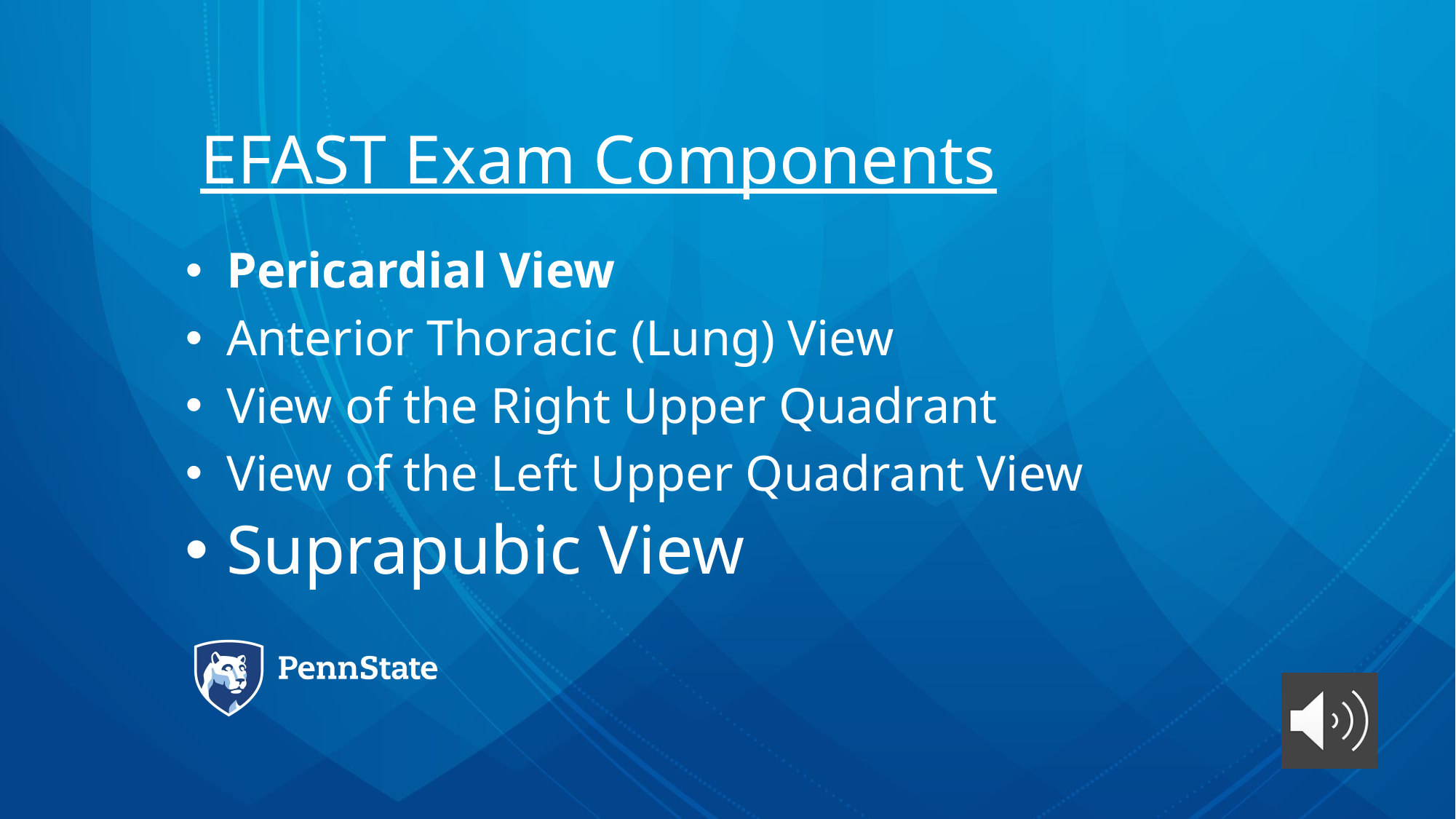

# EFAST Exam Components
Pericardial View
Anterior Thoracic (Lung) View
View of the Right Upper Quadrant
View of the Left Upper Quadrant View
Suprapubic View

## Slide 3
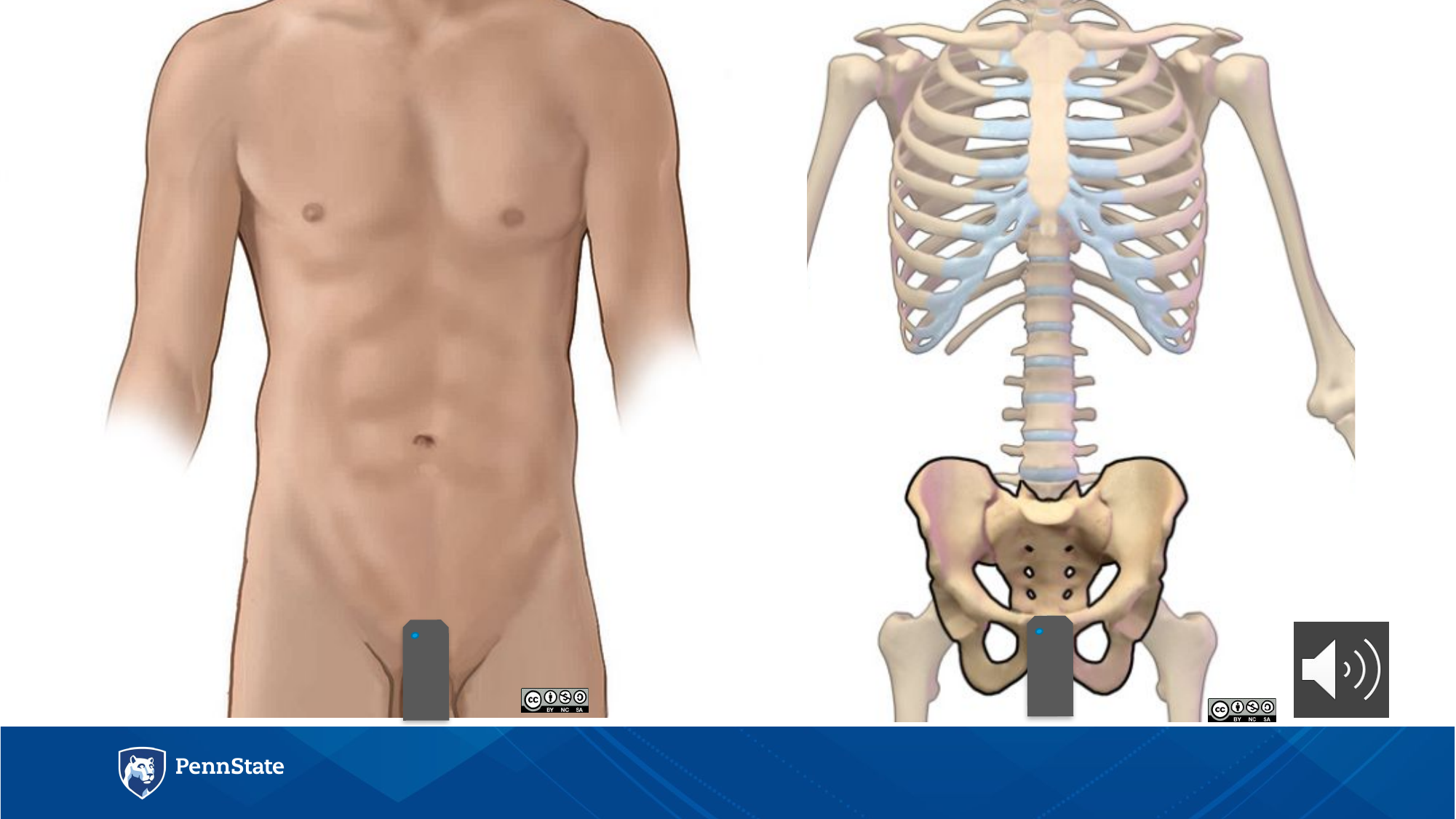

## Slide 4
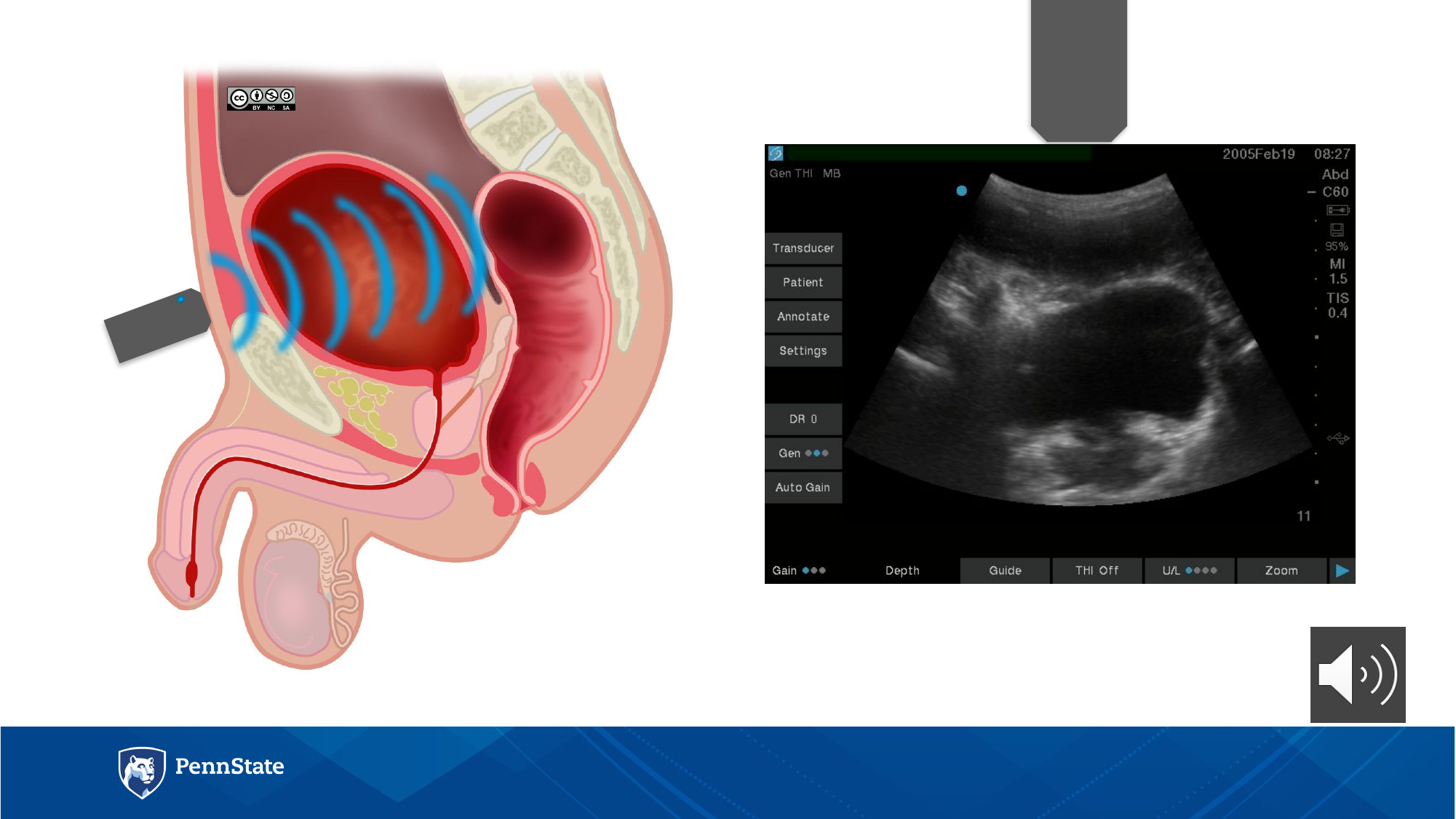

## Slide 5
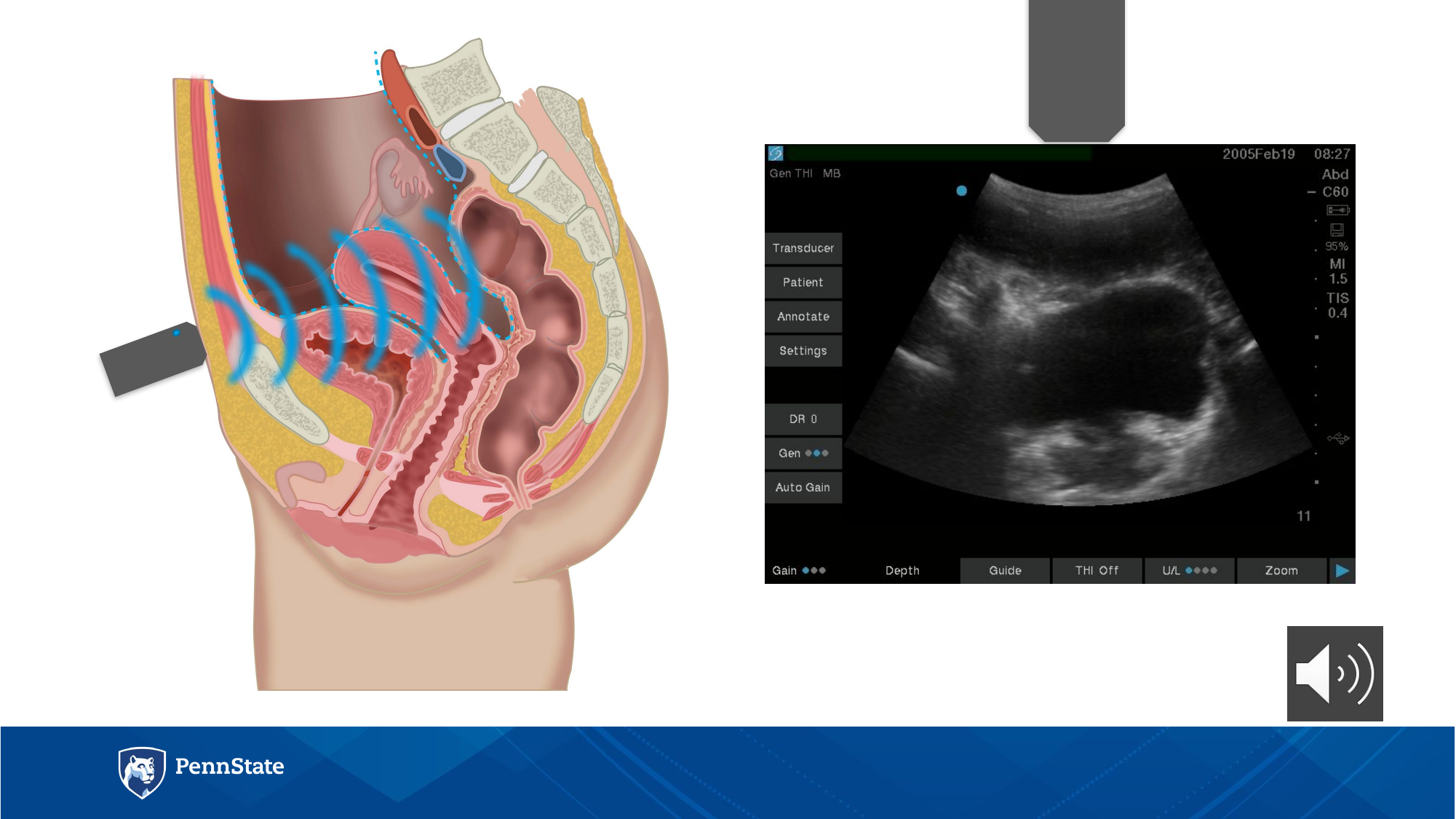

## Slide 6
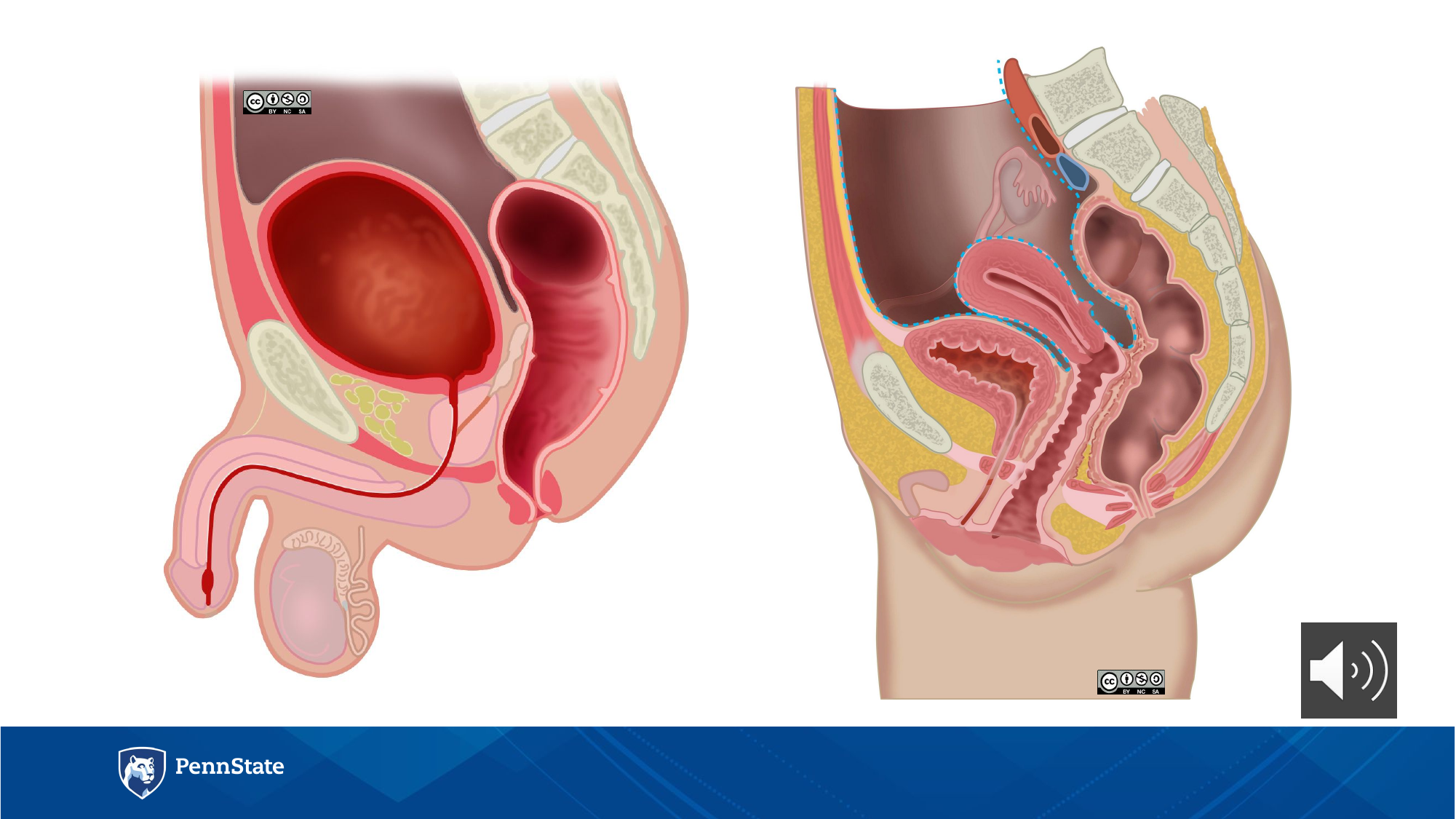

## Slide 7
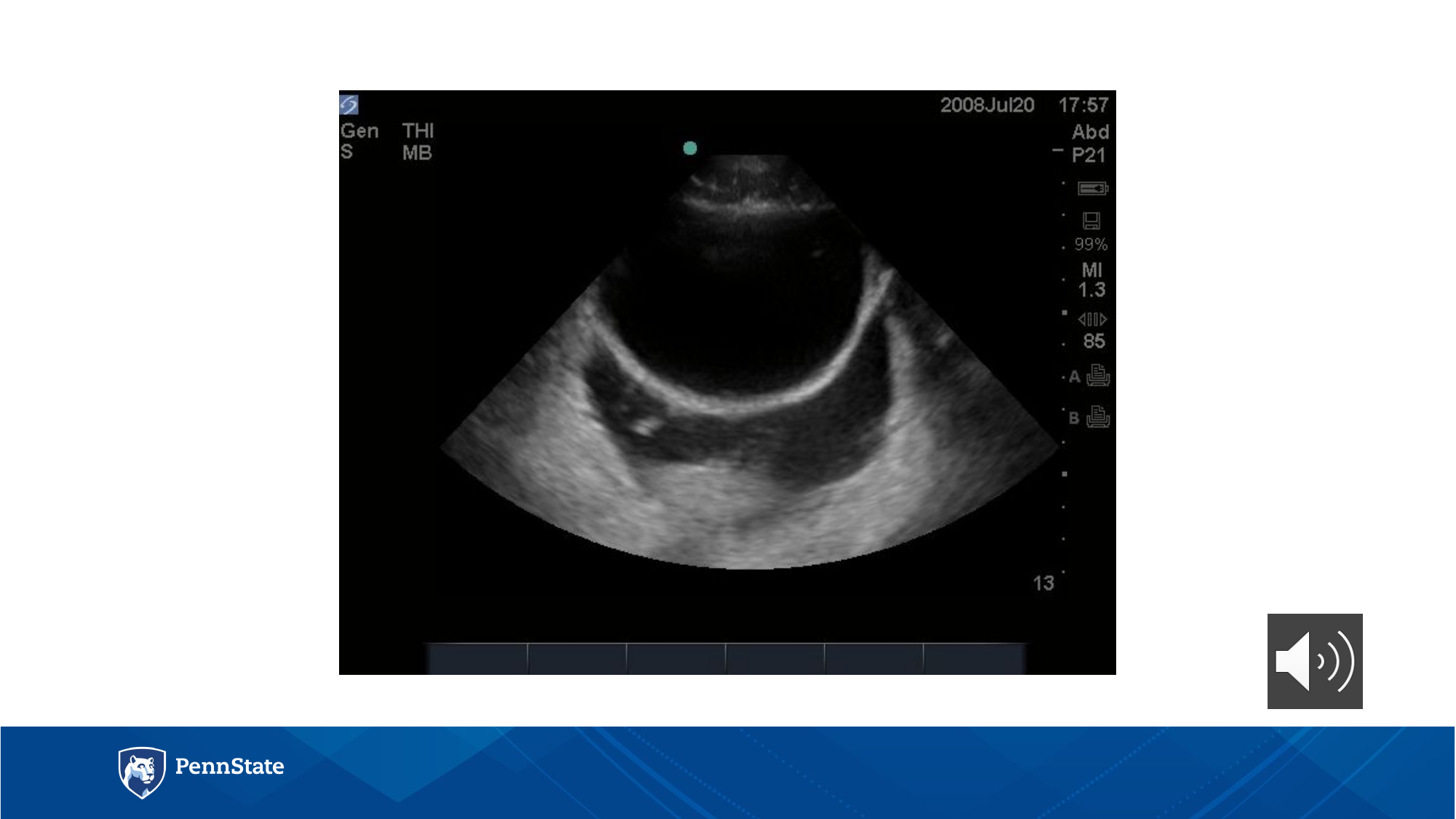

## Slide 8
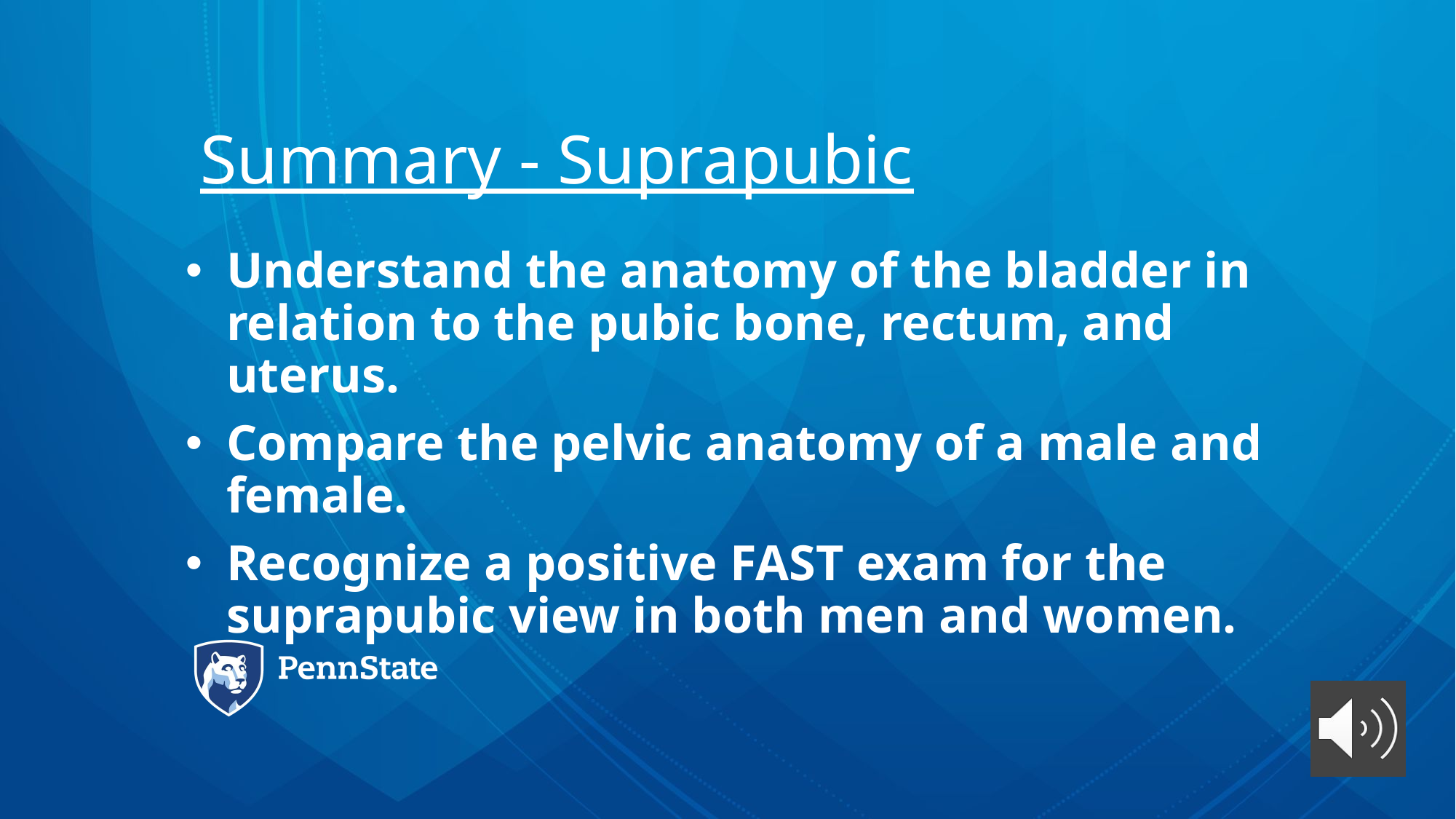

# Summary - Suprapubic
Understand the anatomy of the bladder in relation to the pubic bone, rectum, and uterus.
Compare the pelvic anatomy of a male and female.
Recognize a positive FAST exam for the suprapubic view in both men and women.

## Slide 9
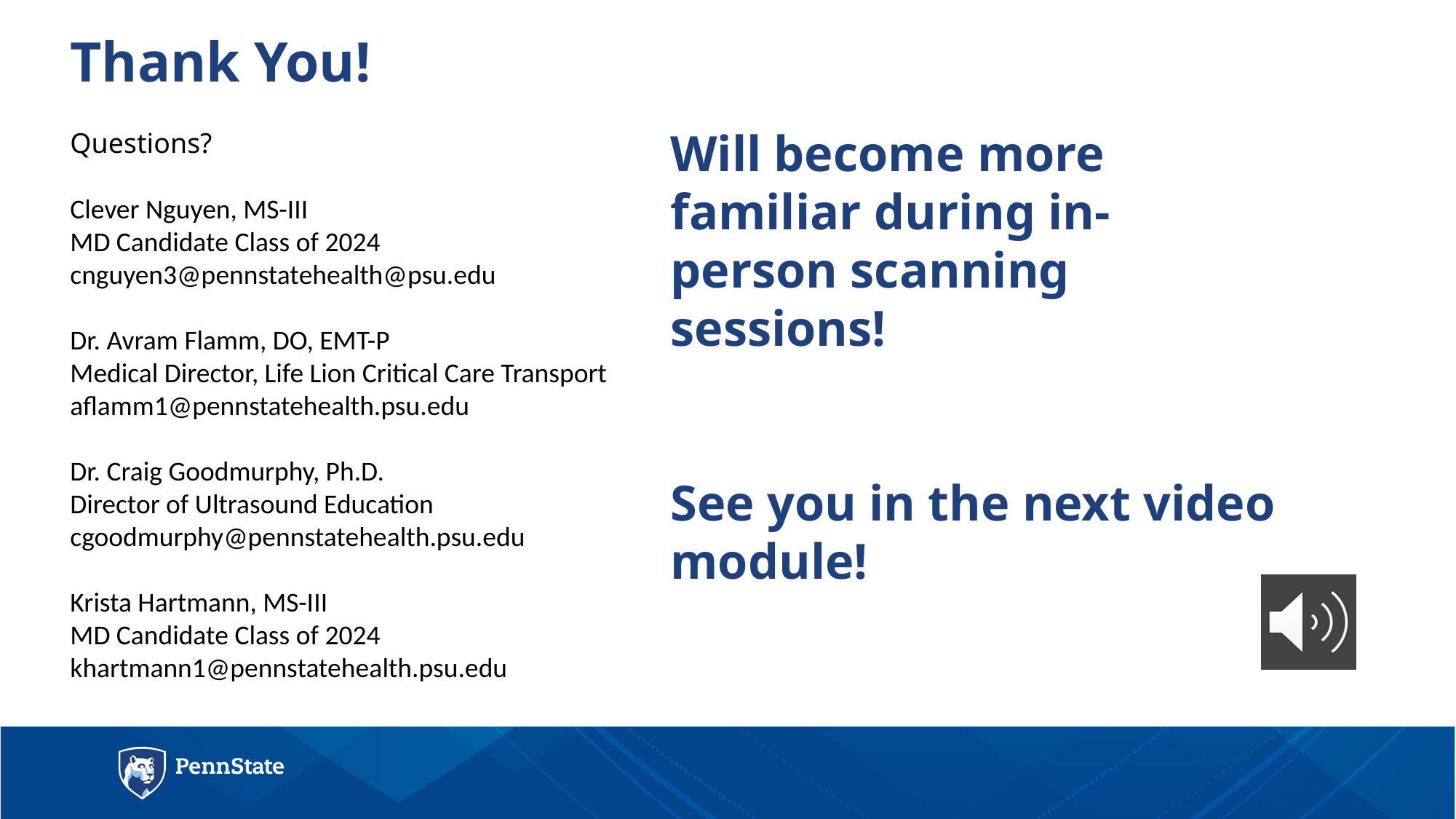

Thank You!
Questions?
Clever Nguyen, MS-III
MD Candidate Class of 2024
cnguyen3@pennstatehealth@psu.edu
Dr. Avram Flamm, DO, EMT-P
Medical Director, Life Lion Critical Care Transport
aflamm1@pennstatehealth.psu.edu
Dr. Craig Goodmurphy, Ph.D.
Director of Ultrasound Education
cgoodmurphy@pennstatehealth.psu.edu
Krista Hartmann, MS-III
MD Candidate Class of 2024
khartmann1@pennstatehealth.psu.edu
Will become more familiar during in-person scanning sessions!
See you in the next video module!
